# Direct synthesis of spirobifluorenes by formal dehydrative coupling of biaryls and fluorenones†

**DOI:** 10.1039/d3sc05977d

**Published:** 2024-01-02

**Authors:** Yugo Kato, Kazutoshi Nishimura, Yuji Nishii, Koji Hirano

**Affiliations:** a Department of Applied Chemistry, Graduate School of Engineering, Osaka University Suita Osaka 565-0871 Japan k_hirano@chem.eng.osaka-u.ac.jp; b Innovative Catalysis Science Division, Institute for Open and Transdisciplinary Research Initiatives (ICS-OTRI), Osaka University Suita Osaka 565-0871 Japan

## Abstract

A Tf_2_O-mediated, direct dehydrative coupling of (hetero)biaryls and fluorenones proceeds to form the corresponding spirobifluorenes in good to high yields. The reaction system allows the relatively simple nonhalogenated and nonmetalated starting substrates to be directly adopted in the spirocyclisation reaction. In addition, the double cyclisation reaction is easily performed, giving the highly spiro-conjugated aromatic compounds of potent interest in materials chemistry. The preliminary optoelectronic properties of some newly synthesised compounds are also demonstrated.

## Abstract 2

A dehydrative coupling of biaryls and fluorenones proceeds under TfOH- or Tf_2_O-promoted conditions to deliver the corresponding SBFs directly. This protocol is applicable to double cyclisation, giving the SBF-based highly π-conjugated molecules.

## Graphical abstract

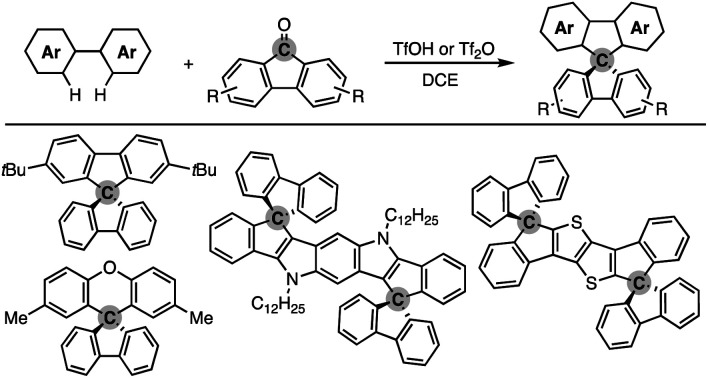

## Introduction

**Scheme 1:**
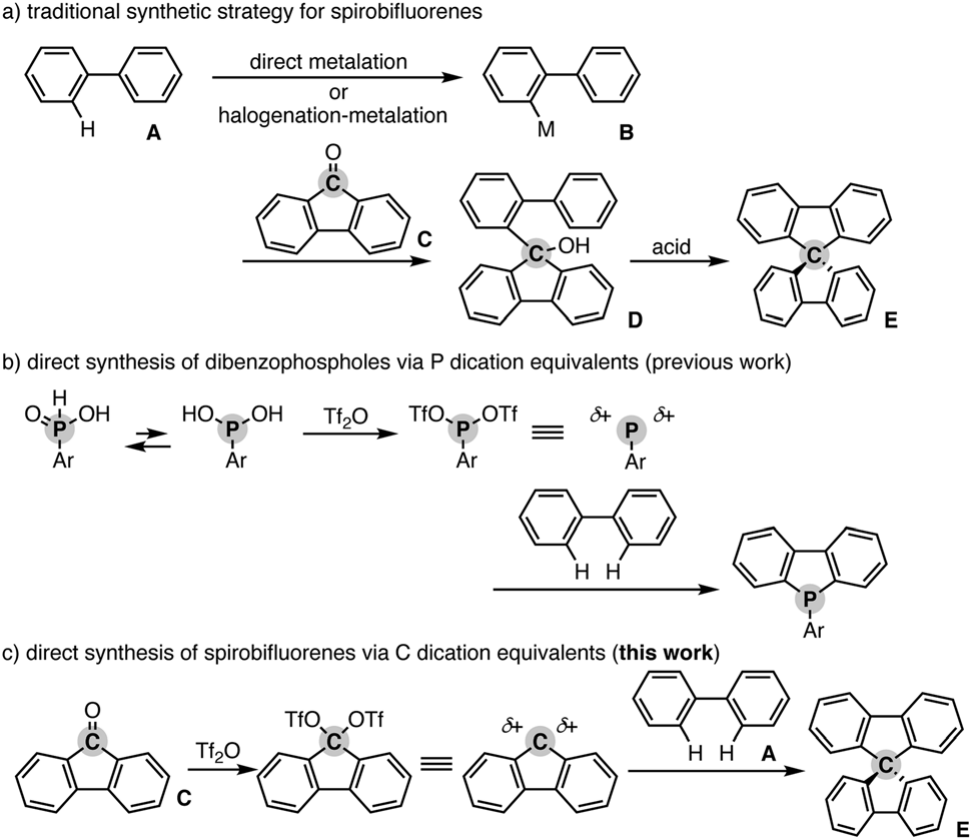
(a) General synthetic scheme of spirobifluorenes, (b) direct synthesis of dibenzophospholes from simple biaryls *via* P dication equivalents, and (c) working scenario of direct synthesis of spirobifluorenes from simple biaryls *via* C dication equivalents.

Spiro compounds have received significant attention in the research field of organic optoelectronics.^1^ In particular, spirobifluorene (SBF) is a promising structural candidate in the design of organic functional materials such as organic field-effect transistors,^2^ light-emitting diodes,^3^ and solar cells^4^ because of its unique homo-conjugation (spiro-conjugation) increasing the hole transporting ability^5^ and rigidity based on the perpendicular arrangement of two π systems that generally provides a high glass temperature (*T*_g_) for the preferable amorphous glassy state.^6^ Additionally, in specific cases, SBF has chirality at the spiro carbon and thus intriguing chiroptical properties for application as a circular polarized luminescence (CPL) material.^7^ Accordingly, numerous π-conjugated compounds incorporated with the SBF moiety have been designed and synthesised. However, their synthetic method still relies on tedious multistep sequences. In general, the starting simple biaryl A is metalated directly or indirectly *via* stepwise halogenation and metalation. The formed metalated biaryl B is then coupled with the fluorenone C to form the corresponding tertiary alcohol D. The final ring closure by the action of a Brønsted or Lewis acid produces the desired SBF E (Scheme 1a). Herein, we report a Tf_2_O-mediated formal dehydrative coupling of biaryls and fluorenones under relatively mild conditions (in DCE at 40–110 °C). By using this strategy, several hydrocarbon-based and heteroatom-incorporated SBFs are accessible directly without preparation and isolation of any halogenated and metalated intermediates. Moreover, the 1 : 2 couplings of biaryls and fluorenones are also possible, allowing the streamlined synthesis of largely π-extended molecules with two SBF moieties.

Our group recently developed a concise synthetic method for the preparation of dibenzophospholes from simple biaryls and phosphinic acids.^8^ The active species is the highly coordinately unsaturated phosphenium dication, which is generated from the phosphinic acid and Tf_2_O, and then readily reacts with the simple biaryl to form two C–P bonds in one synthetic operation (Scheme 1b). Inspired by the phosphenium dication chemistry, we envisioned a new synthetic route to the SBF E from the simple biaryl A and fluorenone C*via* a carbon dication: if the C=O function of fluorenone C is activated by Tf_2_O, the formed carbon ditriflate can work as a carbon dication equivalent to deliver the SBF E directly through double C–C bond formation with the simple bialy A (Scheme 1c).^9^ We also note that during the course of this study, Wang and Yang reported a related approach to spiro-acridans from fluorenones in the presence of *p*-TsOH as an activator, but the coupling partner was limited to diphenylamine, and the simple (hetero)biaryl was not tested.^10^ Moreover, a high reaction temperature (200 °C) was necessary to promote the reaction. Thus, our strategy is complementary to the work by Wang and Yang from the viewpoint of substrate scope.

## Results and discussion

**Scheme 2:**
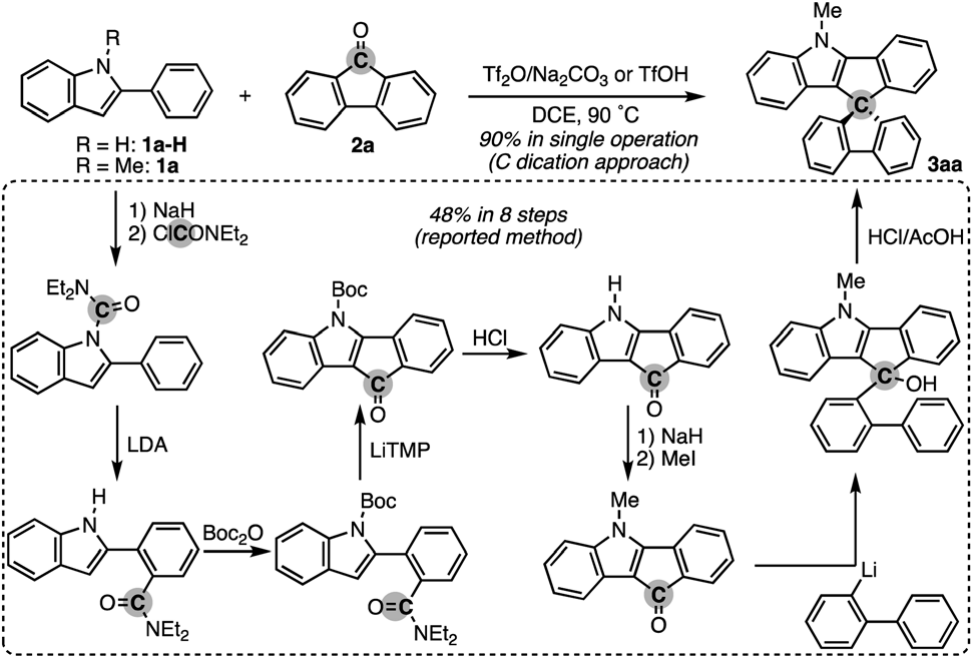
Comparison of the C dication equivalent strategy (top) *vs.* reported method (bottom) in the synthesis of *N*-incorporated spirobifluorene 3aa: Boc = *tert*-butoxycarbonyl, LDA = lithium diisopropylamide, and LiTMP = lithium tetramethylpiperidide.

To test the aforementioned hypothesis, we chose the synthesis of indole-containing SBF 3aa from 2-phenylindole 1a-H and fluorenone (2a) as the model reaction because the synthesis of 3aa was achieved but through a long-step sequence, including (1) *N*-carbamoylation, (2) LDA-mediated *N*-to-*C* carbamoyl migration, (3) *N*-Boc protection, (4) directed metalation/intramolecular acylation, (5 and 6) *N*-substituent switch from Boc to Me, (7) coupling with 2-lithiated biphenyl, and (8) ring closure using HCl/AcOH (48% total yield in 8 steps; Scheme 2; bottom route).^11^ In sharp contrast, our strategy is much more straightforward: upon treatment with 2a, Tf_2_O, and Na_2_CO_3_ in heated 1,2-dichloroethane (DCE), the *N*-methylated indole 1a (readily prepared and commercially available) was directly converted to 3aa in 90% yield (Scheme 2; top route). In this case, the simple Brønsted acid, TfOH, also promoted the reaction with comparable efficiency. This comparison scheme shows the considerable synthetic advantage of our reaction design based on the carbon dication equivalent (see the ESI† for detailed optimization studies).

**Scheme 3:**
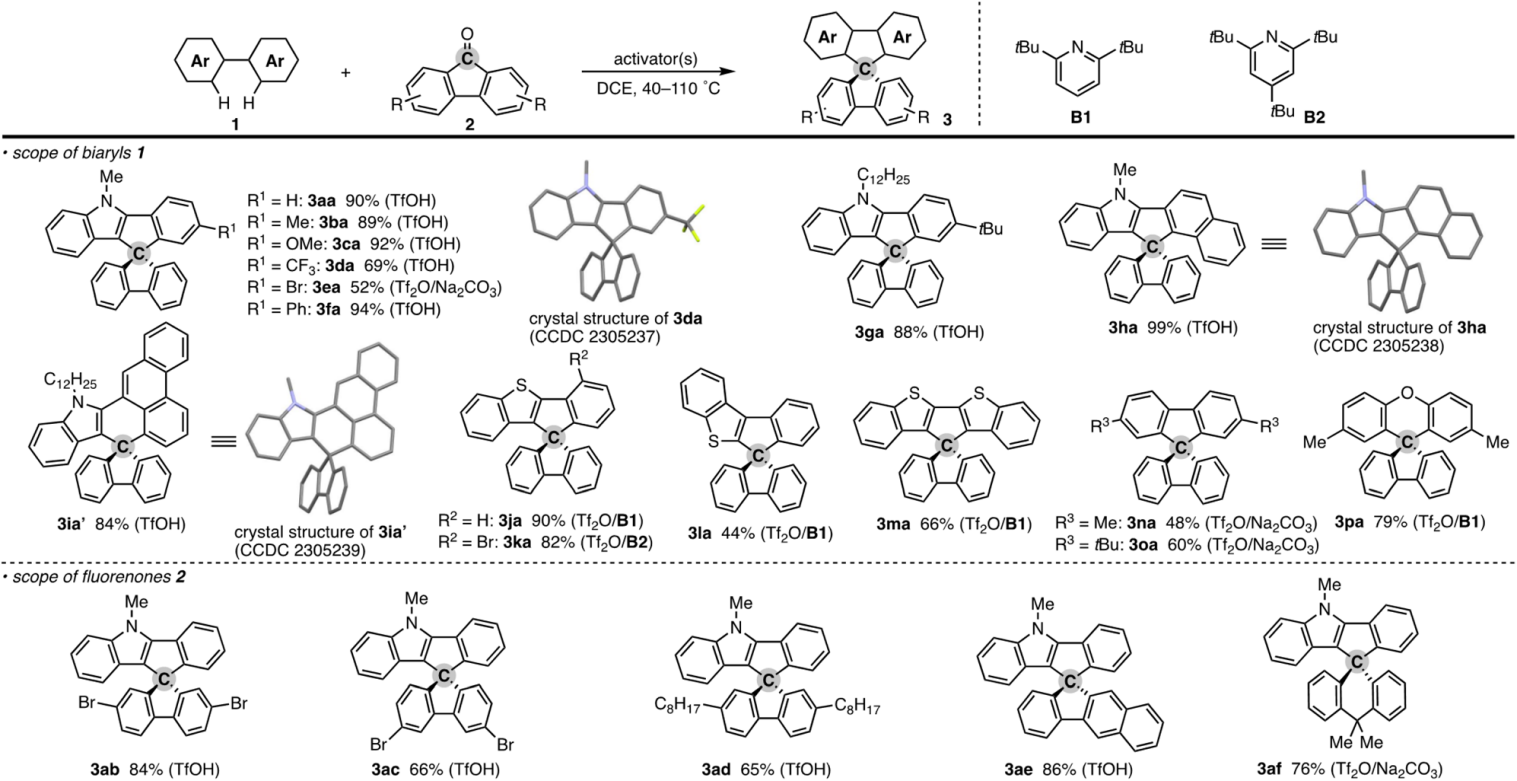
Products of formal dehydrative coupling of simple biaryls 1 and fluorenones 2. Isolated yields are shown. The activator(s) used is in parentheses. See the ESI† for detailed reaction conditions. In the crystal structures, hydrogens and long alkyl side chains are omitted for clarity.

**Scheme 4:**
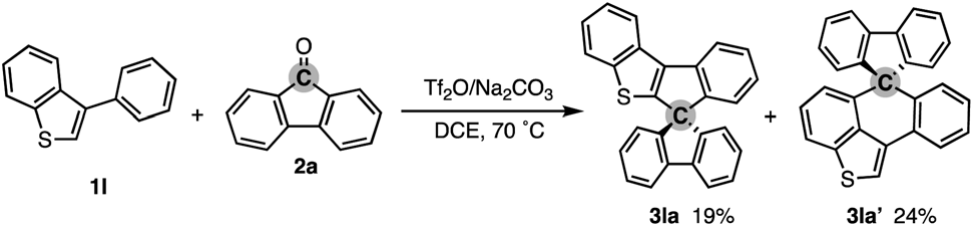
Competitive formation of 3la′ from 1l and 2a under Tf_2_O/Na_2_CO_3_ conditions.

To check the generality of our strategy, we first investigated the scope of biaryls 1 with fluorenone 2a (Scheme 3). The 2-arylindoles that bear both electron-donating and -withdrawing groups were compatible to form the corresponding *N*-containing SBFs 3ba–fa in one synthetic operation, where the electro-rich substrates showed better performance (3aa–ca, fa*vs.*3da–ea), reflecting the electrophilic substitution mechanism of this process. The structure of 3da was determined by X-ray crystallographic analysis (CCDC 2305237).† Additionally, the reaction conditions can obviate the reactive organometallic reagents such as organo-lithiums and -magnesiums, which were inevitable in literature methods,^1^ and thus 1e was transformed to 3ea with the Ar–Br moiety left intact, which can be a useful synthetic handle for further manipulation. This protocol was also tolerant of the longer alkyl chain on the nitrogen (3ga), which is known to generally enhance lipophilicity and solubility and to tune the aromatic π-stacking and π-conjugation of the corresponding oligomers and polymers. In the case of 2-naphthyl-substituted 1h, the cyclisation occurred selectively at the more sterically congested but more electron-rich C1 position (3ha; CCDC 2305238),† again indicating the electronically controlled substitution reaction. In contrast, the higher fused phenanthrene substrate exclusively formed the spiro[4.5] system 3ia′ over the SBF, which was confirmed by the X-ray analysis (CCDC 2305239).† Not only the 2-arylindoles but also several thiophene derivatives could be employed: intriguingly, in these cases the combination of Tf_2_O and sterically crowded 2,6-di(*tert*-butyl)pyridines (B1 and B2) uniquely promoted the reaction. For example, 2-arylbenzothiophenes 1j and 1k directly afforded the corresponding spirocycles 3ja and 3ka in 90 and 82% yields, respectively. Under conditions using Tf_2_O/Na_2_CO_3_ or TfOH, the yields decreased by *ca.* 30% (data not shown). In the reaction of the regioisomeric 3-phenylbenzothiophene (1l), the effect of the pyridine base was more remarkable: B1 provided the desired SBF 3la exclusively, while the inorganic Na_2_CO_3_ base formed a mixture of 3la and its constitutional isomer 3la′ (Scheme 4). The results suggest the involvement of the pyridine base in the C–C bond forming step.^12^ Moreover, 2,2′-bisbenzothiophene-based SBF 3ma was readily accessible under the Tf_2_O/B1 conditions. Particularly notable is the successful use of simple 4,4′-dimethyl- and di(*tert*-butyl)biphenyls to furnish the corresponding spiro hydrocarbons 3na and 3oa in synthetically useful yields. Furthermore, the reaction accommodated the synthesis of spiro[fluorene-9,9′-xanthene] from the diaryl ether (3pa), which receives significant attention as the core structure in the design of organic electronics and light emitting devices.^13^

In addition to the parent 2a, several substituted fluorenones were coupled with 1a: 2,7- and 3,6-dibromofluorenones both were viable to deliver the brominated 3ab and 3ac in good yields. In particular, the latter substitution pattern is generally difficult to obtain by the conventional electrophilic bromination of the simple SBF.^1,14^ The long alkyl side chain on the fluorenone was also amenable (3ad). The reaction of benzofluorenone proceeded smoothly to enable the facile construction of chiral SBF 3ae. Additionally, the xanthone derivative could also be used, thus providing access to the spiro[4.5] system 3af.^15^

**Fig. 1:**
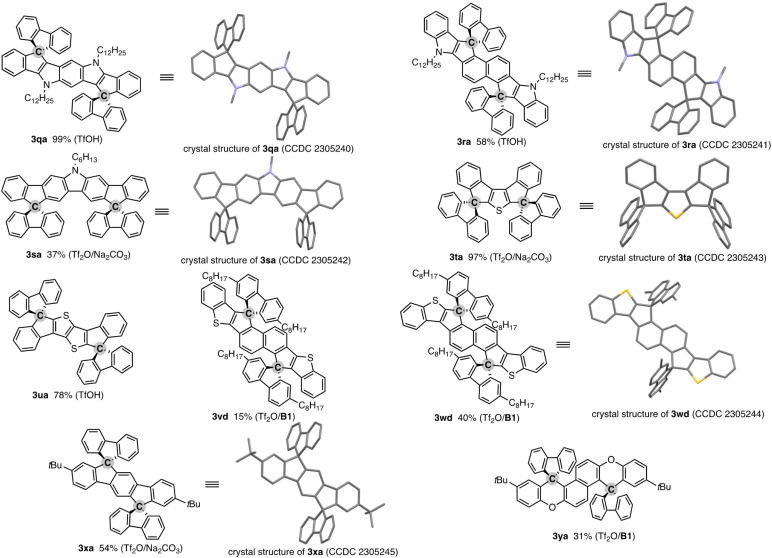
Products of formal dehydrative coupling of simple biaryls 1 and fluorenones 2. Isolated yields are shown. The activator(s) used is in parentheses. See the ESI† for detailed reaction conditions. In the crystal structures, hydrogens and long alkyl side chains are omitted for clarity.

The salient advantage of this concise and one-pot strategy is demonstrated more clearly in the attempt to synthesise more conjugated doubly spirocyclic compounds (Fig. 1). The coupling of 2,6-diphenylpyrroloindole 1q and two equivalent of fluorenone (2a) was promoted by simple treatment with TfOH, and the corresponding double spirocycle 3qa was isolated in a nearly quantitative yield. The 2,6-bis(indole)naphthalene 1r also reacted with 2a regioselectively at the C1 and C5 positions under the same conditions to furnish 3ra as the single isomer in 57% yield. The carbazole also worked as the reactive *N*-heteroaromatic core, and the two-SBF-fused pyrrole 3sa was formed in the single operation. The structures of three *N*-containing double spirocycles were unambiguously determined by X-ray crystallographic analysis (CCDC 2305240, 2305241, and 2305242).† Promising reactivity was also observed in the synthesis of thiophene-incorporated derivatives: 3,4-diphenylthiophene 1t was readily converted to 3ta in 97% yield (CCDC 2305243).† The synthetic route to 3ta from 1t was reported, but it required multistep sequences involving stepwise lithiation or stoichiometric halogenation/metalation and suffered from low to moderate overall yield (25–41%).^16^ Thus, the straightforward access to 3ta further shows the beneficial point of our protocol. Moreover, the thienothiophene 1u and benzothiophene end-capped naphthalenes 1v and 1w, which are candidates of the core structure of organic field-effect transistors, could be easily decorated by two-fold spirocyclisation to produce 3ua, 3vd, and 3wd (CCDC 2305244).† A similar 1 : 2 coupling of simple aromatic hydrocarbon, terphenyl 1x, and fluorenone (2a) was also possible, giving 3xa with exclusive regioselectivity (CCDC 2305245).† While a related structural motif was generally prepared from prefunctionalised terphenyls such as diiodoterphenyls and indenofluorenedione,^17^ the present carbon dication strategy enables the direct use of relatively simple precursor 1x. The double spirocyclisation reaction was applicable to the diaryloxynaphthalene 1y, and fused spirofluorenexanthene 3ya of potent interest in materials chemistry^13^ was obtained regioselectively in a synthetically acceptable yield.

**Fig. 2:**
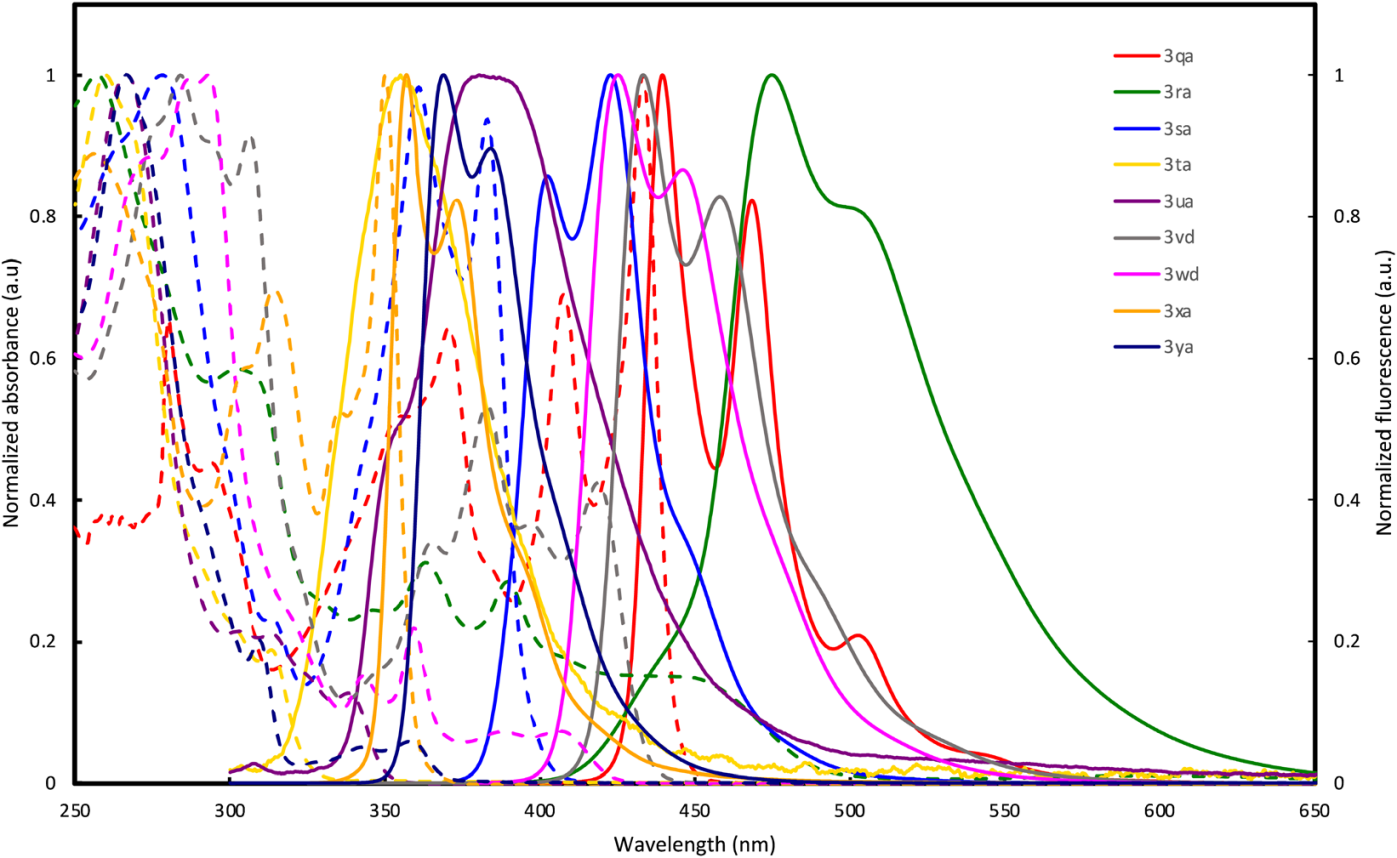
UV-vis absorption (dashed line) and fluorescence spectra (solid line) of 3qa, 3ra, 3sa, 3ta, 3ua, 3vd, 3wd, 3xa, and 3ya in solution (1.0 × 10^−5^ M).

The optical properties of double spirocycles obtained in Fig. 1 were preliminary surveyed in a solution state (1.0 × 10^−5^ M CHCl_3_ solution). Only for the pyrroloindole 3qa, the spectra were obtained in diluted toluene solution due to its instability in CHCl_3_. The results are summarized in Fig. 2 and Table 1. In the *N*-containing systems (3qa–3sa), relatively high quantum yields were obtained probably because of the effective conjugation and rigidity based on the spiro system. In particular, 3qa and 3ra showed the absorption peaks in the visible light region and relatively strong blue fluorescence with maxima at *λ* = 440 and 469 (3qa) and 475 (3ra) nm, respectively. On the other hand, thiophene and thienothiophene derivatives 3ta and 3ua resulted in poor quantum yields, but the higher fused bisbenzothiophene derivatives 3vd and 3wd improved the performance. Additionally, while the figures of both absorption and emission spectra are similar, 3vd has more intensive absorbance in the long-wavelength region than 3wd, thus suggesting that the direction of the benzothiophene fusion largely affects photoluminescence efficiency. The hydrocarbon 3xa also resulted in a relatively high quantum yield owing to the incorporation of the perpendicular SBF architecture.

**Table 1:** Optical properties of 3qa, 3ra, 3sa, 3ta, 3ua, 3vd, 3wd, 3xa, and 3ya^a^

3	*λ*_abs_ (nm) (*ε* (10^4^ M^−1^ cm^−1^))	*λ*_Fl_ b (nm)	*Φ* (%)
3qa	280 (3.1), 371 (3.0), 408 (3.2), 433 (4.7)	440, 469	83
3ra	303 (3.2), 363 (1.7), 390 (1.6), 447 (0.83)	475	39
3sa	278 (6.1), 361 (6.0), 383 (5.7)	403, 423	49
3ta	260 (4.8), 313 (0.90)	355	4
3ua	266 (7.5), 303 (1.6), 313 (1.6), 338 (0.96)	381	5
3vd	284 (8.0), 306 (7.4), 365 (2.7), 383 (4.3), 397 (2.9), 419 (3.4)	434, 458	48
3wd	287 (9.6), 293 (9.7), 343 (1.5), 359 (2.1), 388 (0.70), 406 (0.71)	425, 446	20
3xa	305 (2.1), 314 (2.5), 335 (1.9), 350 (3.6)	357, 373	43
3ya	266 (7.3), 309 (1.5), 343 (0.37), 358 (0.43)	369, 384	19

a Measured in 1.0 × 10^−5^ M solution of toluene (3qa) and CHCl_3_ (3ra–3ya).

b Excited at *λ*_abs_.

We also examined the electrochemical properties of the *N*- and *S*-containing doubly cyclised SBFs by cyclic voltammetry (CV) in *o*-dichlorobenzene/MeCN (10/1, v/v) with tetrabutylammonium hexafluorophosphate (Bu_4_NPF_6_) as an electrolyte *versus* ferrocene/ferrocenium ions (Fc/Fc^+^) (Fig. S23–29†), and their HOMO and LUMO levels were estimated according to the first oxidation potentials and the optical band gaps (*E*^opt^_g_). The data are summarized in Table 2. The CV of 3qa, 3ra, 3vd, and 3wd showed two-step, reversible oxidation waves associated with their two pyrrole (3qa and 3ra) and thiophene rings (3vd and 3wd), respectively, and their *E*_ox_^1/2^ values are relatively shifted in a negative direction. In particular, the first oxidation potential of 3qa was significantly lower, which is reflected by the highly rigid and electron-rich two-indene-fused pyrroloindole core. On the other hand, the HOMO–LUMO energy gap is relatively narrow for the two-indole-fused octacyclic system 3ra, suggesting its larger intramolecular charge transfer ability. Both the HOMO and LUMO levels of regioisomeric 3vd and 3wd are almost the same. Thus, in contrast to the photoluminescent properties (Table 1), their electrochemical properties are less dependent on the thiophene ring orientation.

**Table 2:** Absorption wavelengths, HOMO–LUMO energy gaps and cyclic voltammogram data of compounds 3qa, 3ra, 3sa, 3ta, 3ua, 3vd, and 3wd

3	*λ*_onset_^abs^ a (nm)	*E* ^opt^_g_ b (eV)	*E*_ox_^1/2^ c (V)	*E*_HOMO_ d (eV)	*E*_LUMO_ e (eV)
3qa	440	2.82	0.0085	−4.81	−1.99
3ra	480	2.58	0.309	−5.11	−2.53
3sa	401	3.09	0.665	−5.47	−2.38
3ta	328	3.78	1.05	−5.85	−2.07
3ua	348	3.56	0.843	−5.64	−2.08
3vd	432	2.87	0.647	−5.44	−2.58
3wd	420	2.95	0.628	−5.43	−2.48

a Measured in toluene (3qa) and CHCl_3_ (3ra–3wd).

b Determined from the onset of the normalized absorption spectra.

c Performed in *o*-dichlorobenzene/MeCN (10 : 1, v/v) in the presence of Bu_4_NPF_6_. *v* = 10.0 mV s^−1^ (3qa, 3ra, and 3sa), 5.0 mV s^−1^ (3ta, 3ua, and 3vd), and 4.0 mV s^−1^ (3wd), *versus* Fc/Fc^+^.

d The approximation for the Fc/Fc^+^ level is −4.8 eV *versus* vacuum: *E*_HOMO_ = −4.8 − *E*_ox_^1/2^.

e Estimated from *E*_HOMO_ and *E*^opt^_g_: *E*_LUMO_ = *E*_HOMO_ + *E*^opt^_g_.

## Conclusions

We have developed a formal dehydrative coupling reaction of (hetero)biaryls and fluorenone derivatives. The reaction proceeds well under metal-free TfOH- or Tf_2_O-promoted conditions, in which two C–C bonds are sequentially formed and the corresponding spirobifluorenes (SBFs) are obtained directly. This protocol does not necessitate any halogenated and metalated starting substrates/intermediates and thus is quite simple, practical, and beneficial from the viewpoint of functional group compatibility. Actually, the reaction was tolerant of the Ar–Br moiety, which can be further functionalised by the established cross-coupling chemistry. Moreover, the present reaction can be applicable to double cyclisation to deliver largely π-conjugated SBFs in one synthetic operation. Given their unique homo-conjugation and rigidity based on the perpendicular arrangement of two π systems, the obtained doubly spiro SBFs are of potent interest in materials chemistry. Further expansion of substrate scope, development of catalytic conditions, and application to chiral SBF molecules are ongoing in our laboratory.

## Data availability

All experimental procedures and spectroscopic data can be found in the ESI.†

## Author contributions

K. N. and K. H. conceived the idea. Y. K. and K. N. performed all experiments. Y. N. assisted with X-ray analysis. K. H. supervised the project and wrote the manuscript. All the authors discussed the results and commented on the manuscript.

## Conflicts of interest

There are no conflicts to declare.

## Supplementary Material

SC-015-D3SC05977D-s001

SC-015-D3SC05977D-s002

## References

[cit1] Saragi T. P. I., Spehr T., Siebert A., Fuhrmann-Lieker T., Salbeck J. (2007). Chem. Rev..

[cit2] Saragi T. P. I., Fuhrmann-Lieker T., Salbeck J. (2005). Synth. Met..

[cit3] Steuber F., Staudigl J., Stössel M., Simmerer J., Winnacker A., Spreitzer H., Weissörtel F., Salbeck J. (2000). Adv. Mater..

[cit4] Hawash Z., Ono L. K., Qi Y. (2018). Adv. Mater. Interfaces.

[cit5] Schweig A., Weidner U., Hellwinkel D., Krapp W. (1973). Angew. Chem., Int. Ed..

[cit6] Naito K., Miura A. (1993). J. Phys. Chem..

[cit7] Takase K., Noguchi K., Nakano K. (2017). Org. Lett..

[cit8] Nishimura K., Hirano K., Miura M. (2020). Org. Lett..

[cit9] (a) Olah G. A. , Surya Prakash G. K., Molnár Á. and Sommer J., Superacid Chemistry, John Wiley & Sons, 2nd edn, 2009

[cit10] Liu H., Liu Z., Li C., Huang H., Zhou C., Wang Z., Yang C. (2021). Angew. Chem., Int. Ed..

[cit11] Cao J. , Hua R. and Wang S., *Chinese Pat.*, CN 104557875A, 2015

[cit12] The interconversion between 3la and 3la′ did not occur in the presence of Tf_2_O/B1 or Tf_2_O/Na_2_CO_3_, thus indicating that both compounds were kinetically formed. See the ESI† for details

[cit13] Gu J.-F., Xie G.-H., Zhang L., Chen S.-F., Lin Z.-Q., Zhang Z.-S., Zhao J.-F., Xie L.-H., Tang C., Zhao Y., Liu S.-Y., Huang W. (2010). J. Phys. Chem. Lett..

[cit14] Tour J. M., Wu R., Schumm J. S. (1990). J. Am. Chem. Soc..

[cit15] The cyclic structure such as fluorenones and xanthones was essential: when the simple benzophenone was used, the direct construction of spiro carbon did not occur, and the corresponding tertiary alcohol was observed as the sole product. We also tried the reaction of *N*-Boc-2-phenylindole under optimal conditions. However, the Boc protection was unstable and underwent the deprotection and/or transamidation with Tf_2_O to deliver a mixture of N–H and N–Tf spiroindole compounds in <10% combined yield

[cit16] Xie L.-H., Hou X.-Y., Tang C., Hua Y.-R., Wang R.-J., Chen R.-F., Fan Q.-L., Wang L.-H., Wei W., Peng B., Huang W. (2006). Org. Lett..

[cit17] Horhant D., Liang J.-J., Virboul M., Poriel C., Alcaraz G., Rault-Berthelot J. (2006). Org. Lett..

