# Supplementary material for: Direct synthesis of spirobifluorenes by formal dehydrative coupling of biaryls and fluorenones
Source: Chem Sci. 2024 Jan 2;15(6):2112–7. doi: 10.1039/d3sc05977d (PMC10848674; doi:10.1039/d3sc05977d)

Electronic Supplementary Material (ESI) for Chemical Science.
This journal is © The Royal Society of Chemistry 2024

Supplementary Information

Direct synthesis of spirobifluorenes by formal dehydrative coupling of biaryls and fluorenones

Yugo Kato,[†] Kazutoshi Nishimura,[†] Yuji Nishii,[†] and Koji Hirano*[,†, ‡]

[†]*Department of Applied Chemistry, Graduate School of Engineering, Osaka University, Suita, Osaka 565-0871, Japan*

[‡]*Innovative Catalysis Science Division, Institute for Open and Transdisciplinary Research Initiatives (ICS-OTRI), Osaka University, Suita, Osaka 565-0871, Japan*

k_hirano@chem.eng.osaka-u.ac.jp

Contents

**Instrumentation and Chemicals**	S1–S2
**Pictures of Reaction Set-Up**	S3–S4
**Experimental Procedures for Starting Substrates**	S5–S9
**Detailed Optimization Studies**	S10–S11
**X-Ray Analysis**	S12–S20
**Tests for Possibility of Interconversion between 3la and 3la'**	S21
**Table of Detailed Conditions for Each Substrate**	S22
**Photoluminescence Properties**	S23–S27
**Cyclic Voltammetry**	S28–S31
**Characterization Data and Experimental Procedures for Products**	S32–S56
**References**	S57
**Copies of NMR Charts**	S58–S91

Instrumentation and Chemicals

^{1}H, $^{13}C\{^{1}H\}$, and $^{19}F\{^{1}H\}$ spectra were recorded at 400 MHz, 100 MHz, and 162 MHz, respectively, for $CDCl_3$ or CD_2Cl_2 solutions. HRMS data were obtained by APCI and FAB using a TOF and a magnetic sector, respectively. GC analysis was carried out using a silicon OV-17 column (i. d. 2.6 mm x 1.5 m) or CBP capillary column (i. d. 0.5 mm x 25 m). TLC analyses were performed on commercial glass plates bearing 0.25-mm layer of Merck Silica gel $60F_{254}$. Silica gel (Wakosil C-200,

Wako Pure Chemical Co.) was used for column chromatography. Gel permeation chromatography (GPC) was performed by LC-20AR (pump, SHIMADZU, 7.5 mL/min $CHCl_3$) and SPD-20A (UV detector, SHIMADZU, 254 nm) with two in-line YMC-GPC T2000 (20 x 600 mm, particle size: 10 μm) (preparative columns, YMC). UV-vis spectra were acquired with JASCO V-750 spectrometer. Photoluminescence spectra and quantum yield measurements were conducted with JASCO FP-8500 spectrometer equipped with an integration sphere system. Cyclic voltammogram (CV) was recorded on ALS Electrochemical Analyzer Model 600E equipped with SVC-3 Voltammetry cell. Counter and working electrodes were made of Pt, and the reference electrode was Ag/Ag^+. The measurements were conducted in *o*-dichlorobenzene/MeCN solvent (10/1, v/v) containing tetrabutylammonium hexafluorophosphate as a supporting electrolyte at an indicated scan rate. All the potentials were calibrated with the standard ferrocene/ferrocenium (Fc/Fc^+) redox couple measured in identical conditions. The IUPAC convention was used to report the CV data.

Unless otherwise noted, materials obtained from commercial suppliers were used without further purification. *N*-Methyl-2-phenylindole (**1a**), biphenyls **1n**–**o**, di(*p*-tolyl) ether (**1p**), and fluorenones **2** except for **2d** were commercially available. DCE was freshly distilled from CaH_2. All reactions were carried out under nitrogen atmosphere unless otherwise noted.

Pictures of Reaction Set-Up

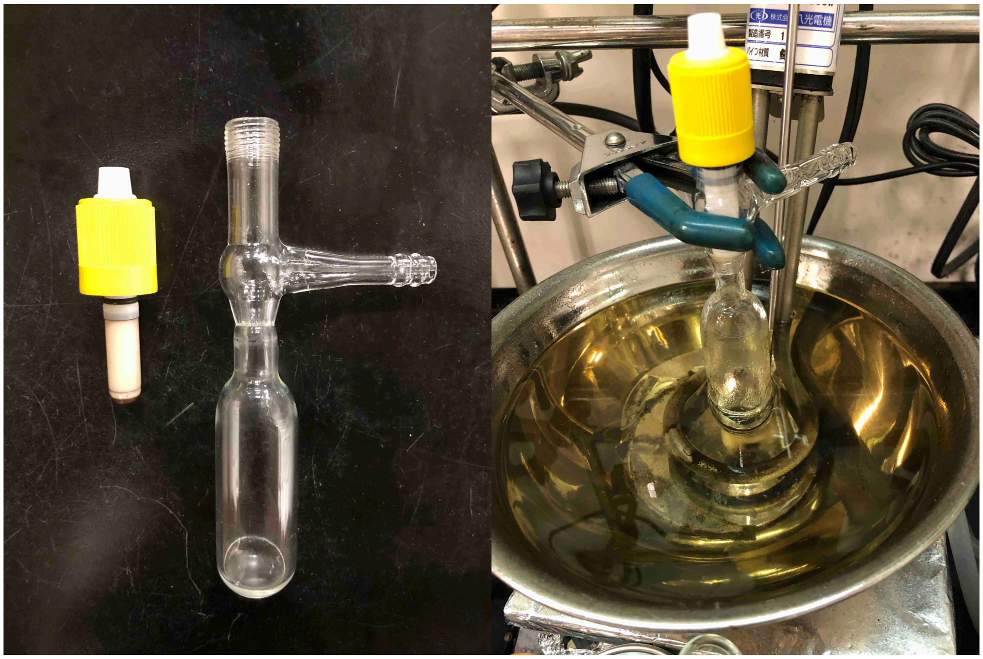

Figure S1. Photos of a schlenk flask used in this study: reaction set up (left); reaction progress (right).

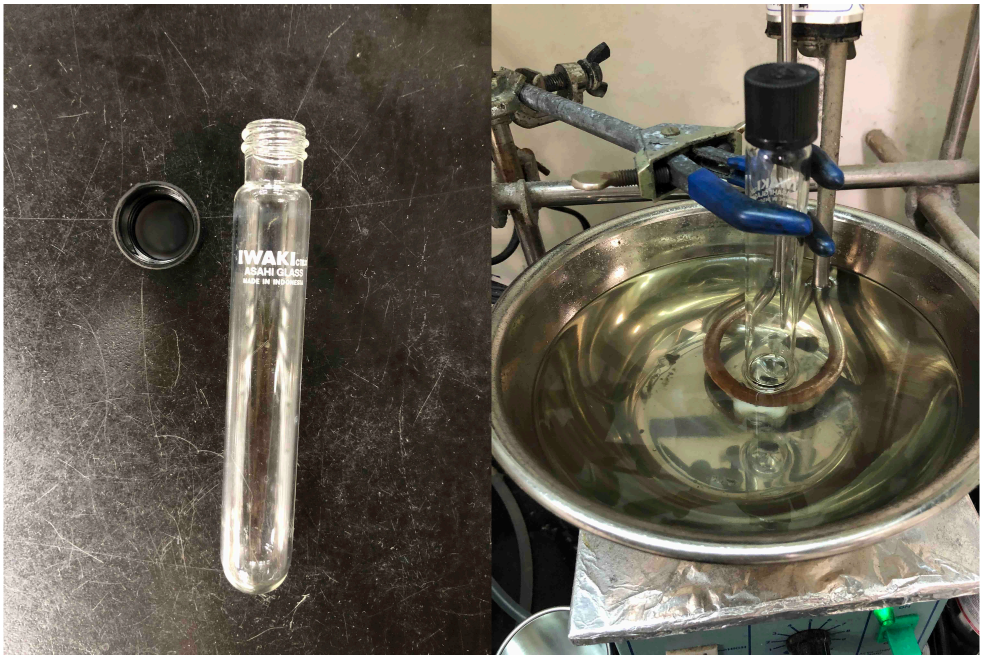

Figure S2. Photos of a 20 mL screw cap test tube used in this study: reaction set up (left); reaction progress (right).

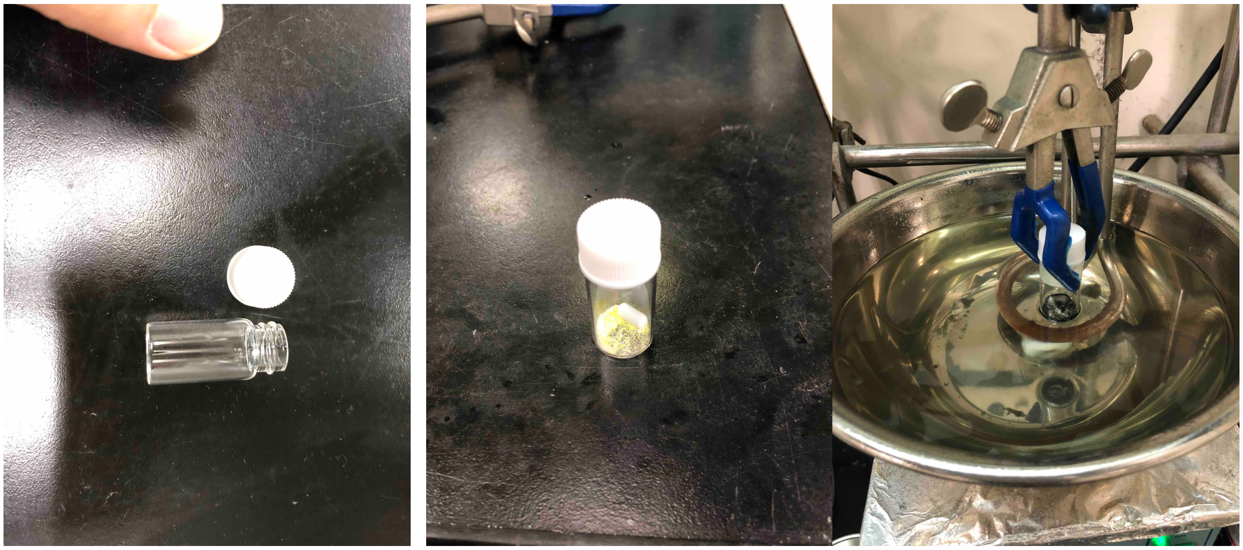

Figure S3. Photos of a 18*40 mm screw vial used in this study: reaction set up (left and center); reaction progress (right).

Experimental Procedures for Starting Substrates

The following starting substrates were synthesized according to the literature methods (Figure S4).

• Prepared by Fischer indole synthesis[S1]

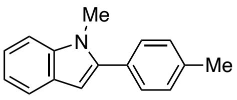

1b

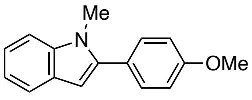

1c

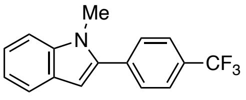

1d

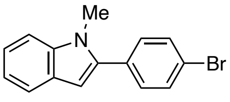

1e

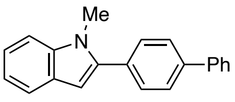

1f

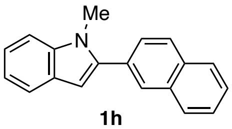

1h

• Prepared by C–H arylation[S2]

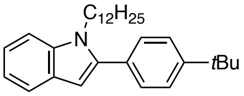

1g

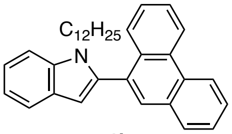

1i

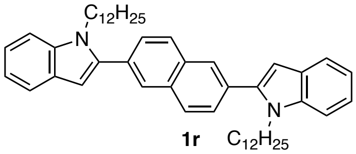

1r

• Prepared by Suzuki-Miyaura coupling[S3]

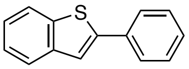

1j

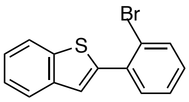

1k

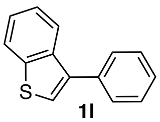

1l

• Prepared by homocoupling[S4]

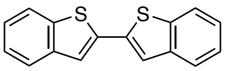

1m

• Prepared by Suzuki-Miyaura coupling[S5]

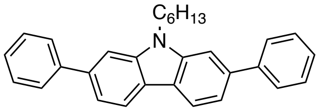

1s

• Prepared by Suzuki-Miyaura coupling[S6]

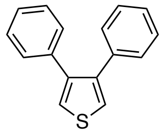

1t

• Prepared by Suzuki-Miyaura coupling[S7]

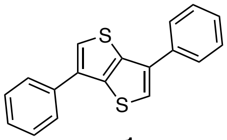

1u

• Prepared by Suzuki-Miyaura coupling[S8]

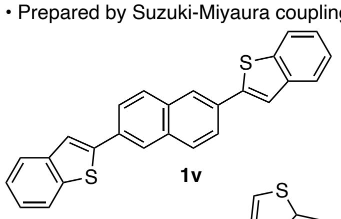

1v

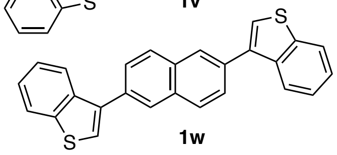

1w

• Prepared by Suzuki-Miyaura coupling[S9]

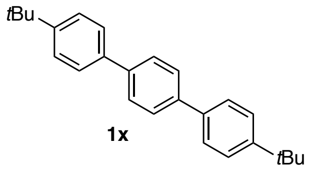

1x

• Prepared by C–O coupling[S10]

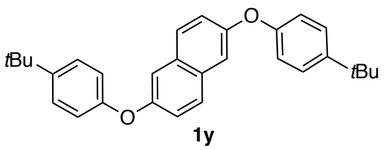

1y

Figure S4. Starting substrates prepared according to the literature methods.

Synthesis of **1q** (Scheme S1).

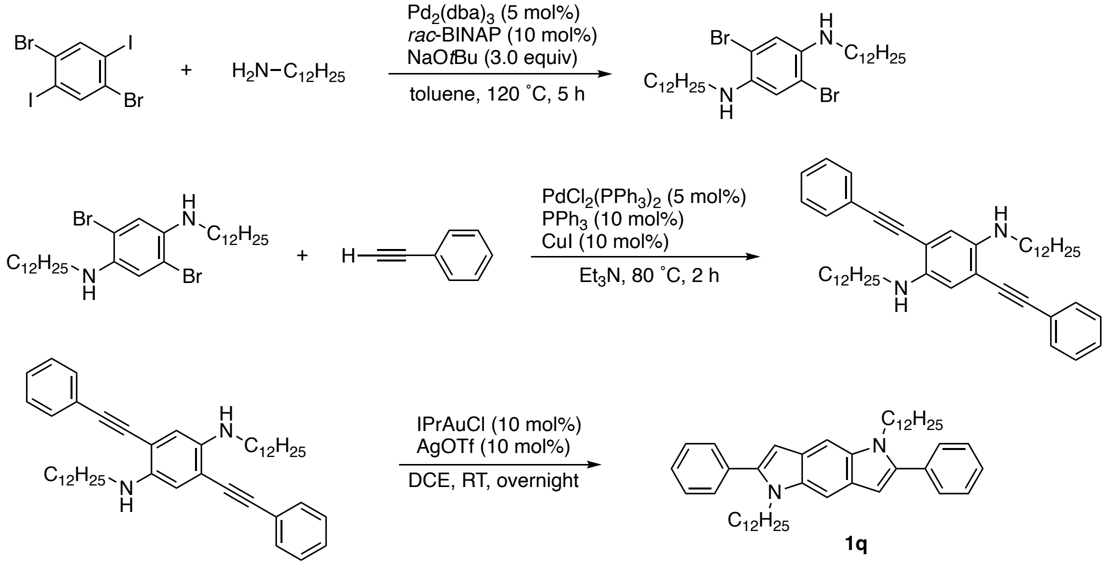

Scheme S1. Synthetic scheme of **1q**.

In a 200 mL two necked flask, 1,4-dibromo-2,5-diiodobenzene (10 mmol, 4.9 g), dodecylamine (26 mmol, 4.8 g), $Pd_2(dba)_3$ (0.50 mmol, 0.46 g), *rac*-BINAP (1.0 mmol, 0.62 g), and NaO*t*Bu (30 mmol, 2.9 g) were placed with a magnetic stir bar. The reaction flask was vacuumed and refilled with dry N_2. Toluene (100 mL) was subsequently added by a syringe. The reaction mixture was heated at 120 ˚C for 5 h (oil bath). After being cool to room temperature, the resulting mixture was filtered through a short pad of silica gel, and the filtrate was evaporated in vacuo. The residual solid was dissolved in heated ethyl acetate. Addition of isopropyl alcohol at room temperature allowed precipitation of pale yellow solid, which was collected and dried under reduced pressure. The desired 2,5-dibromo-N^1,N^4-didodecylbenzene-1,4-diamine (1.8 mmol, 1.1 g) was obtained in 18% yield.[S11]

In a 20 mL two necked flask, 2,5-dibromo-N^1,N^4-didodecylbenzene-1,4-diamine (1.8 mmol, 1.1 g), 1,4-dibromo-2,5-diiodobenzene (10 mmol, 4.9 g), $PdCl_2(PPh_3)_2$ (0.090 mmol, 63 mg), PPh_3 (0.18 mmol, 47 mg), and CuI (0.18 mmol, 34 mg) were placed with a magnetic stir bar. The reaction flask was vacuumed and refilled with dry N_2. Degassed Et_3N (4.0 mL) and phenylacetylene (4.2 mmol, 0.46 mL) were subsequently added by syringes. The reaction mixture was heated at 80 ˚C for 2 h (oil bath). After being cool to room temperature, the resulting mixture was quenched with H_2O and extracted with chloroform (100 x 3). The combined organic layers were evaporated under reduced pressure. The residual oil was dissolved in toluene, and the solution was filtered through a pad of Na_2SO_4 and neutral

alumina. The filtrate was evaporated again to form yellow reddish brown solid. The solid was dissolve in chloroform. Addition of isopropyl alcohol and gentle evaporation allowed precipitation of orange solid, which was collected and dried to deliver the desired N^1,N^4-didodecyl-2,5-bis(phenylethynyl)benzene-1,4-diamine (1.3 mmol, 0.84 g) as orange solid in 74% yield.[S11]

In a 20 mL two necked flask, N^1,N^4-didodecyl-2,5-bis(phenylethynyl)benzene-1,4-diamine (0.65 mmol, 0.42 g), IPrAuCl (0.065 mmol, 40 mg), and AgOTf (0.065 mmol, 17 mg) were placed with a magnetic stir bar. The reaction flask was vacuumed and refilled with dry N_2. DCE (7.0 mL) was subsequently added by a syringe. The reaction mixture was stirred at room temperature overnight. The resulting mixture was directly filtered through a pad of neutral alumina, and the filtrate was evaporated in vacuo. The residual solid was dissolved in chloroform, and addition of isopropyl alcohol allowed precipitation of pale yellow solid, which was collected and rinsed with cold hexane. Dryness under reduced pressure gave 1,5-didodecyl-2,6-diphenyl-1,5-dihydropyrrolo[2,3-*f*]indole (**1q**, 0.62 mmol, 0.38 g) as white solid in 95% yield.[S12]

1,5-Didodecyl-2,6-diphenyl-1,5-dihydropyrrolo[2,3-*f*]indole (**1q**). white solid; m.p. 111.6-113.6 °C; ^{1}H NMR (CD_2Cl_2, 400 MHz): δ 7.57-7.55 (m, 4H), 7.52 (s, 2H), 7.49 (dd, J = 7.2, 7.2 Hz, 4H), 7.42-7.38 (m, 2H), 6.56 (s, 2H), 4.19 (t, J = 7.5 Hz, 4H), 1.75-1.71 (m, 4H), 1.25-1.17 (m, 36H), 0.88 (t, J = 6.7 Hz, 6H). ^{13}C{^{1}H} NMR (CD_2Cl_2, 100 MHz): δ 142.6, 135.6, 134.1, 129.6, 128.8, 128.0, 126.5, 101.4, 99.5, 44.6, 32.3, 30.0 (2C), 29.93, 29.87, 29.8, 29.7, 29.5, 27.2, 23.1, 14.3. HRMS (APCI) m/z $(M+H)^+$ calcd for $C_{46}H_{65}N_2$: 645.5142, found: 645.5141.

Synthesis of **2d** (Scheme S2).

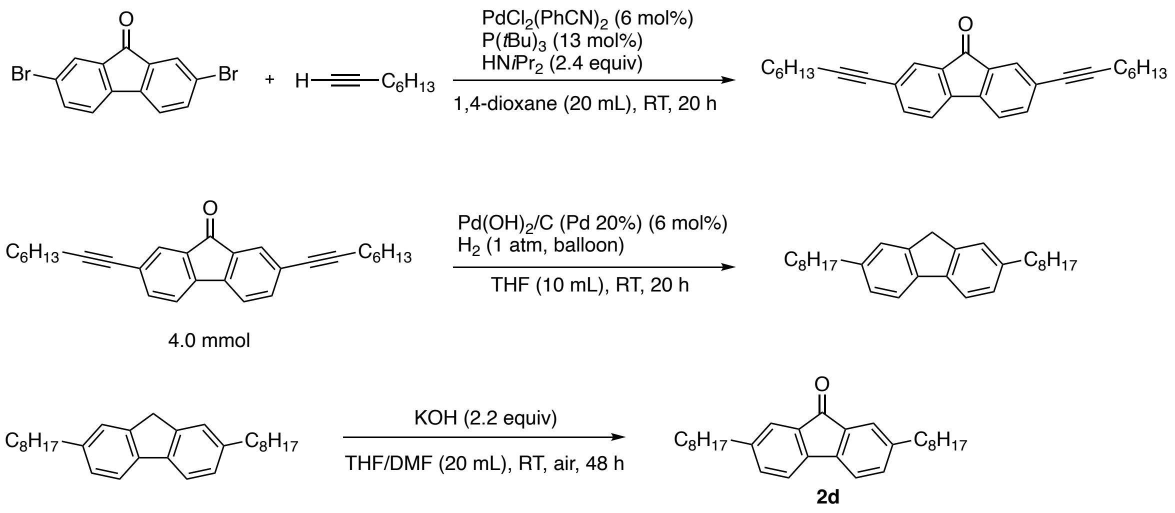

Scheme S2. Synthetic scheme of **2d**.

In a 50 mL two necked flask, 2,7-dibromofluorene (10 mmol, 3.4 g) and $PdCl_2(PhCN)_2$ (0.60 mmol, 0.23 g) were placed with a magnetic stir bar. The reaction vessel was vacuumed and refilled with dry N_2. 1,4-Dioxane (20 mL), P(*t*Bu)$_3$ (1.3 mmol, 0.26 g), diisopropylamine (24 mmol, 2.4 g, 3.4 mL), and 1-octyne (24 mmol, 2.6 g) were subsequently added by a syringe. The reaction mixture was stirred at room temperature for 20 h. The resulting mixture was diluted with H_2O, extracted three times with $CHCl_3$ (20 mL x 3), dried over Na_2SO_4, filtered through a pad of silica gel. The crude mixture was concentrated in vacuo. The desired product 2,7-di(oct-1-yn-1-yl)-9*H*-fluoren-9-one (4.0 mmol, 1.6 g, 40%) was isolated by column chromatography on silica gel using hexane/toluene (1/0 to 1/1, v/v) as eluent followed by GPC (chloroform).

In a 20 mL two necked flask, a mixture of 2,7-di(oct-1-yn-1-yl)-9*H*-fluoren-9-one (4.0 mmol, 1.6 g) and 20 wt% $Pd(OH)_2$ on carbon (0.32 g) were placed with a magnetic stir bar. The reaction vessel was equipped with H_2 balloon and then slightly vacuumed and refilled with H_2. The mixture was stirred under hydrogen at room temperature. After stirring for 20 h, the reaction mixture was refilled with N_2 and filtered through a pad of celite, and the solvent was removed under reduced pressure to give 2,7-dioctylfluorene (3.6 mmol, 1.4 g, 90%).

In a 100 mL round bottomed flask, 2,7-dioctylfluorene (3.6 mmol, 1.4 g) and KOH (7.9 mmol, 0.44 g) were placed with a magnetic stir bar. THF (10 mL) and DMF (10 mL) were subsequently added by a

syringe. The mixture was stirred under ambient conditions for 48 h. The resultant was diluted with H_2O, extracted three times with THF (20 mL x 3), then washed three times with brine (20 mL x 3). The combined organic layer was filtered through a short pad of Na_2SO_4/celite and concentrated in vacuo. The desired product 2,7-dioctyl-9*H*-fluoren-9-one (**2d**, 3.0 mmol, 1.2 g) was isolated by column chromatography on silica gel using hexane/ethyl acetate (1/0 to 20/1 to 10/1, v/v) as eluent.

2,7-Dioctyl-9*H*-fluoren-9-one (**2d**). yellow solid; m.p. 46.6-48.6 °C; ^1H NMR ($CDCl_3$, 400 MHz): δ 7.46 (d, J = 1.1 Hz, 2H), 7.36 (d, J = 7.6 Hz, 2H), 7.26-7.24 (m, 2H), 2.61 (t, J = 7.6 Hz, 4H), 1.65-1.58 (m, 4H), 1.31-1.26 (m, 20H), 0.88 (t, J = 6.7 Hz, 6H). $^{13}C\{^1H\}$ NMR ($CDCl_3$, 100 MHz): δ 194.8, 144.1, 142.4, 134.8, 134.7, 124.4, 120.0, 35.9, 32.0, 31.3, 29.6, 29.4, 29.3, 22.8, 14.3. HRMS (APCI) m/z $(M+H)^+$ calcd for $C_{29}H_{41}O$: 405.3152, found: 405.3174.

Detailed Optimization Studies

Table S1. Optimization studies for formal dehydrative coupling of biaryl **1a** and fluorenone **2a** for synthesis of spirobifluorene **3aa**[a]

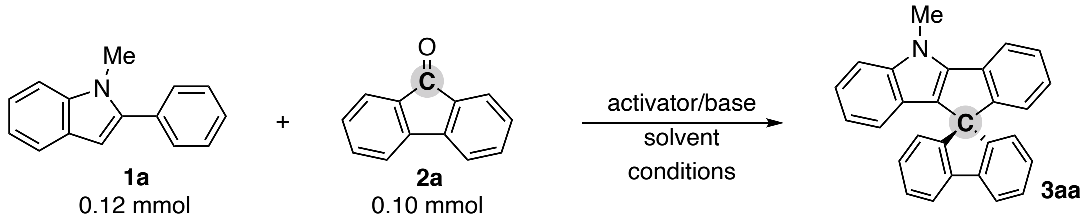

entry	activator (mmol)	base (mmol)	solvent	conditions	yield (%)[b]
1	Tf_2O (0.10)	none	DCE	120 ˚C, 16 h	56
2	***Tf_2O (0.12)***	***none***	***DCE***	***90 ˚C, 20 h***	***69 (72)***
3	Tf_2O (0.24)	none	DCE	90 ˚C, 20 h	40
4	Tf_2O (0.12)	2,6-lutidine (0.12)	DCE	90 ˚C, 20 h	40
5	Tf_2O (0.12)	**B1** (0.12)	DCE	90 ˚C, 20 h	(32)
6	Tf_2O (0.12)	**B2** (0.12)	DCE	90 ˚C, 20 h	(9)
7	Tf_2O (0.12)	DMAP (0.12)	DCE	90 ˚C, 20 h	55
8	Tf_2O (0.12)	Et_3N (0.12)	DCE	90 ˚C, 20 h	63
9	***Tf_2O (0.12)***	***Na_2CO_3 (0.12)***	***DCE***	***90 ˚C, 20 h***	***(90)***
10	Tf_2O (0.12)	K_2CO_3 (0.12)	DCE	90 ˚C, 20 h	72
11	Tf_2O (0.12)	Cs_2CO_3 (0.12)	DCE	90 ˚C, 20 h	61
12	Tf_2O (0.12)	KOAc (0.12)	DCE	90 ˚C, 20 h	38
13	Tf_2O (0.12)	K_3PO_4 (0.12)	DCE	90 ˚C, 20 h	31
14	***TfOH (0.24)***	***none***	***DCE***	***90 ˚C, 20 h***	***(90)***
15	TsOH•H_2O (0.24)	none	DCE	90 ˚C, 20 h	50
16	MsOH (0.24)	none	DCE	90 ˚C, 20 h	22
17	CF_3COOH (0.24)	none	DCE	90 ˚C, 20 h	68
18	TfOH (0.24)	Na_2CO_3 (0.12)	DCE	90 ˚C, 20 h	68
19	$PhNTf_2$ (0.12)	Na_2CO_3 (0.12)	DCE	90 ˚C, 20 h	0
20	Tf_2O (0.12)	Na_2CO_3 (0.12)	toluene	90 ˚C, 20 h	55
21	Tf_2O (0.12)	Na_2CO_3 (0.12)	$C_6H_5CF_3$	90 ˚C, 20 h	54
22	Tf_2O (0.12)	Na_2CO_3 (0.12)	1,4-dioxane	90 ˚C, 20 h	0
23	Tf_2O (0.12)	Na_2CO_3 (0.12)	DCE	60 ˚C, 20 h	30

24	Tf_2O (0.12)	Na_2CO_3 (0.12)	DCE	40 ˚C, 20 h	21
25	Tf_2O (0.12)	Na_2CO_3 (0.12)	DCE	RT, 20 h	13
26	Tf_2O (0.12)	Na_2CO_3 (0.12)	DCE	90 ˚C, 12 h	58
27	Tf_2O (0.12)	Na_2CO_3 (0.12)	DCE	90 ˚C, 4 h	63
28	none	none	DCE	90 ˚C, 20 h	0
29	none	Na_2CO_3 (0.12)	DCE	90 ˚C, 20 h	0

[a] Reaction conditions: activator, base, **1a** (0.12 mmol), **2a** (0.10 mmol), solvent (1.5 mL), N_2. [b] Estimated by GC method using dibenzyl as the internal standard. Isolated yields are shown in parentheses. Ms = methanesulfonyl, Ts = *p*-toluenesulfonyl.

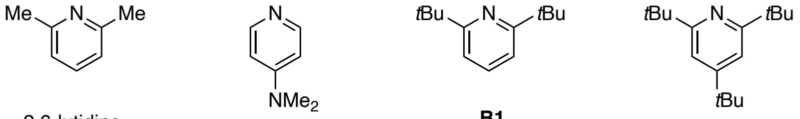

2,6-lutidine DMAP **B1** **B2**

X-Ray Analysis

The single X-ray quality crystals of **3da** were grown from hexane by slow evaporation at room temperature. The structure was refined by full-matrix least-squares method using SHELXL-2017/1.

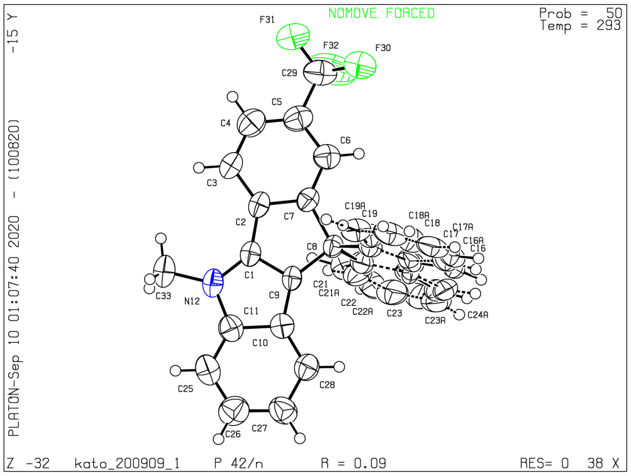

Figure S5. ORTEP drawing of **3da** (CCDC 2305237, 50% thermal probability).

Table S2. Crystal Data for **3da**

Crystal system	tetragonal
Space group IT number	86
Space group name H-M alt	P 42/n
Space group name Hall	-P 4bc
Cell length a	22.8051(3)
Cell length b	22.8051(3)
Cell length c	8.2792(2)
Cell angle alpha	90
Cell angle beta	90
Cell angle gamma	90
Cell volume	4305.78(15)
Cell formula units Z	8
Refine ls R factor all	0.0997
Refine ls R factor gt	0.0918
Refine ls wR factor gt	0.2877
Refine ls wR factor ref	0.2963
Refine ls goodness of fit ref	1.098

The single X-ray quality crystals of **3ha** were grown from THF/hexane by slow evaporation at room temperature. The structure was refined by full-matrix least-squares method using SHELXL-2017/1.

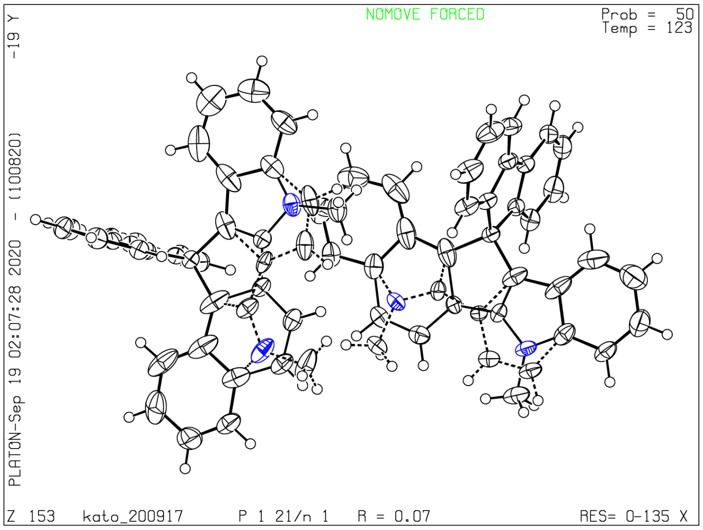

Figure S6. ORTEP drawing of **3ha** (CCDC 2305238, 50% thermal probability).

Table S3. Crystal Data for **3ha**

Crystal system	monoclinic
Space group IT number	14
Space group name H-M alt	P 1 21/n 1
Space group name Hall	-P 2yn
Cell length a	15.7190(3)
Cell length b	8.36430(10)
Cell length c	33.8437(5)
Cell angle alpha	90
Cell angle beta	101.487(2)
Cell angle gamma	90
Cell volume	4360.59(12)
Cell formula units Z	4
Refine ls R factor all	0.0752
Refine ls R factor gt	0.0691
Refine ls wR factor gt	0.1621
Refine ls wR factor ref	0.1659
Refine ls goodness of fit ref	1.134

The single X-ray quality crystals of **3ia’** were grown from $CHCl_3/CH_3CN$ by slow evaporation at room temperature. The structure was refined by full-matrix least-squares method using SHELXL-2017/1.

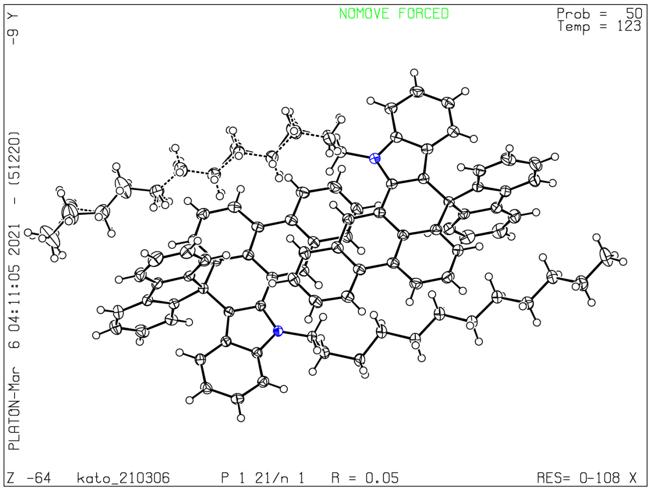

Figure S7. ORTEP drawing of **3ia’** (CCDC 2305239, 50% thermal probability).

Table S4. Crystal Data for **3ia’**

Crystal system	monoclinic
Space group IT number	14
Space group name H-M alt	P 1 21/n 1
Space group name Hall	-P 2yn
Cell length a	27.4355(3)
Cell length b	7.96430(10)
Cell length c	31.2422(3)
Cell angle alpha	90
Cell angle beta	91.9370(10)
Cell angle gamma	90
Cell volume	6814.10(13)
Cell formula units Z	4
Refine ls R factor all	0.0590
Refine ls R factor gt	0.0548
Refine ls wR factor gt	0.1401
Refine ls wR factor ref	0.1435
Refine ls goodness of fit ref	1.032

The single X-ray quality crystals of **3qa** were grown from CD_2Cl_2 by slow evaporation at room temperature. The structure was refined by full-matrix least-squares method using SHELXL-2017/1.

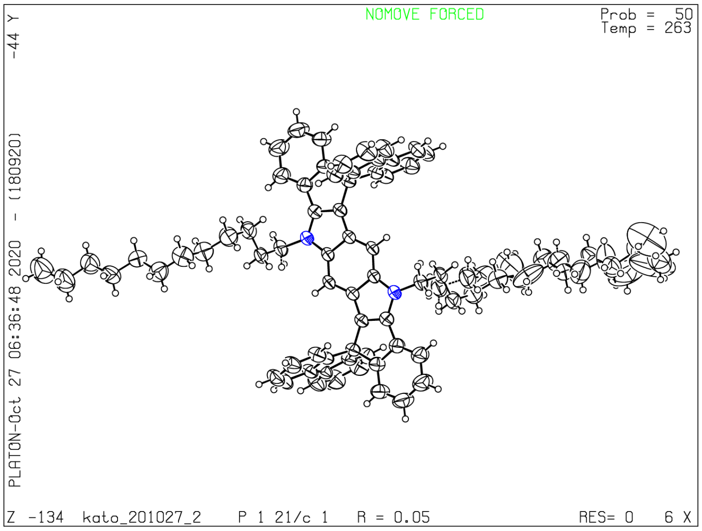

Figure S8. ORTEP drawing of **3qa** (CCDC 2305240, 50% thermal probability).

Table S5. Crystal Data for **3qa**

Crystal system	monoclinic
Space group IT number	14
Space group name H-M alt	P 1 21/c 1
Space group name Hall	-P 2ybc
Cell length a	15.0249(2)
Cell length b	11.65340(10)
Cell length c	16.3566(2)
Cell angle alpha	90
Cell angle beta	97.3120(10)
Cell angle gamma	90
Cell volume	2840.61(6)
Cell formula units Z	8
Refine ls R factor all	0.0581
Refine ls R factor gt	0.0530
Refine ls wR factor gt	0.1437
Refine ls wR factor ref	0.1500
Refine ls goodness of fit ref	1.061

The single X-ray quality crystals of **3ra** were grown from DCE/CH_3NO_2 by slow evaporation at room temperature. The structure was refined by full-matrix least-squares method using SHELXL-2017/1.

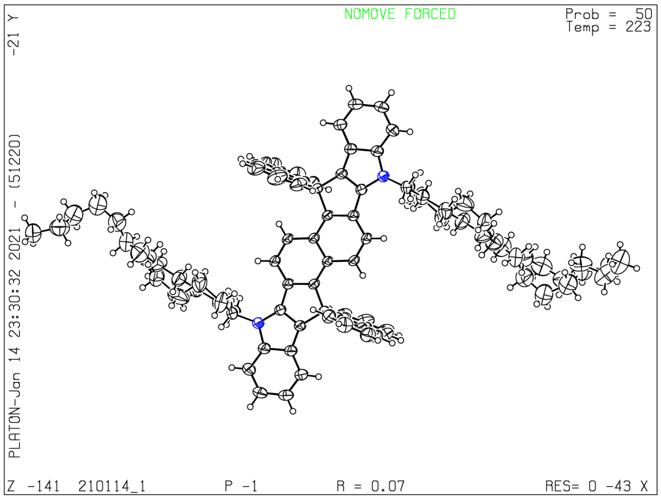

Figure S9. ORTEP drawing of **3ra** (CCDC 2305241, 50% thermal probability).

Table S6. Crystal Data for **3ra**

Crystal system	triclinic
Space group IT number	2
Space group name H-M alt	P -1
Space group name Hall	-P 1
Cell length a	10.9754(2)
Cell length b	12.1150(4)
Cell length c	12.3347(3)
Cell angle alpha	116.431(3)
Cell angle beta	95.048(2)
Cell angle gamma	94.470(2)
Cell volume	1450.31(7)
Cell formula units Z	2
Refine ls R factor all	0.0829
Refine ls R factor gt	0.0723
Refine ls wR factor gt	0.2040
Refine ls wR factor ref	0.2161
Refine ls goodness of fit ref	1.066

The single X-ray quality crystals of **3sa** were grown from DCE/CH_3CN by slow evaporation at room temperature. The structure was refined by full-matrix least-squares method using SHELXL-2017/1.

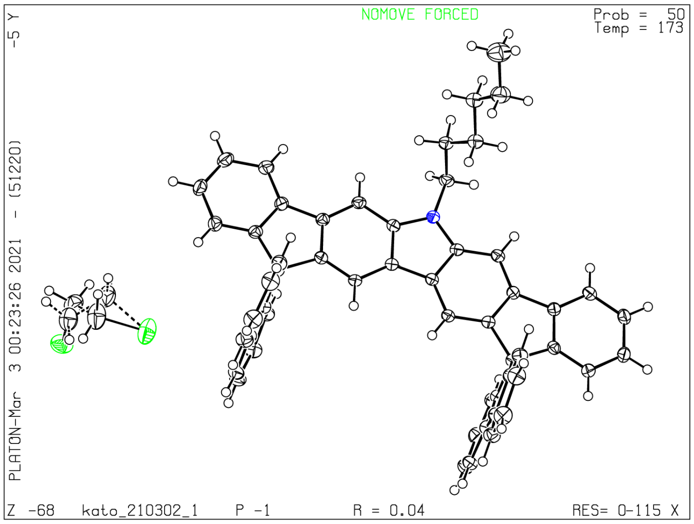

Figure S10. ORTEP drawing of **3sa** (CCDC 2305242, 50% thermal probability).

Table S7. Crystal Data for **3sa**

Crystal system	triclinic
Space group IT number	2
Space group name H-M alt	P -1
Space group name Hall	-P 1
Cell length a	10.38100(10)
Cell length b	14.3480(2)
Cell length c	15.61030(10)
Cell angle alpha	94.9290(10)
Cell angle beta	102.9010(10)
Cell angle gamma	107.0460(10)
Cell volume	2138.07(4)
Cell formula units Z	2
Refine ls R factor all	0.0439
Refine ls R factor gt	0.0396
Refine ls wR factor gt	0.1069
Refine ls wR factor ref	0.1104
Refine ls goodness of fit ref	1.069

The single X-ray quality crystals of **3ta** were grown from $CHCl_3$/hexane by slow evaporation at room temperature. The structure was refined by full-matrix least-squares method using SHELXL-2017/1.

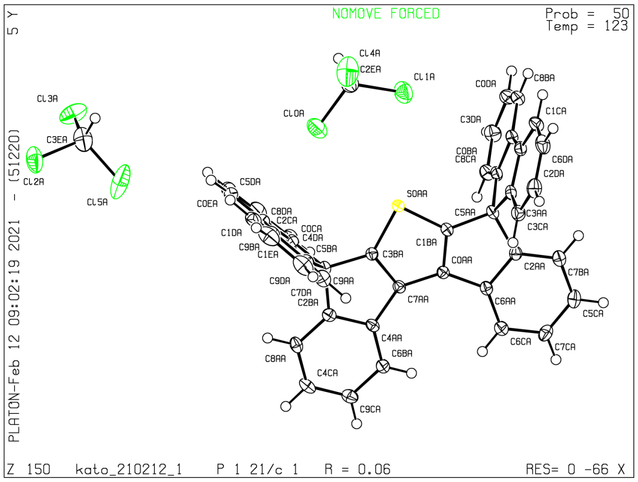

Figure S11. ORTEP drawing of **3ta** (CCDC 2305243, 50% thermal probability).

Table S8. Crystal Data for **3ta**

Crystal system	monoclinic
Space group IT number	14
Space group name H-M alt	P 1 21/c 1
Space group name Hall	-P 2ybc
Cell length a	12.2354(4)
Cell length b	14.2615(4)
Cell length c	20.8253(6)
Cell angle alpha	90
Cell angle beta	98.248(3)
Cell angle gamma	90
Cell volume	3596.33(19)
Cell formula units Z	4
Refine ls R factor all	0.0759
Refine ls R factor gt	0.0564
Refine ls wR factor gt	0.1565
Refine ls wR factor ref	0.1728
Refine ls goodness of fit ref	1.054

The single X-ray quality crystals of **3wd** were grown from DCE/CH_3NO_2 by slow evaporation at room temperature. The structure was refined by full-matrix least-squares method using SHELXL-2017/1.

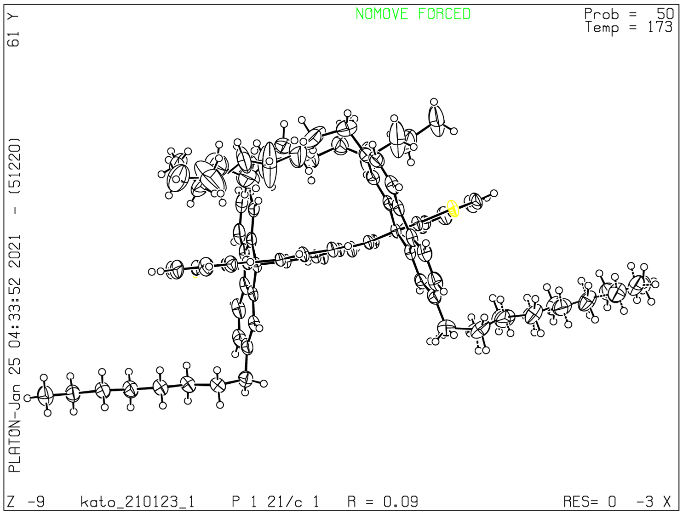

Figure S12. ORTEP drawing of **3wd** (CCDC 2305244, 50% thermal probability).

Table S9. Crystal Data for **3wd**

Crystal system	monoclinic
Space group IT number	14
Space group name H-M alt	P 1 21/c 1
Space group name Hall	-P 2ybc
Cell length a	22.0314(4)
Cell length b	12.4409(2)
Cell length c	26.6247(4)
Cell angle alpha	90
Cell angle beta	112.159(2)
Cell angle gamma	90
Cell volume	6758.6(2)
Cell formula units Z	4
Refine ls R factor all	0.1079
Refine ls R factor gt	0.0946
Refine ls wR factor gt	0.2904
Refine ls wR factor ref	0.3334
Refine ls goodness of fit ref	1.384

The single X-ray quality crystals of **3xa** were grown from $CHCl_3$/hexane by slow evaporation at room temperature. The structure was refined by full-matrix least-squares method using SHELXL-2017/1.

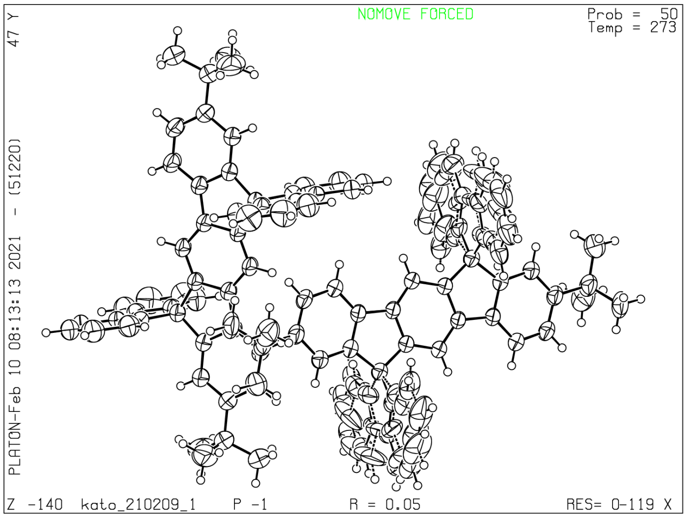

Figure S13. ORTEP drawing of **3xa** (CCDC 2305245, 50% thermal probability).

Table S10. Crystal Data for **3xa**

Crystal system	triclinic
Space group IT number	2
Space group name H-M alt	P -1
Space group name Hall	-P 1
Cell length a	12.0253(5)
Cell length b	14.2850(8)
Cell length c	15.2395(6)
Cell angle alpha	63.792(5)
Cell angle beta	67.462(4)
Cell angle gamma	77.789(4)
Cell volume	2166.5(2)
Cell formula units Z	2
Refine ls R factor all	0.0649
Refine ls R factor gt	0.0539
Refine ls wR factor gt	0.1612
Refine ls wR factor ref	0.1699
Refine ls goodness of fit ref	1.052

Tests for Possibility of Interconversion between 3la and 3la’

We tried the conversion of **3la** into **3la’** under Tf_2O/Na_2CO_3 conditions. However, the starting **3la** was recovered quantitatively. Similarly, no conversion of **3la’** was observed under Tf_2O/**B1** conditions. These results concluded no interconversion between **3la** and **3la’**.

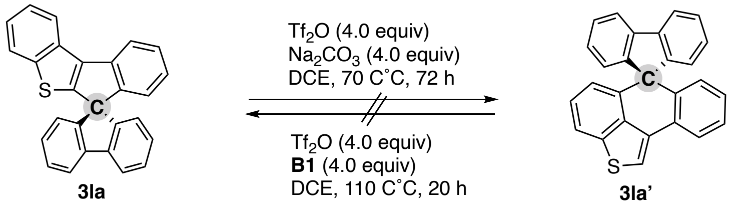

Table of Detailed Conditions for Each Substrate

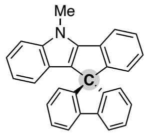

3aa 90%
1a (0.12 mmol)
2a (0.10 mmol)
TfOH (0.24 mmol)
DCE (1.5 mL), 90 ˚C, 20 h

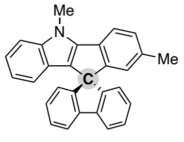

3ba 89%
1b (0.12 mmol)
2a (0.10 mmol)
TfOH (0.24 mmol)
DCE (1.5 mL), 90 ˚C, 20 h

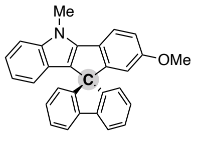

3ca 92%
1c (0.12 mmol)
2a (0.10 mmol)
TfOH (0.24 mmol)
DCE (1.5 mL), 90 ˚C, 20 h

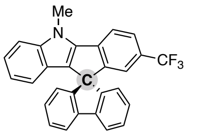

3da 69%
1d (0.12 mmol)
2a (0.10 mmol)
TfOH (0.24 mmol)
DCE (1.5 mL), 90 ˚C, 20 h

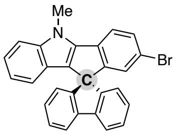

3ea 52%
1e (0.12 mmol)
2a (0.10 mmol)
Tf_2O/Na_2CO_3 (0.12 mmol)
DCE (1.5 mL), 90 ˚C, 20 h

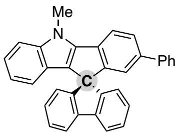

3fa 94%
1f (0.12 mmol)
2a (0.10 mmol)
TfOH (0.24 mmol)
DCE (1.5 mL), 90 ˚C, 20 h

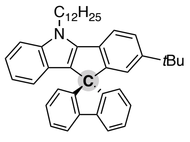

3ga 88%
1g (0.12 mmol)
2a (0.10 mmol)
TfOH (0.24 mmol)
DCE (1.5 mL), 90 ˚C, 20 h

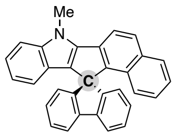

3ha 99%
1h (0.12 mmol)
2a (0.10 mmol)
TfOH (0.24 mmol)
DCE (1.5 mL), 90 ˚C, 20 h

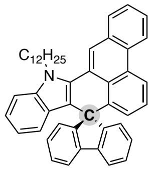

3ia' 84%
1i (0.060 mmol)
2a (0.050 mmol)
TfOH (0.12 mmol)
DCE (1.5 mL), 90 ˚C, 20 h

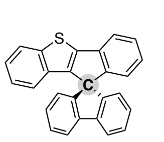

3ja 90%
1j (0.12 mmol)
2a (0.10 mmol)
Tf_2O/**B1** (0.12 mmol)
DCE (1.5 mL), 110 ˚C, 20 h

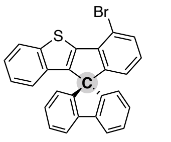

3ka 82%
1k (0.12 mmol)
2a (0.10 mmol)
Tf_2O/**B2** (0.12 mmol)
DCE (1.5 mL), 110 ˚C, 20 h

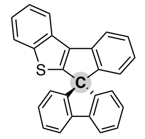

3la 44%
1l (0.12 mmol)
2a (0.10 mmol)
Tf_2O/$\mathbf{B_1}$ (0.12 mmol)
DCE (1.5 mL), 110 ˚C, 20 h

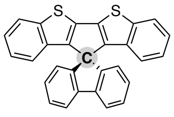

3ma 66%
1m (0.12 mmol)
2a (0.10 mmol)
Tf_2O/**B1** (0.12 mmol)
DCE (1.5 mL), 110 ˚C, 20 h

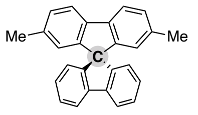

3na 48%
1n (0.40 mmol)
2a (0.10 mmol)
Tf_2O/Na_2CO_3 (0.12 mmol)
DCE (0.080 mL), 40 ˚C, 20 h

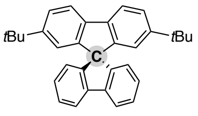

3oa 60%
1o (0.40 mmol)
2a (0.10 mmol)
Tf_2O/Na_2CO_3 (0.12 mmol)
DCE (0.080 mL), 40 ˚C, 20 h

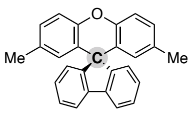

3pa 79%
1p (0.40 mmol)
2a (0.10 mmol)
Tf_2O/**B1** (0.12 mmol)
DCE (0.080 mL), 40 ˚C, 20 h

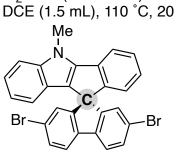

3ab 84%
1a (0.12 mmol)
2b (0.10 mmol)
TfOH (0.24 mmol)
DCE (1.5 mL), 90 ˚C, 20 h

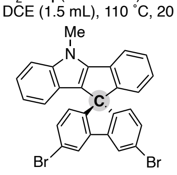

3ac 66%
1a (0.12 mmol)
2c (0.10 mmol)
TfOH (0.24 mmol)
DCE (1.5 mL), 90 ˚C, 20 h

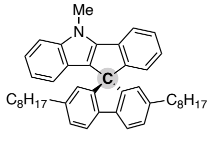

3ad 65%
1a (0.12 mmol)
2d (0.10 mmol)
TfOH (0.24 mmol)
DCE (1.5 mL), 90 ˚C, 20 h

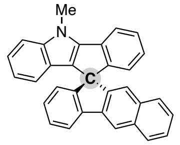

3ae 86%
1a (0.12 mmol)
2e (0.10 mmol)
TfOH (0.24 mmol)
DCE (1.5 mL), 90 ˚C, 20 h

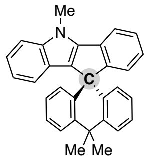

3af 76%
1a (0.12 mmol)
2f (0.10 mmol)
Tf_2O/Na_2CO_3 (0.12 mmol)
DCE (1.5 mL), 90 ˚C, 48 h

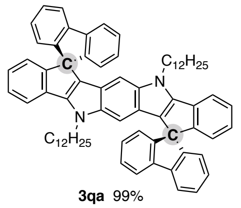

3qa 99%
1q (0.050 mmol)
2a (0.12 mmol)
TfOH (0.24 mmol)
DCE (1.5 mL), 90 ˚C, 20 h

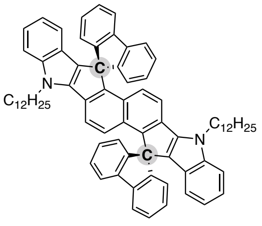

3ra 58%
1r (0.050 mmol)
2a (0.12 mmol)
TfOH (0.24 mmol)
DCE (1.5 mL), 90 ˚C, 20 h

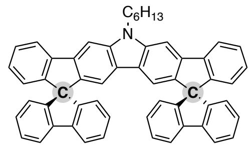

3sa 37%
1s (0.050 mmol)
2a (0.12 mmol)
Tf_2O/Na_2CO_3 (0.12 mmol)
DCE (0.080 mL), 40 ˚C, 20 h

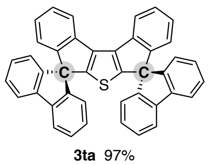

3ta 97%
1t (0.050 mmol)
2a (0.12 mmol)
Tf_2O/Na_2CO_3 (0.12 mmol)
DCE (0.080 mL), 40 ˚C, 20 h

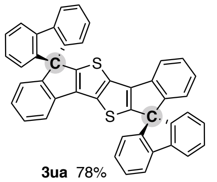

3ua 78%
1u (0.050 mmol)
2a (0.12 mmol)
TfOH (0.24 mmol)
DCE (1.5 mL), 110 ˚C, 20 h

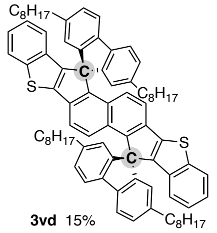

3vd 15%
1v (0.050 mmol)
2d (0.12 mmol)
Tf_2O/**B1** (0.12 mmol)
DCE (1.5 mL), 120 ˚C, 20 h

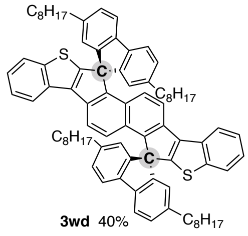

3wd 40%
1w (0.050 mmol)
2d (0.12 mmol)
Tf_2O/**B1** (0.12 mmol)
DCE (0.080 mL), 40 ˚C, 20 h

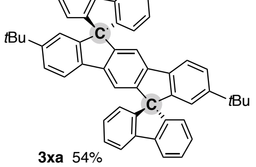

3xa 54%
1x (0.050 mmol)
2a (0.12 mmol)
Tf_2O/Na_2CO_3 (0.12 mmol)
DCE (0.080 mL), 40 ˚C, 20 h

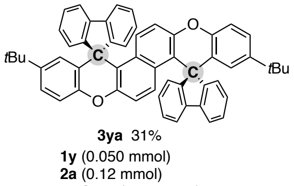

3ya 31%
1y (0.050 mmol)
2a (0.12 mmol)
Tf_2O/**B1** (0.12 mmol)
DCE (0.080 mL), 40 ˚C, 20 h

Photoluminescence Properties

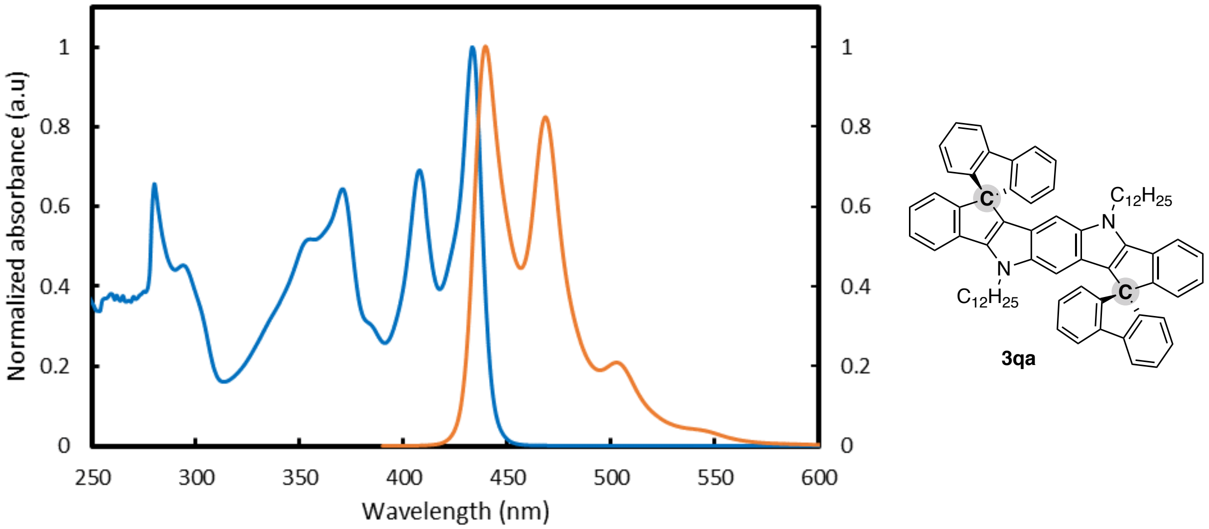

Figure S14. Normalized absorption (blue) and emission (orange) spectra of **3qa** (1.0×10^{-5} M in toluene). Excited at 370 nm for the emission spectrum.

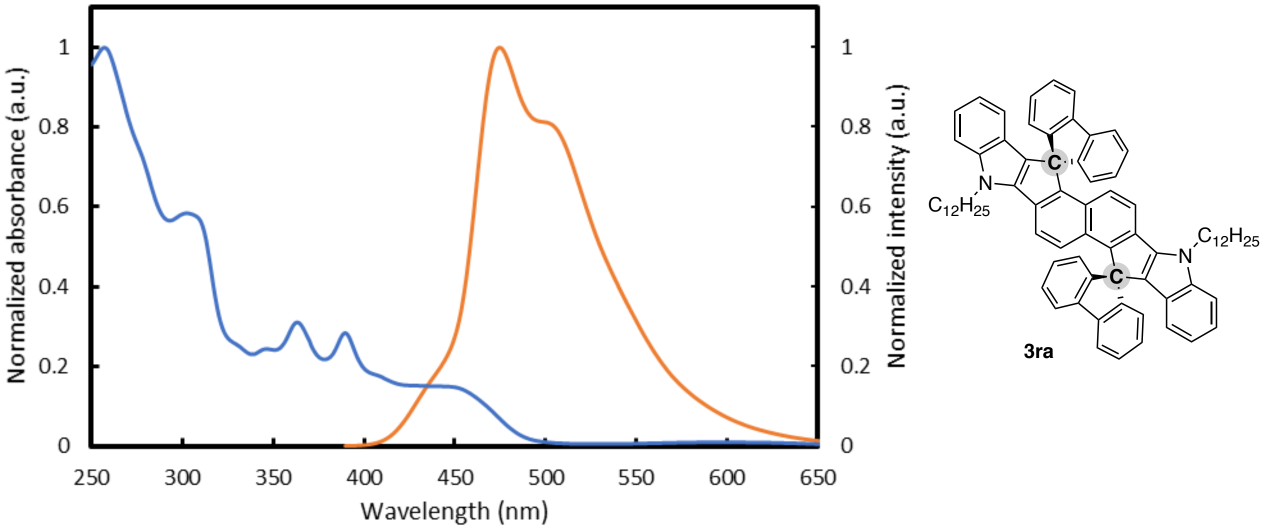

Figure S15. Normalized absorption (blue) and emission (orange) spectra of **3ra** (1.0×10^{-5} M in $CHCl_3$). Excited at 370 nm for the emission spectrum.

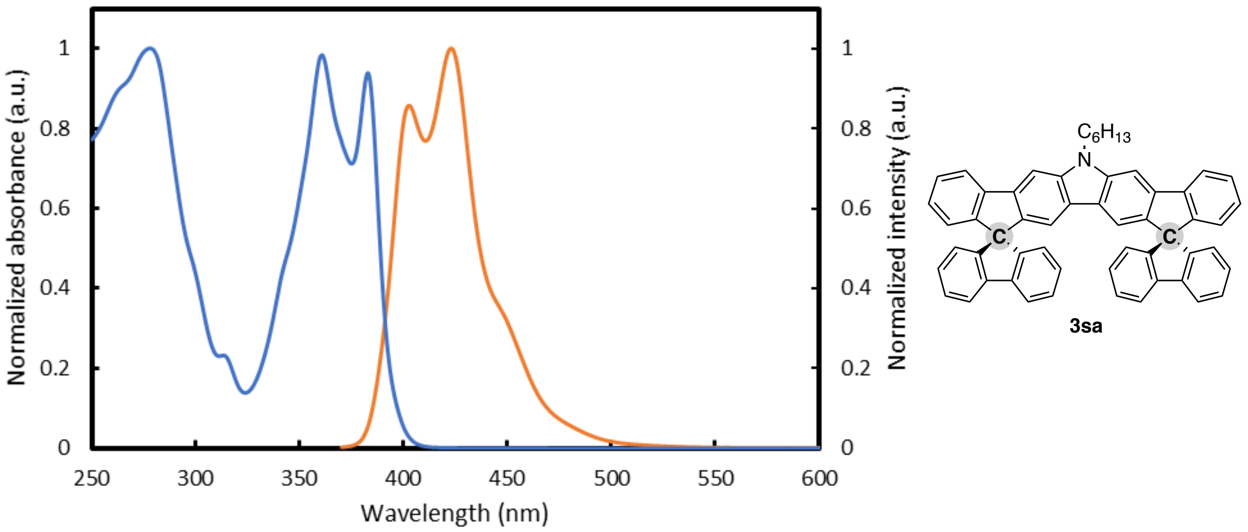

Figure S16. Normalized absorption (blue) and emission (orange) spectra of **3sa** ($1.0{\times}10^{-5}$ M in $CHCl_3$). Excited at 360 nm for the emission spectrum.

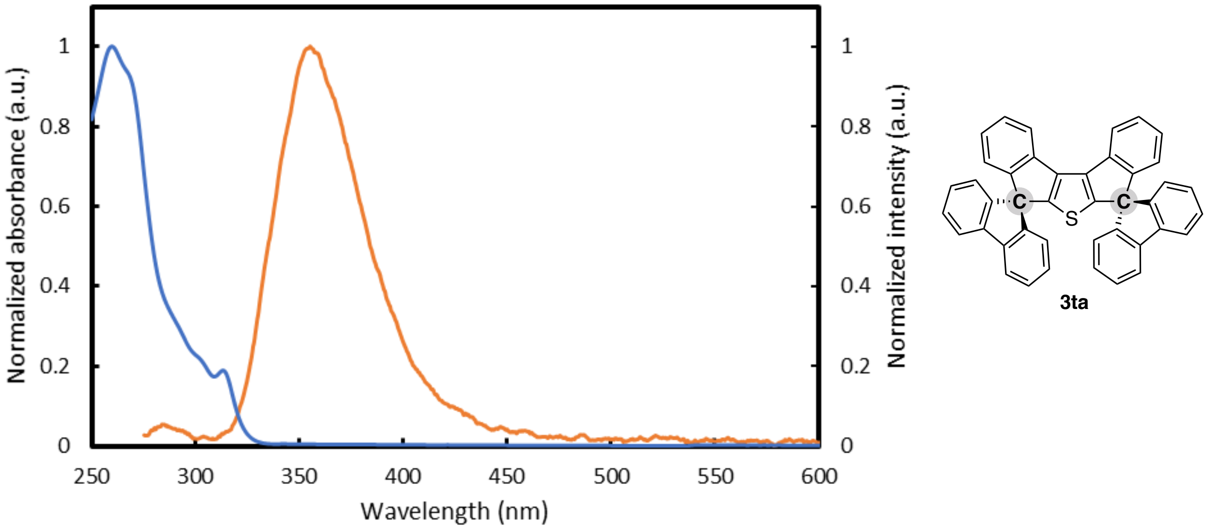

Figure S17. Normalized absorption (blue) and emission (orange) spectra of **3ta** ($1.0{\times}10^{-5}$ M in $CHCl_3$). Excited at 260 nm for the emission spectrum.

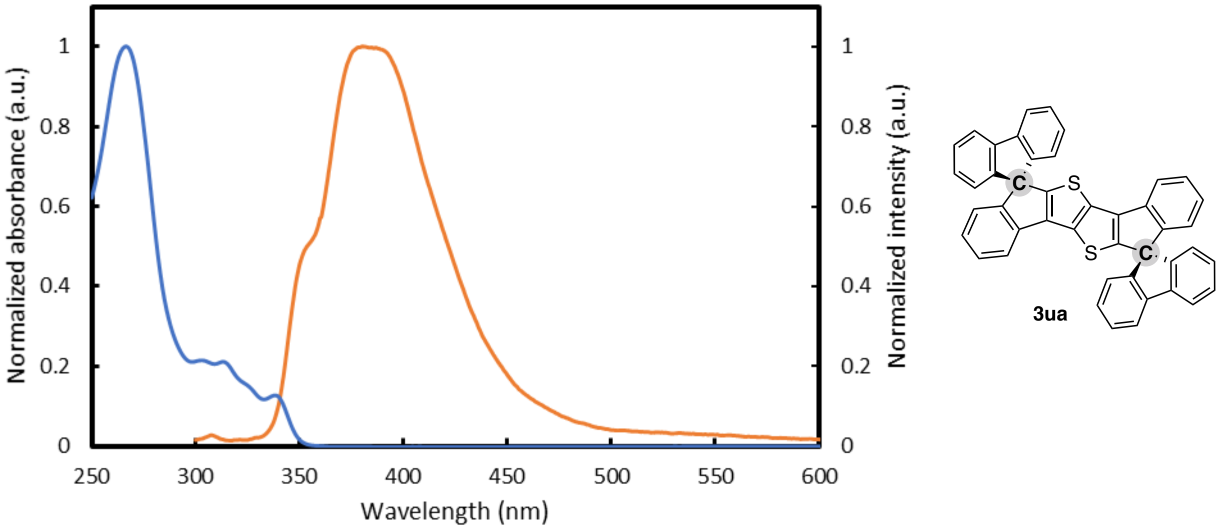

Figure S18. Normalized absorption (blue) and emission (orange) spectra of **3ua** (1.0×10^{-5} M in $CHCl_3$). Excited at 280 nm for the emission spectrum.

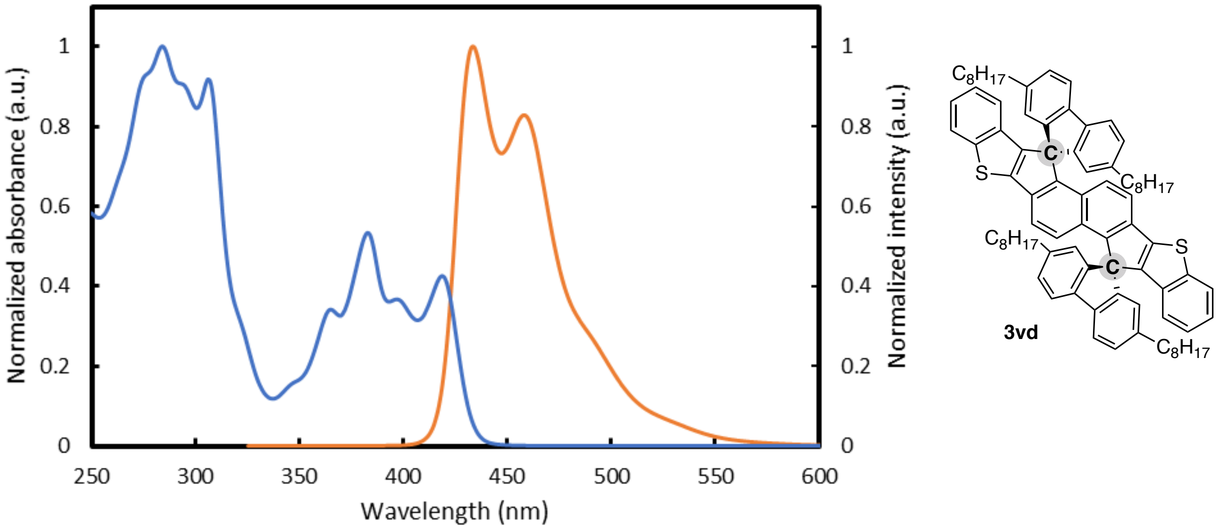

Figure S19. Normalized absorption (blue) and emission (orange) spectra of **3vd** (1.0×10^{-5} M in $CHCl_3$). Excited at 310 nm for the emission spectrum.

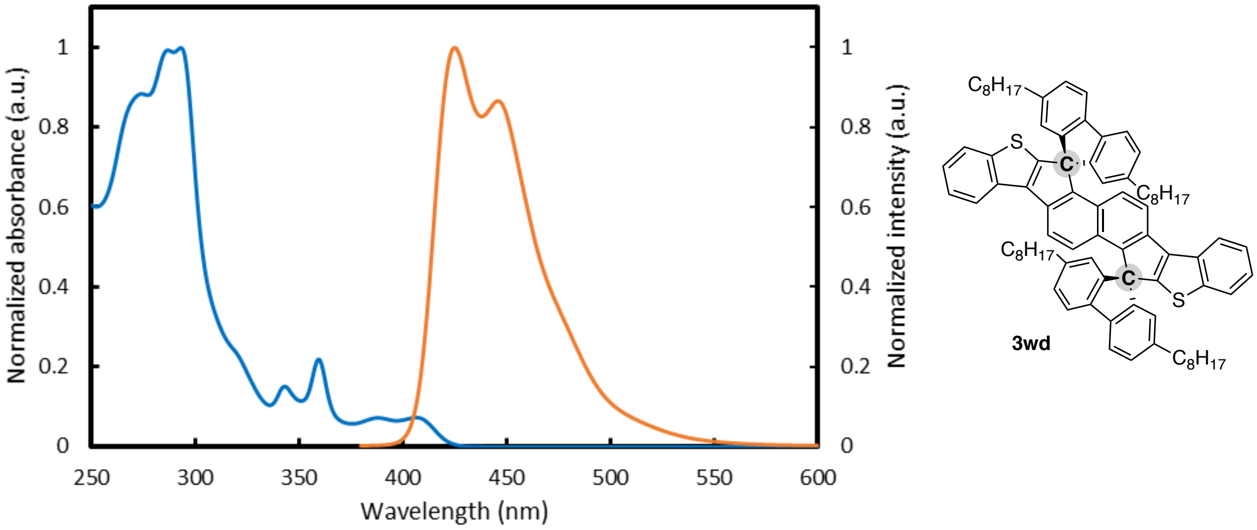

Figure S20. Normalized absorption (blue) and emission (orange) spectra of **3wd** (1.0×10^{-5} M in $CHCl_3$). Excited at 360 nm for the emission spectrum.

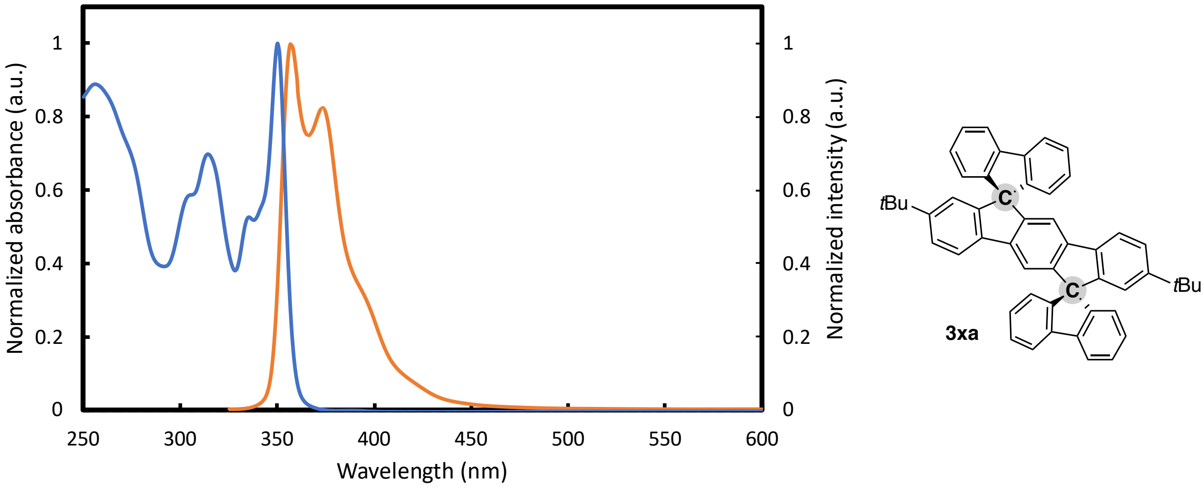

Figure S21. Normalized absorption (blue) and emission (orange) spectra of **3xa** (1.0×10^{-5} M in $CHCl_3$). Excited at 310 nm for the emission spectrum.

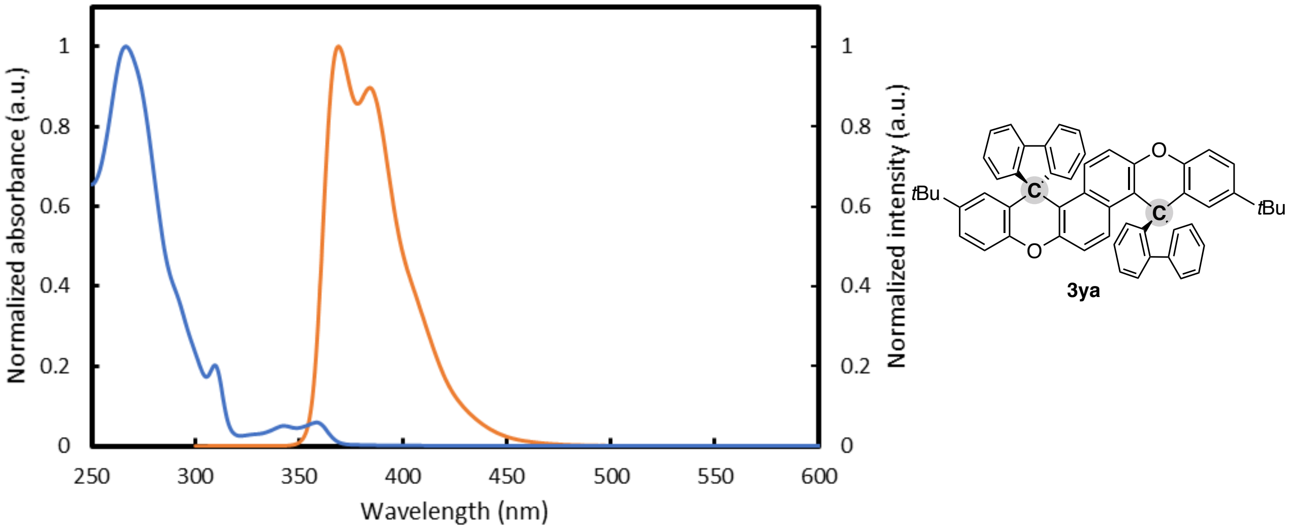

Figure S22. Normalized absorption (blue) and emission (orange) spectra of **3ya** (1.0×10^{-5} M in $CHCl_3$). Excited at 260 nm for the emission spectrum.

Table S11. Summary of optical properties.

compound	λ_{abs} (nm) (ε (10^4 M^{-1} cm^{-1}))	λ_{Fl} (nm)	Φ (%)
**3qa**	280 (3.1), 371 (3.0), 408 (3.2), 433 (4.7)	440, 469	83
**3ra**	303 (3.2), 363 (1.7), 390 (1.6), 447 (0.83)	475	39
**3sa**	278 (6.1), 361 (6.0), 383 (5.7)	403, 423	49
**3ta**	260 (4.8), 313 (0.90)	355	4
**3ua**	266 (7.5), 303 (1.6), 313 (1.6), 338 (0.96)	381	5
**3vd**	284 (8.0), 306 (7.4), 365 (2.7), 383 (4.3), 397 (2.9), 419 (3.4)	434, 458	48
**3wd**	287 (9.6), 293 (9.7), 343 (1.5), 359 (2.1), 388 (0.70), 406 (0.71)	425,446	20
**3xa**	305 (2.1), 314 (2.5), 335 (1.9), 350 (3.6)	357, 373	43
**3ya**	266 (7.3), 309 (1.5), 343 (0.37), 358 (0.43)	369, 384	19

Cyclic Voltammetry

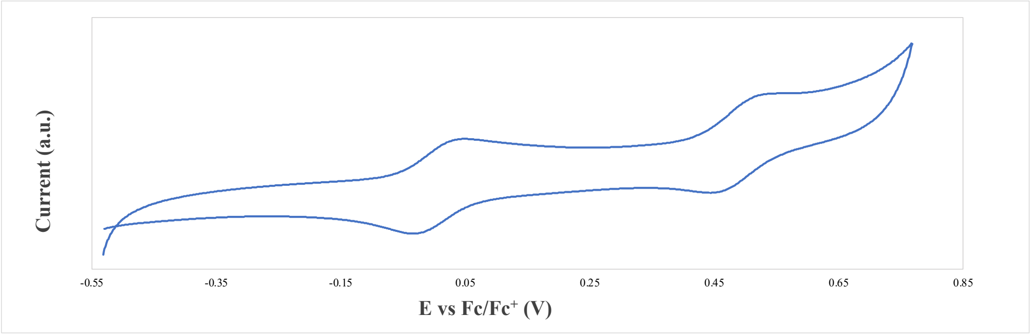

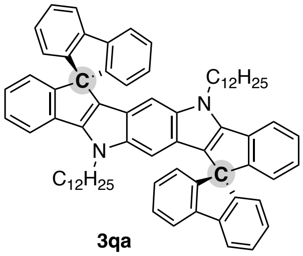

3qa

Figure S23. Cyclic voltammograms of **3qa** in o-dichlorobenzene/acetonitrile (10:1, v/v) containing 0.1 M n-Bu_4NPF_6 at a scan rate of 10.0 mVs^{-1}.

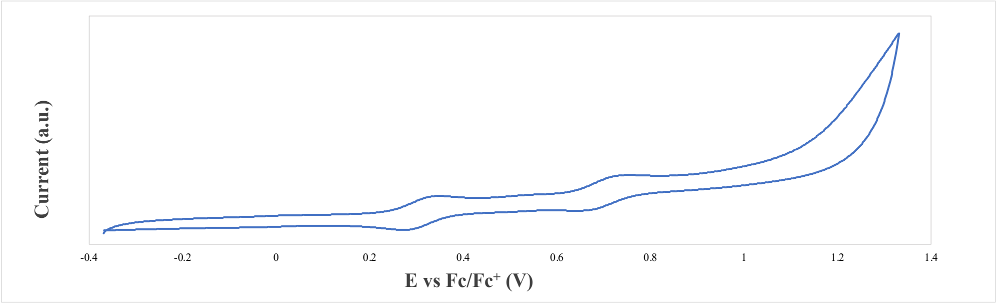

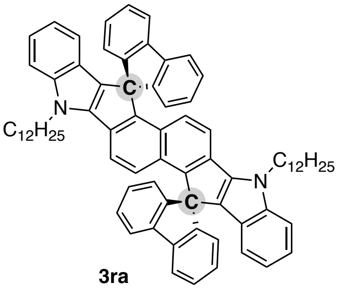

3ra

Figure S24. Cyclic voltammograms of **3ra** in o-dichlorobenzene/acetonitrile (10:1, v/v) containing 0.1 M n-Bu_4NPF_6 at a scan rate of 10.0 mVs^{-1}.

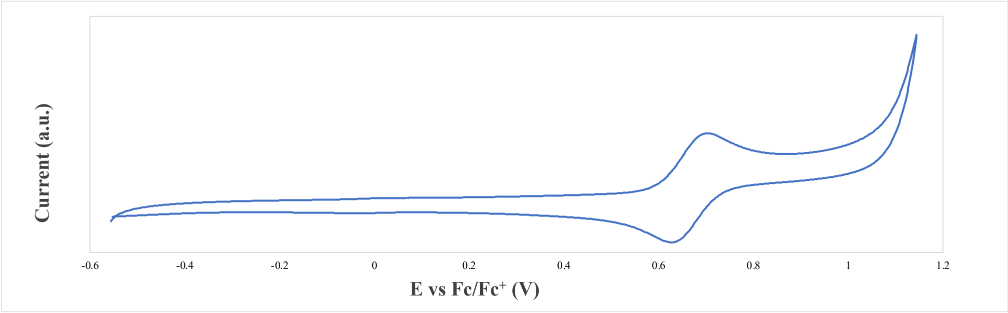

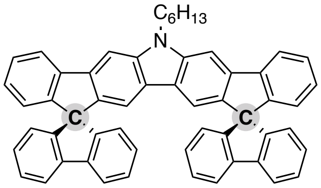

3sa

Figure S25. Cyclic voltammograms of **3sa** in o-dichlorobenzene/acetonitrile (10:1, v/v) containing 0.1 M n-Bu_4NPF_6 at a scan rate of 10.0 mVs^{-1}.

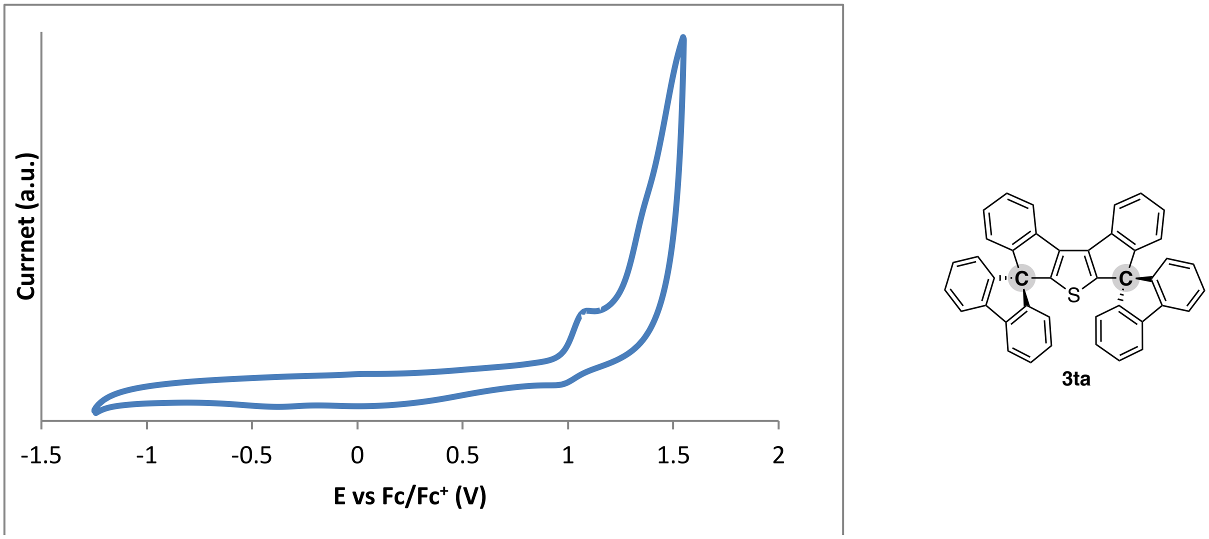

Figure S26. Cyclic voltammograms of **3ta** in o-dichlorobenzene/acetonitrile (10:1, v/v) containing 0.1 M n-Bu_4NPF_6 at a scan rate of 5.0 mVs^{-1}.

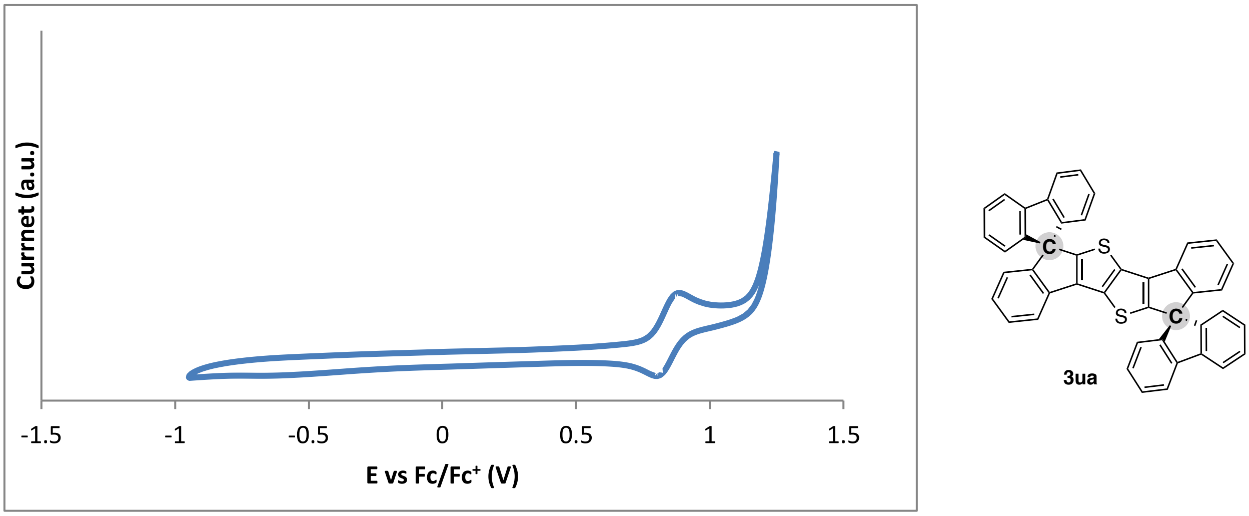

Figure S27. Cyclic voltammograms of **3ua** in o-dichlorobenzene/acetonitrile (10:1, v/v) containing 0.1 M n-Bu_4NPF_6 at a scan rate of 5.0 mVs^{-1}.

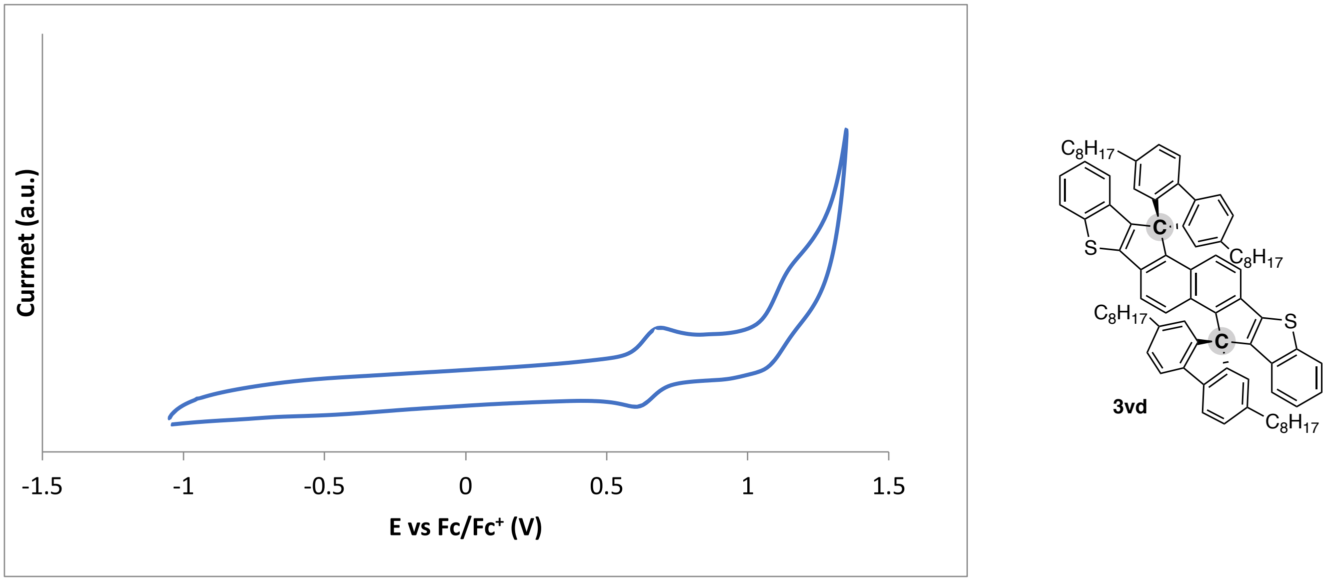

Figure S28. Cyclic voltammograms of **3vd** in o-dichlorobenzene/acetonitrile (10:1, v/v) containing 0.1 M n-Bu_4NPF_6 at a scan rate of 4.0 mVs^{-1}.

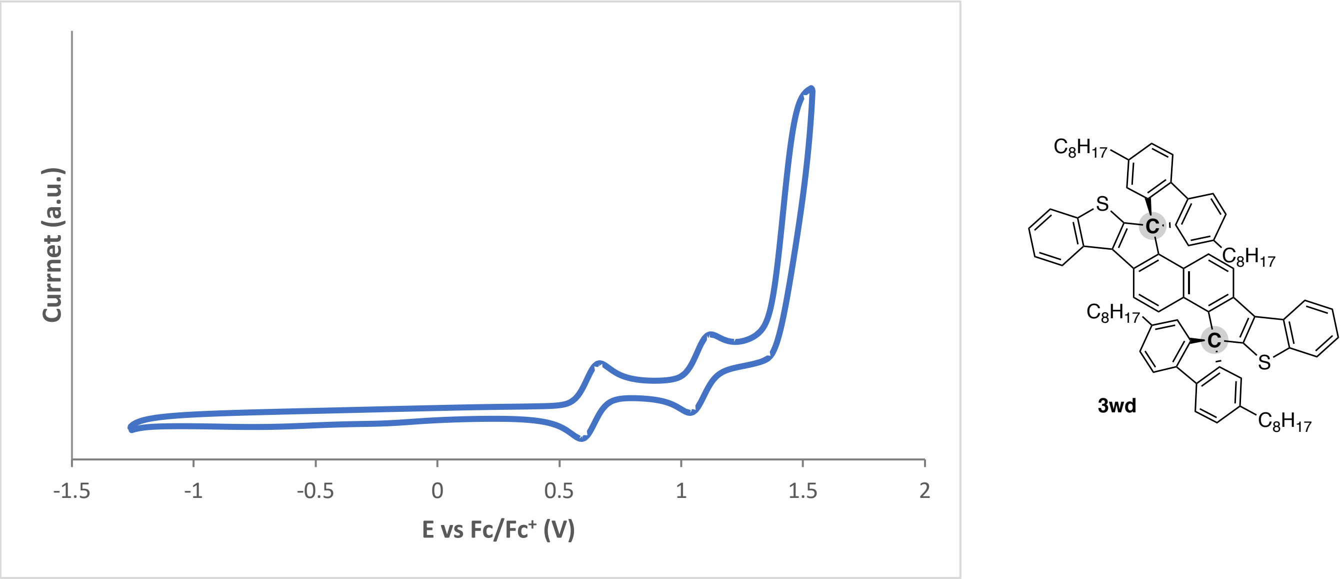

Figure S29. Cyclic voltammograms of **3wd** in o-dichlorobenzene/acetonitrile (10:1, v/v) containing 0.1 M n-Bu_4NPF_6 at a scan rate of 4.0 mVs^{-1}.

Table S12. Summary of absorption wavelengths, HOMO-LUMO energy gaps and cyclic voltammogram data.

**Compd.**	λ_{onset}^{abs} (nm)[a]	E_g^{opt} (eV)[b]	$E^{1/2}_{ox}$ (V)[c]	E_{HOMO} (eV)[d]	E_{LUMO} (eV)[e]
**3qa**	440	2.82	0.0085, 0.493	-4.81	-1.99
**3ra**	480	2.58	0.309, 0.697	-5.11	-2.53
**3sa**	401	3.09	0.665	-5.47	-2.38
**3ta**	328	3.78	1.05	-5.85	-2.07
**3ua**	348	3.56	0.843	-5.64	-2.08
**3vd**	432	2.87	0.647, 1.18[f]	-5.44	-2.58
**3wd**	420	2.95	0.628, 1.08	-5.43	-2.48
**3xa**	356	3.48	-	-	-
**3ya**	368	3.37	-	-	-

[a] Measured in toluene (**3qa**) and $CHCl_3$ (**3ra**-**3xa**). [b] Determined from the onset of the normalized absorption spectra. [c] Performed in *o*-dichlorobenzene/MeCN (10:1, v/v) in the presence of Bu_4NPF_6. v = 5.0 mV/s (**3ta**, **3ua**, **3vd**), v = 4.0 mV/s (**3wd**), versus Fc/Fc$^+$. [d] The approximation for Fc/Fc$^+$ level is -4.8 eV versus vacuum: $E_{HOMO} = -4.8 - E^{1/2}_{ox}$. [e] Estimated from E_{HOMO} and E_g^{opt}: $E_{LUMO} = E_{HOMO} + E_g^{opt}$. [f] E^p_{ox}

Characterization Data for Products

^{1}H, $^{13}C\{^{1}H\}$, and $^{19}F\{^{1}H\}$ NMR spectra for all compounds are attached in the last part.

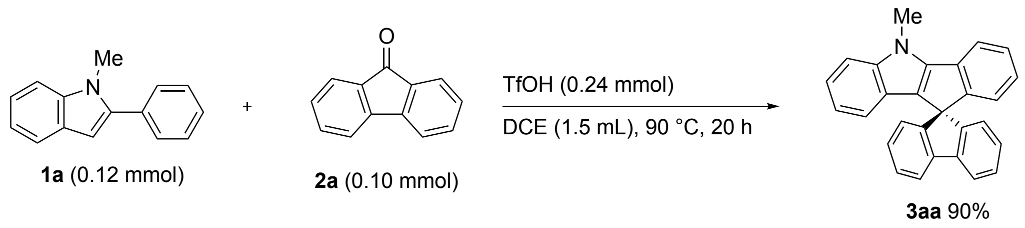

Synthesis of **3aa**: In a schlenk tube with pressure resistance, 1-methyl-2-phenyl-indole (**1a**, 0.12 mmol, 25 mg) and fluorenone (**2a**, 0.10 mmol, 18 mg) were placed with a magnetic stir bar. The reaction vessel was vacuumed and refilled with dry N_2. DCE (1.5 mL) was added by a syringe. The resulting mixture was stirred for 3 min. TfOH (0.24 mmol, 21 μL) was then added by a measuring pipette. The reaction mixture was heated at 90 ˚C in an oil bath for 20 h. After cooling, sat. $NaHCO_3$ aq was added. The resulting mixture was extracted three times with $CHCl_3$ (10 mL x 3). The combined organic layer was filtered through a short pad of celite/alumina and concentrated in vacuo. The desired product 5'-methyl-5'*H*-spiro[fluorene-9,10'-indeno[1,2-*b*]indole] (**3aa**) was isolated by column chromatography on silica gel using hexane/ethyl acetate (1/0 to 20/1, v/v) as eluent.

5'-Methyl-5'*H*-spiro[fluorene-9,10'-indeno[1,2-*b*]indole] (**3aa**). Purified by silica gel column chromatography with hexane/ethyl acetate (1/0 to 20/1, v/v): 34 mg (90%, 0.10 mmol scale); white solid; m.p. 237.7-238.7 °C; ^{1}H NMR ($CDCl_3$, 400 MHz): δ 7.86 (d, J = 7.6 Hz, 2H), 7.68 (d, J = 7.6 Hz, 1H), 7.35-7.32 (m, 3H), 7.28 (ddd, J = 7.5, 7.5, 0.9 Hz, 1H), 7.10 (ddd, J = 8.2, 8.2, 1.0 Hz, 1H), 7.05 (ddd, J = 7.5, 7.5, 0.9 Hz, 2H), 6.98 (ddd, J = 7.5, 7.5, 0.8 Hz, 1H), 6.82 (dd, J = 7.8, 7.8 Hz, 1H), 6.78 (d, J = 7.6 Hz, 2H), 6.67 (d, J = 7.6 Hz, 1H), 6.66 (d, J = 7.9 Hz, 1H), 4.14 (s, 3H). $^{13}C\{^{1}H\}$ NMR ($CDCl_3$, 100 MHz): δ 154.4, 147.5, 145.0, 142.3, 141.9, 135.7, 127.73, 127.71, 127.4, 126.1, 124.3, 124.2, 123.9, 122.6, 121.5, 120.1, 119.9, 118.8, 117.9, 109.8, 60.7, 31.5. HRMS (APCI) m/z $(M+H)^+$ calcd for $C_{28}H_{20}N$: 370.1590, found: 370.1587.

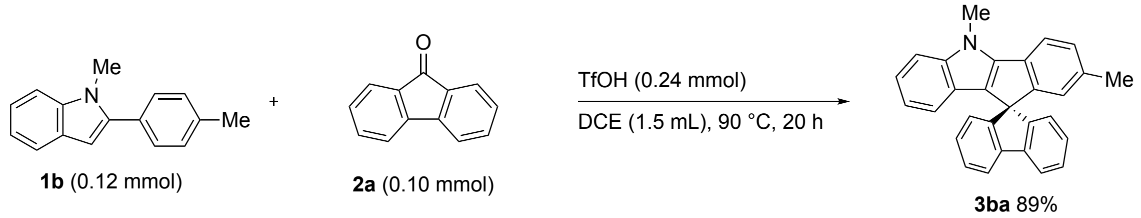

Synthesis of **3ba**: In a schlenk tube with pressure resistance, 1-methyl-2-phenyl-indole (**1b**, 0.12 mmol, 27 mg) and fluorenone (**2a**, 0.10 mmol, 18 mg) were placed with a magnetic stir bar. The reaction vessel was vacuumed and refilled with dry N_2. DCE (1.5 mL) was added by a syringe. The resulting mixture was stirred for 3 min. TfOH (0.24 mmol, 21 μL) was then added by a measuring pipette. The reaction mixture was heated at 90 ˚C in an oil bath for 20 h. After cooling, sat. $NaHCO_3$ aq was added. The resulting mixture was extracted three times with $CHCl_3$ (10 mL x 3). The combined organic layer was filtered through a short pad of celite/alumina and concentrated in vacuo. The residue was purified by column chromatography on silica gel using hexane/ethyl acetate (1/0 to 15/1, v/v). The desired product 2',5'-dimethyl-5'*H*-spiro[fluorene-9,10'-indeno[1,2-*b*]indole] (**3ba**) was obtained by addition of hexane, decantation, and drying.

2',5'-Dimethyl-5'*H*-spiro[fluorene-9,10'-indeno[1,2-*b*]indole] (**3ba**). Purified by silica gel column chromatography with hexane/ethyl acetate (1/0 to 15/1, v/v) as eluent followed by washing with hexane: 33 mg (89%, 0.10 mmol scale); white solid; m.p. 253.6-255.6 ºC; ^{1}H NMR ($CDCl_3$, 400 MHz): δ 7.87 (d, J = 7.6 Hz, 2H), 7.56 (d, J = 7.7 Hz, 1H), 7.34 (ddd, J = 7.5, 7.5, 1.1 Hz, 2H), 7.33 (d, J = 8.3 Hz, 1H), 7.10-7.05 (m, 2H), 7.305 (ddd, J = 7.5, 7.5, 1.0 Hz, 2H), 6.82-6.80 (m, 1H), 6.79 (d, J = 7.3 Hz, 2H), 6.64 (d, J = 7.9 Hz, 1H), 6.49 (s, 1H). 4.12 (s, 3H), 2.15 (s, 3H). ^{13}C{^{1}H} NMR ($CDCl_3$, 100 MHz): δ 154.6, 147.9, 145.3, 142.1, 141.9, 136.0, 133.0, 128.0, 127.7, 127.6, 125.2, 124.0, 123.4, 122.7, 121.2, 120.0, 119.8, 118.5, 117.6, 109.8, 60.6, 31.5, 21.5. HRMS (APCI) m/z $(M+H)^+$ calcd for $C_{29}H_{22}N$: 384.1747, found: 384.1737.

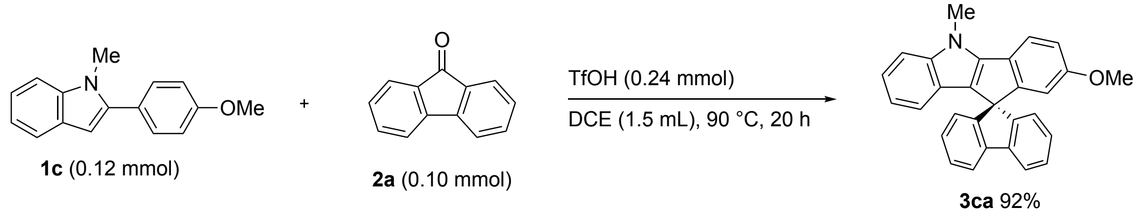

Synthesis of **3ca**: In a schlenk tube with pressure resistance, 2-(4-methoxyphenyl)-1-methyl-1*H*-indole

(**1c**, 0.12 mmol, 28 mg) and fluorenone (**2a**, 0.10 mmol, 18 mg) were placed with a magnetic stir bar. The reaction vessel was vacuumed and refilled with dry N_2. DCE (1.5 mL) was added by a syringe. The resulting mixture was stirred for 3 min. TfOH (0.24 mmol, 21 μL) was then added by a measuring pipette. The reaction mixture was heated at 90 ˚C in an oil bath for 20 h. After cooling, sat. $NaHCO_3$ aq was added. The resulting mixture was extracted three times with $CHCl_3$ (10 mL x 3). The combined organic layer was filtered through a short pad of celite/alumina and concentrated in vacuo. The desired product 2',5'-dimethyl-5'*H*-spiro[fluorene-9,10'-indeno[1,2-*b*]indole] (**3ca**) was isolated by column chromatography on silica gel using hexane/ethyl acetate (1/0 to 15/1 to 5/1, v/v) as eluent.

2'-Methoxy-5'-methyl-5'*H*-spiro[fluorene-9,10'-indeno[1,2-*b*]indole] (3ca). Purified by silica gel column chromatography with hexane/ethyl acetate (1/0 to 15/1 to 5/1, v/v): 37 mg (92%, 0.10 mmol scale); white solid; m.p. 223.3-225.3 °C; ^{1}H NMR ($CDCl_3$, 400 MHz): δ 7.86 (d, J = 7.6 Hz, 2H), 7.57 (d, J = 8.3 Hz, 1H), 7.34 (ddd, J = 7.5, 7.5, 1.0 Hz, 2H), 7.32 (d, J = 8.3 Hz, 1H), 7.08-7.04 (m, 3H), 6.83-6.78 (m, 4H), 6.62 (d, J = 7.8 Hz, 1H), 6.26 (d, J = 2.4 Hz, 1H), 4.11 (s, 3H), 3.62 (s, 3H). ^{13}C{^{1}H} NMR ($CDCl_3$, 100 MHz): δ 158.6, 156.6, 147.8, 145.1, 141.9, 141.8, 128.7, 127.74, 127.69, 124.0, 122.8, 122.6, 120.9, 120.0, 119.8, 118.3, 118.2, 112.1, 111.3, 109.7, 60.7, 55.6, 31.4. HRMS (APCI) m/z $(M+H)^+$ calcd for $C_{29}H_{22}NO$: 400.1696, found: 400.1704.

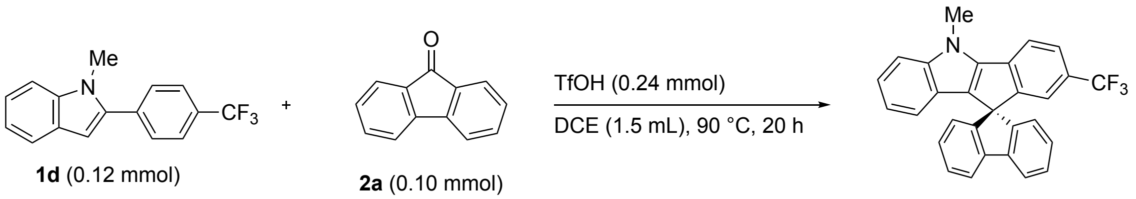

1d (0.12 mmol) **2a** (0.10 mmol) **3da** 69%

Synthesis of **3da**: In a schlenk tube with pressure resistance, 2-(4-methoxyphenyl)-1-methyl-1*H*-indole (**1d**, 0.12 mmol, 33 mg) and fluorenone (**2a**, 0.10 mmol, 18 mg) were placed with a magnetic stir bar. The reaction vessel was vacuumed and refilled with dry N_2. DCE (1.5 mL) was added by a syringe. The resulting mixture was stirred for 3 min. TfOH (0.24 mmol, 21 μL) was then added by a measuring pipette. The reaction mixture was heated at 90 ˚C in an oil bath for 20 h. After cooling, sat. $NaHCO_3$ aq was added. The resulting mixture was extracted three times with $CHCl_3$ (10 mL x 3). The combined organic layer was filtered through a short pad of celite/alumina and concentrated in vacuo. The residue was purified by column chromatography on silica gel using hexane/ethyl acetate (1/0 to 15/1 to 10/1, v/v) as eluent. The desired product 5'-methyl-2'-(trifluoromethyl)-5'*H*-spiro[fluorene-9,10'-indeno[1,2-*b*]indole]

(**3da**) was obtained by addition of hexane, decantation, and drying. The single crystals of **3da** suitable for X-ray analysis were grown from hexane.

5'-Methyl-2'-(trifluoromethyl)-5'*H*-spiro[fluorene-9,10'-indeno[1,2-*b*]indole] (**3da**). Purified by silica gel column chromatography with hexane/ethyl acetate (1/0 to 15/1 to 10/1, v/v) as eluent followed by washing with hexane: 30 mg (69%, 0.10 mmol scale); white solid; m.p. 260.6-262.6 °C; ^{1}H NMR ($CDCl_3$, 400 MHz): δ 7.89 (d, J = 7.6 Hz, 2H), 7.73 (d, J = 8.0 Hz, 1H), 7.57 (dd, J = 8.8, 0.6 Hz, 1H), 7.38 (d, J = 8.0 Hz, 1H), 7.36 (d, J = 8.0 Hz, 2H), 7.15 (dd, J = 8.2, 8.2 Hz, 1H), 7.07 (dd, J = 7.4, 7.4 Hz, 2H), 6.89 (s, 1H), 6.84 (dd, J = 7.1, 7.1 Hz, 1H), 6.76 (dd, J = 7.6, 0.6 Hz, 2H), 6.67 (dd, J = 8.0, 0.7 Hz, 1H), 4.15 (s, 3H). ^{13}C{^{1}H} NMR ($CDCl_3$, 100 MHz): δ 154.9, 146.2, 143.3, 142.7, 142.0, 139.1, 128.1, 127.9, 127.6 (q, J = 32 Hz), 126.8, 125.2 (q, J = 3.9 Hz), 124.4 (q, J = 270 Hz), 123.8, 122.6, 122.3, 121.0 (q, J = 3.6 Hz), 120.3, 120.2, 119.2, 117.5, 110.1, 60.7, 31.5. ^{19}F{^{1}H} NMR ($CDCl_3$, 376 MHz): δ -61.67.; HRMS (APCI) m/z $(M+H)^+$ calcd for $C_{29}H_{19}F_3N$: 438.1464, found: 438.1475.

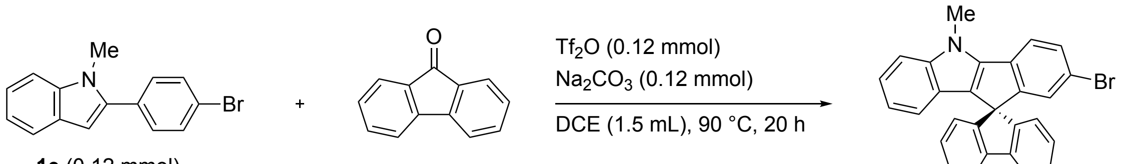

1e (0.12 mmol) **2a** (0.10 mmol) **3ea** 52%

Synthesis of **3ea**: In a schlenk tube with pressure resistance, 2-(4-bromophenyl)-1-methyl-1*H*-indole (**1e**, 0.12 mmol, 34 mg), fluorenone (**2a**, 0.10 mmol, 18 mg), and Na_2CO_3 (0.12 mmol, 13 mg) were placed with a magnetic stir bar. The reaction vessel was vacuumed and refilled with dry N_2. DCE (1.5 mL) was added by a syringe. The resulting mixture was stirred for 3 min. Tf_2O (0.12 mmol, 20 µL) was then added by a syringe. The reaction mixture was heated at 90 ˚C in an oil bath for 20 h. After cooling, sat. $NaHCO_3$ aq was added. The resulting mixture was extracted three times with $CHCl_3$ (10 mL x 3). The combined organic layer was filtered through a short pad of celite/alumina and concentrated in vacuo. The desired product 2'-bromo-5'-methyl-5'*H*-spiro[fluorene-9,10'-indeno[1,2-*b*]indole] (**3ea**) was isolated by column chromatography on silica gel using hexane/ethyl acetate (1/0 to 15/1, v/v) as eluent.

2'-Bromo-5'-methyl-5'*H*-spiro[fluorene-9,10'-indeno[1,2-*b*]indole] (**3ea**). Purified by silica gel column chromatography with hexane/ethyl acetate (1/0 to 15/1, v/v): 23 mg (52%, 0.10 mmol scale); white solid; m.p. 268.6-269.6 °C; ^{1}H NMR ($CDCl_3$, 400 MHz): δ 7.87 (d, J = 7.5 Hz, 2H), 7.53 (d, J =

8.1 Hz, 1H), 7.42 (dd, J = 8.1, 1.8 Hz, 1H), 7.37 (dd, J = 7.4, 7.4 Hz, 2H), 7.35 (d, J = 8.3 Hz, 1H), 7.12 (dd, J = 7.4, 7.4 Hz, 1H), 7.07 (dd, J = 7.5, 7.5 Hz, 2H), 6.82 (dd, J = 7.8, 7.8 Hz, 1H), 6.79-6.77 (m, 3H), 6.65 (d, J = 7.9 Hz, 1H), 4.13 (s, 3H). $^{13}C\{^1H\}$ NMR ($CDCl_3$, 100 MHz): δ 156.4, 146.6, 143.9, 142.4, 141.9, 134.6, 130.4, 128.0, 127.9, 127.6, 124.5, 123.9, 122.4, 122.0, 120.2, 120.1, 119.6, 118.9, 118.8, 109.9, 60.6, 31.5. HRMS (APCI) m/z $(M+H)^+$ calcd for $C_{28}H_{19}BrN$: 448.0695, found: 448.0679.

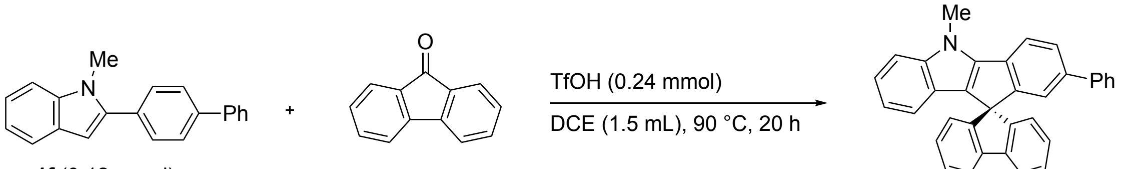

1f (0.12 mmol) **2a** (0.10 mmol) **3fa** 94%

Synthesis of **3af**: In a 20 mL screw cap test tube, 1-methyl-2-(4-(trifluoromethyl)phenyl)-1*H*-indole (**1f**, 0.12 mmol, 34 mg) and fluorenone (**2a**, 0.10 mmol, 18 mg) were placed with a magnetic stir bar. The reaction vessel was vacuumed and refilled with dry N_2. DCE (1.5 mL) was added by a syringe. The resulting mixture was stirred for 3 min. TfOH (0.24 mmol, 21 μL) was then added by a measuring pipette. The reaction mixture was heated at 90 ˚C in a heat block for 20 h. After cooling, sat. $NaHCO_3$ aq was added. The resulting mixture was extracted three times with $CHCl_3$ (10 mL x 3). The combined organic layer was filtered through a short pad of celite/alumina and concentrated in vacuo. The desired product 5'-methyl-2'-phenyl-5'*H*-spiro[fluorene-9,10'-indeno[1,2-*b*]indole] (**3fa**) was isolated by column chromatography on silica gel using hexane/ethyl acetate (1/0 to 10/1, v/v) as eluent.

5'-Methyl-2'-phenyl-5'*H*-spiro[fluorene-9,10'-indeno[1,2-*b*]indole] (3fa). Purified by silica gel column chromatography with hexane/ethyl acetate (1/0 to 10/1, v/v): 42 mg (94%, 0.10 mmol scale); white solid; m.p. 226.6-228.6 °C; ^1H NMR ($CDCl_3$, 400 MHz): δ 7.87 (d, J = 7.6 Hz, 2H), 7.72 (d, J = 7.9 Hz, 1H), 7.54 (dd, J = 7.9, 1.5 Hz, 1H), 7.39-7.32 (m, 5H), 7.27 (dd, J = 7.8, 7.8 Hz, 2H), 7.21-7,18 (m, 1H), 7.10 (dd, J = 8.1, 8.1 Hz, 1H), 7.05 (dd, J = 7.4, 7.4 Hz, 2H), 6.90 (s, 1H), 6.83 (d, J = 7.4 Hz, 2H), 6.82 (dd, J = 7.7, 7.7 Hz, 1H), 6.67 (dd, J = 7.9, 0.4 Hz, 1H), 4.13 (s, 3H). $^{13}C\{^1H\}$ NMR ($CDCl_3$, 100 MHz): δ 155.2, 147.5, 144.7, 142.4, 141.9, 140.9, 139.0, 134.8, 128.7, 127.78, 127.75, 127.2, 127.0, 126.4, 124.7, 124.0, 123.1, 122.6, 121.6, 120.1, 119.9, 118.8, 118.0, 109.9, 60.8, 31.5. HRMS (APCI) m/z $(M+H)^+$ calcd for $C_{34}H_{24}N$: 446.1903, found: 446.1911.

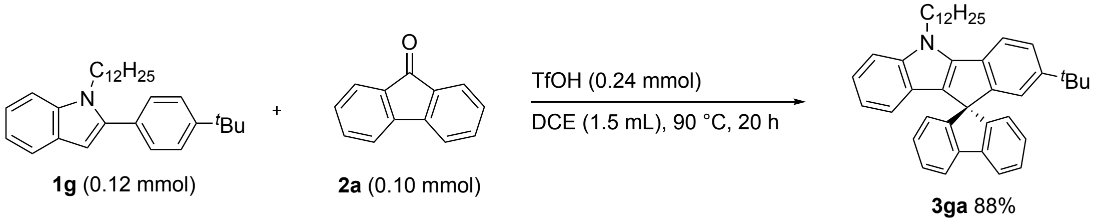

Synthesis of **3ga**: In a 20 mL screw cap test tube, 2-(4-(*tert*-butyl)phenyl)-1-dodecyl-1*H*-indole (**1g**, 0.12 mmol, 50 mg) fluorenone (**2a**, 0.10 mmol, 18 mg) were placed with a magnetic stir bar. The reaction vessel was vacuumed and refilled with dry N_2. DCE (1.5 mL) was added by a syringe. The resulting mixture was stirred for 3 min. TfOH (0.24 mmol, 21 μL) was then added by a measuring pipette. The reaction mixture was heated at 90 ˚C in a heat block for 20 h. After cooling, sat. $NaHCO_3$ aq was added. The resulting mixture was extracted three times with $CHCl_3$ (10 mL x 3). The combined organic layer was filtered through a short pad of celite/alumina and concentrated in vacuo. The desired product 2'-(*tert*-butyl)-5'-dodecyl-5'*H*-spiro[fluorene-9,10'-indeno[1,2-*b*]indole] (**3ga**) was isolated by column chromatography on silica gel using hexane/ethyl acetate (1/0 to 10/1, v/v) as eluent followed by GPC (chloroform).

2'-(*tert*-Butyl)-5'-dodecyl-5'*H*-spiro[fluorene-9,10'-indeno[1,2-*b*]indole] (**3ga**). Purified by silica gel column chromatography with hexane/ethyl acetate (1/0 to 10/1, v/v) as eluent followed by GPC (chloroform): 51 mg (88%, 0.10 mmol scale); white solid; m.p. 61.2-63.2 ^{o}C; ^{1}H NMR ($CDCl_3$, 400 MHz): δ 7.87 (d, J = 7.6 Hz, 2H), 7.53 (d, J = 8.0 Hz, 1H), 7.35-7.32 (m, 4H), 7.07-7.03 (m, 3H), 6.80-6.77 (m, 3H), 6.700-6.695 (m, 1H), 6.60 (d, J = 7.9 Hz, 1H), 4.48 (t, J = 7.4 Hz, 2H), 2.06-1.99 (m, 2H), 1.54-1.47 (m, 2H), 1.41-1.38 (m, 2H), 1.32-1.26 (m, 14H), 1.14 (s, 9H), 0.88 (t, J = 6.5 Hz, 3H). ^{13}C{^{1}H} NMR ($CDCl_3$, 100 MHz): δ 154.4, 149.4, 148.0, 144.5, 141.9, 141.5, 133.2, 127.7, 127.5, 124.2 (2C), 124.1, 122.7, 121.5, 121.1, 119.9, 119.6, 118.6, 117.3, 110.0, 60.9, 45.2, 34.9, 32.1, 31.5, 30.9, 29.79, 29.78, 29.76, 29.70, 29.59, 29.50, 27.3, 22.8, 14.3. HRMS (APCI) m/z $(M+H)^+$ calcd for $C_{43}H_{50}N$: 580.3938, found: 580.3957.

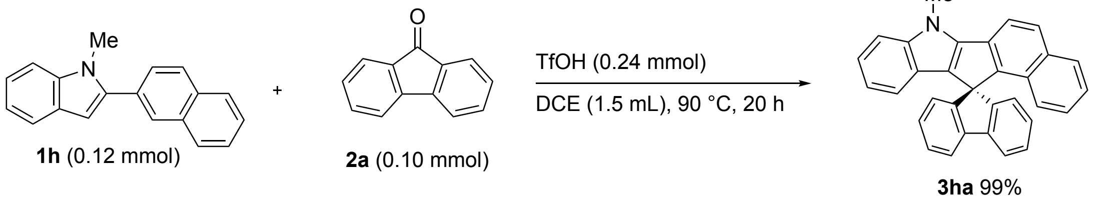

Synthesis of **3ha**: In a schlenk tube with pressure resistance, 1-methyl-2-(naphthalen-2-yl)-1*H*-indole (**1h**, 0.12 mmol, 31 mg) and fluorenone (**2a**, 0.10 mmol, 18 mg) were placed with a magnetic stir bar. The reaction vessel was vacuumed and refilled with dry N_2. DCE (1.5 mL) was added by a syringe. The resulting mixture was stirred for 3 min. TfOH (0.24 mmol, 21 µL) was then added by a measuring pipette. The reaction mixture was heated at 90 ˚C in an oil bath for 20 h. After cooling, sat. $NaHCO_3$ aq was added. The resulting mixture was extracted three times with $CHCl_3$ (10 mL x 3). The combined organic layer was filtered through a short pad of celite/alumina and concentrated in vacuo. The desired product 7-methyl-7*H*-spiro[benzo[4,5]indeno[1,2-*b*]indole-12,9'-fluorene] (**3ha**) was isolated by column chromatography on silica gel using hexane/ethyl acetate (1/0 to 10/1, v/v) as eluent. The single crystals of **3ha** suitable for X-ray analysis were grown from THF/hexane.

7-Methyl-7*H*-spiro[benzo[4,5]indeno[1,2-*b*]indole-12,9'-fluorene] (**3ha**). Purified by silica gel column chromatography with hexane/ethyl acetate (1/0 to 10/1, v/v): 42 mg (99%, 0.10 mmol scale); yellow solid; m.p. >300.0 ºC; ^{1}H NMR ($CDCl_3$, 400 MHz): δ 8.00-7.96 (m, 3H), 7.92 (d, J = 8.4 Hz, 1H), 7.78 (d, J = 8.2 Hz, 1H), 7.36 (ddd, J = 7.5, 7.5, 1.0 Hz, 2H), 7.33 (d, J = 8.4 Hz, 1H), 7.19 (ddd, J = 8.1, 6.9, 1.1 Hz, 1H), 7.07 (ddd, J = 8.3, 7.1, 1.2 Hz, 1H), 7.00 (ddd, J = 7.5, 7.5, 1.1 Hz, 2H), 6.98 (ddd, J = 8.0, 8.0, 1.2 Hz, 1H), 6.79 (ddd, J = 7.9, 7.0, 0.8 Hz, 1H), 6.73 (dd, J = 8.7, 0.7 Hz, 1H), 6.71 (d, J = 7.6 Hz, 2H), 6.61 (d, J = 7.8 Hz, 1H), 4.21 (s, 3H). ^{13}C{^{1}H} NMR ($CDCl_3$, 100 MHz): δ 147.9, 147.2, 144.4, 142.0, 141.7, 134.4, 132.5, 130.0, 129.4, 128.9, 127.9, 127.7, 126.9, 126.0, 124.8, 124.0, 122.9, 122.3, 121.3, 120.5, 119.9, 118.1, 117.1, 109.8, 61.3, 31.5. HRMS (APCI) m/z $(M+H)^+$ calcd for $C_{32}H_{22}N$: 420.1747, found: 420.1733.

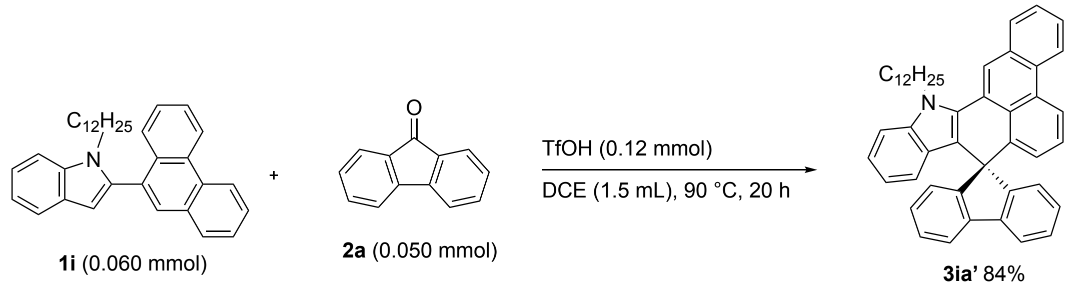

Synthesis of **3ia'**: In a 20 mL screw cap test tube, 1-dodecyl-2-(phenanthren-9-yl)-1*H*-indole (**1i**, 0.060 mmol, 28 mg) and fluorenone (**2a**, 0.050 mmol, 9 mg) were placed with a magnetic stir bar. The reaction vessel was vacuumed and refilled with dry N_2. DCE (1.5 mL) was added by a syringe. The resulting mixture was stirred for 3 min. TfOH (0.12 mmol, 11 µL) was then added by a measuring pipette. The

reaction mixture was heated at 90 ˚C in a heat block for 20 h. After cooling, sat. $NaHCO_3$ aq was added. The resulting mixture was extracted three times with $CHCl_3$ (10 mL x 3). The combined organic layer was filtered through a short pad of celite/alumina and concentrated in vacuo. The desired product 13'-dodecyl-13'*H*-spiro[fluorene-9,8'-phenanthro[10,1-*ab*]carbazole] (**3ia'**) was isolated by column chromatography on silica gel using hexane/ethyl acetate (1/0 to 20/1, v/v) as eluent followed by GPC (chloroform). The single crystals of **3ia'** suitable for X-ray analysis were grown from $CHCl_3/CH_3CN$.

13'-Dodecyl-13'*H*-spiro[fluorene-9,8'-phenanthro[10,1-*ab*]carbazole] (**3ia'**). Purified by silica gel column chromatography with hexane/ethyl acetate (1/0 to 20/1, v/v) as eluent followed by GPC (chloroform): 26 mg (84%, 0.050 mmol scale); yellow solid; m.p. 124.6-126.6 ºC; ^{1}H NMR ($CDCl_3$, 400 MHz): δ 8.61-8.59 (m, 1H), 8.52 (d, J = 7.9 Hz, 1H), 8.21 (s, 1H), 7.91 (d, J = 8.8 Hz, 1H), 7.90 (d, J = 7.6 Hz, 2H), 7.63-7.57 (m, 2H), 7.34 (dd, J = 7.6, 7.6 Hz, 2H), 7.33 (d, J = 8.2 Hz, 1H), 7.30 (d, J = 8.3 Hz, 1H), 7.06 (dd, J = 7.5, 7.5 Hz, 2H), 7.06 (d, J = 7.4 Hz, 1H), 6.98 (d, J = 7.6 Hz, 2H), 6.81 (d, J = 7.5 Hz, 1H), 6.64 (dd, J = 7.9, 7.9 Hz, 1H), 6.25 (d, J = 8.0 Hz, 1H), 4.64 (t, J = 8.0 Hz, 2H), 2.21-2.13 (m, 2H), 1.64-1.57 (m, 2H), 1.54-1.47 (m, 2H), 1.37-1.27 (m, 14H), 0.88 (t, J = 5.6 Hz, 3H). ^{13}C{^{1}H} NMR ($CDCl_3$, 100 MHz): δ 155.1, 140.1, 140.0, 139.5, 132.4, 131.9, 131.0, 130.1, 128.8, 128.3, 127.7, 127.6, 127.2, 127.0, 126.7, 126.6, 125.6, 124.5, 124.4, 122.9, 122.8, 121.4, 120.0, 119.9, 119.6, 119.5, 116.1, 109.3, 57.3, 46.2, 32.0, 30.5, 29.72 (3C), 29.67, 29.5, 29.4, 27.2, 22.8, 14.2. HRMS (APCI) m/z $(M+H)^+$ calcd for $C_{47}H_{46}N$: 624.3625, found: 624.3644.

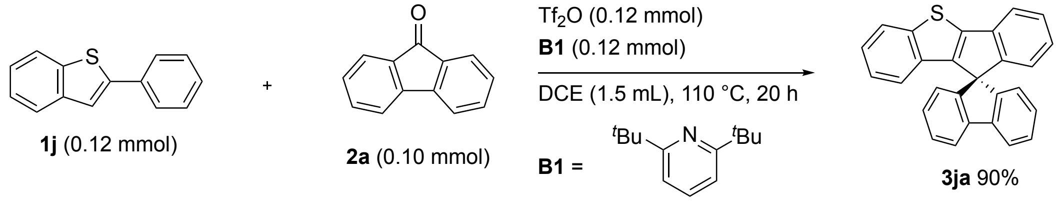

Synthesis of **3ja**: In a 20 mL screw cap test tube, 2-phenylbenzo[*b*]thiophene (**1j**, 0.12 mmol, 25 mg) and fluorenone (**2a**, 0.10 mmol, 18 mg) were placed with a magnetic stir bar. The reaction vessel was vacuumed and refilled with dry N_2. DCE (1.5 mL) and 2,6-di-*tert*-butylpyridine (**B1**, 0.12 mmol, 23 mg) were subsequently added by a syringe. The resulting mixture was stirred for 3 min. Tf_2O (0.12 mmol, 20 µL) was then added by a syringe. The reaction mixture was heated at 110 ˚C in an oil bath for 20 h. After cooling, sat. $NaHCO_3$ aq was added. The resulting mixture was extracted three times with $CHCl_3$ (10 mL x 3). The combined organic layer was filtered through a short pad of celite/alumina and concentrated in vacuo. The desired product spiro[benzo[*b*]indeno[2,1-*d*]thiophene-10,9'-fluorene] (**3ja**) was isolated by

column chromatography on silica gel using hexane/ toluene (1/0 to 3/1, v/v) as eluent followed by GPC (chloroform).

Spiro[benzo[*b*]indeno[2,1-*d*]thiophene-10,9'-fluorene] (**3ja**). Purified by column silica gel column chromatography with hexane/toluene (1/0 to 3/1, v/v) as eluent followed by GPC (chloroform): 34 mg (90%, 0.10 mmol scale); white solid; m.p. 185.2-187.2 °C; ^{1}H NMR ($CDCl_3$, 400 MHz): δ 7.91 (ddd, *J* = 7.6, 0.8, 0.8 Hz, 2H), 7.82 (ddd, *J* = 8.1, 0.8, 0.8 Hz, 1H), 7.62 (ddd, *J* = 7.6, 1.0, 1.0 Hz, 1H), 7.39 (ddd, *J* = 7.5, 7.5, 1.0 Hz, 2H), 7.35 (ddd, *J* = 7.6, 7.6, 1.1 Hz, 1H), 7.15 (ddd, *J* = 8.2, 7.1, 1.2 Hz, 1H), 7.09 (ddd, *J* = 7.5, 7.5, 1.1 Hz, 2H), 7.06 (ddd, *J* = 7.4, 7.4, 1.1 Hz, 1H), 6.97 (ddd, *J* = 8.0, 7.1, 1.0 Hz, 1H), 6.78 (ddd, *J* = 7.6, 0.9, 0.9 Hz, 2H), 6.74 (ddd, *J* = 7.6, 1.0, 1.0 Hz, 1H), 6.56 (ddd, *J* = 8.0, 1.2, 1.1 Hz, 1H). ^{13}C{^{1}H} NMR ($CDCl_3$, 100 MHz): δ 152.4, 145.9, 144.9, 144.2 143.9, 142.0, 139.1, 133.3, 128.1, 128.0, 127.9, 126.8, 124.8, 124.1, 124.0, 123.8, 123.7, 121.1, 120.3, 119.9, 63.9. HRMS (APCI) m/z $(M+H)^+$ calcd for $C_{27}H_{17}S$: 373.1045, found: 373.1037.

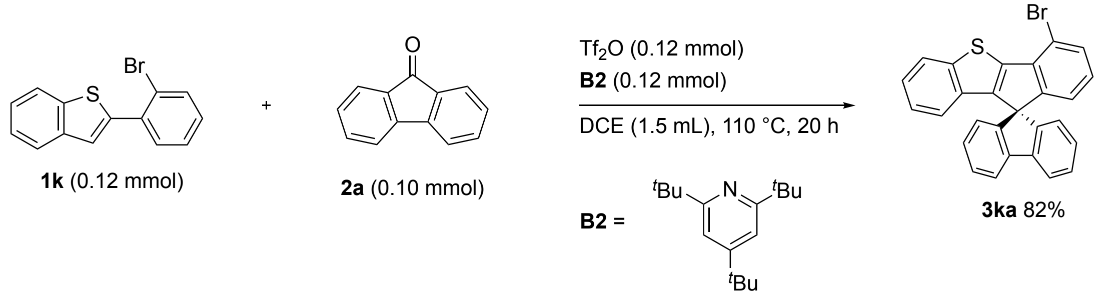

Synthesis of **3ka**: In a 20 mL screw cap test tube, 2-(2-bromophenyl)benzo[*b*]thiophene (**1k**, 0.12 mmol, 35 mg), fluorenone (**2a**, 0.10 mmol, 18 mg), and 2,4,6-tri-*tert*-butylpyridine (**B2**, 0.12 mmol, 30 mg) were placed with a magnetic stir bar. The reaction vessel was vacuumed and refilled with dry N_2. DCE (1.5 mL) was added by a syringe. The resulting mixture was stirred for 3 min. Tf_2O (0.12 mmol, 20 μL) was then added by a syringe. The reaction mixture was heated at 110 °C in an oil bath for 20 h. After cooling, sat. $NaHCO_3$ aq was added. The resulting mixture was extracted three times with $CHCl_3$ (10 mL x 3). The combined organic layer was filtered through a short pad of celite/alumina and concentrated in vacuo. The residue was purified by chromatography on silica gel using hexane/ethyl acetate (1/0 to 15/1 to 10/1, v/v) as eluent, again using hexane/toluene (1/0 to 2/1, v/v) as eluent. The desired product 4-bromospiro[benzo[*b*]indeno[2,1-*d*]thiophene-10,9'-fluorene] (**3ka**) was obtained by addition of pentane, decantation, and drying.

4-Bromospiro[benzo[*b*]indeno[2,1-*d*]thiophene-10,9'-fluorene] (**3ka**). Purified by silica gel column chromatography with hexane/ethyl acetate (1/0 to 15/1 to 10/1, v/v) then with hexane/toluene (1/0 to 2/1, v/v) as eluent. After concentration in vacuo, washing with pentane: 37 mg (82%, 0.10 mmol scale); white solid; m.p. 208.3-210.3 °C; ^{1}H NMR ($CDCl_3$, 400 MHz): δ 7.91 (d, J = 7.6 Hz, 2H), 7.86 (d, J = 8.1 Hz, 1H), 7.47 (dd, J = 8.0, 0.8 Hz, 1H), 7.40 (ddd, J = 7.5, 7.5, 1.0 Hz, 2H), 7.20-7.16 (m, 1H), 7.10 (ddd, J = 7.5, 7.5, 1.0 Hz, 2H), 7.01-6.97 (m, 1H), 6.92 (dd, J = 7.6, 7.6 Hz, 1H), 6.80 (d, J = 7.6 Hz, 2H), 6.67 (dd, J = 7.5, 0.8 Hz, 1H), 6.58 (d, J = 7.8 Hz, 1H). ^{13}C{^{1}H} NMR ($CDCl_3$, 100 MHz): δ 154.0, 145.33, 145.31, 144.8, 142.7, 141.9, 139.9, 132.7, 131.2, 128.4, 128.14, 128.05, 124.8, 124.4, 124.0, 123.7, 122.6, 121.2, 120.4, 114.4, 64.3. HRMS (APCI) m/z $(M+H)^+$ calcd for $C_{27}H_{16}BrS$: 451.0151, found: 451.0159.

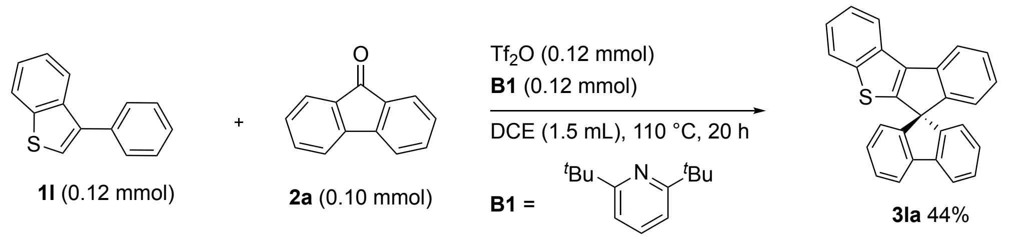

Synthesis of **3la**: In a 20 mL screw cap test tube, fluorenone (**2a**, 0.10 mmol, 18 mg) and 3-phenylbenzo[*b*]thiophene (**1l**, 0.12 mmol, 25 mg) were placed with a magnetic stir bar. The reaction vessel was vacuumed and refilled with dry N_2. DCE (1.5 mL) and 2,6-di-*tert*-butylpyridine (**B1**, 0.12 mmol, 23 mg) were subsequently added by a syringe. The resulting mixture was stirred for 3 min. Tf_2O (0.12 mmol, 20 µL) was then added by a syringe. The reaction mixture was heated at 110 ˚C in an oil bath for 20 h. After cooling, sat. $NaHCO_3$ aq was added. The resulting mixture was extracted three times with $CHCl_3$ (10 mL x 3). The combined organic layer was filtered through a short pad of celite/alumina and concentrated in vacuo. The desired product spiro[benzo[*b*]indeno[1,2-*d*]thiophene-6,9'-fluorene] (**3la**) was isolated by column chromatography on silica gel using hexane/toluene (1/0 to 3/1 to 2/1, v/v) as eluent followed by GPC (chloroform).

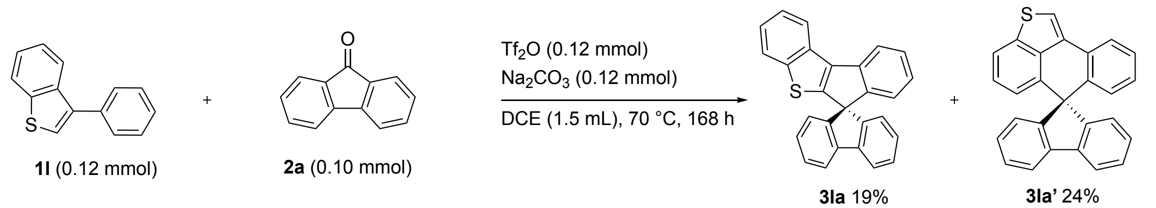

Synthesis of **3la** and **3la'**: In a schlenk tube with pressure resistance, 3-phenylbenzo[*b*]thiophene (**1l**, 0.12 mmol, 25 mg), fluorenone (**2a**, 0.10 mmol, 18 mg), and Na_2CO_3 (0.12 mmol, 13 mg) were placed

with a magnetic stir bar. The reaction vessel was vacuumed and refilled with dry N_2. DCE (1.5 mL) was subsequently added by a syringe. The resulting mixture was stirred for 3 min. Tf_2O (0.12 mmol, 20 µL) was then added by a syringe. The reaction mixture was heated at 70 ˚C in an oil bath for 168 h. After cooling, sat. $NaHCO_3$ aq was added. The resulting mixture was extracted three times with $CHCl_3$ (10 mL x 3). The combined organic layer was filtered through a short pad of celite/alumina and concentrated in vacuo. The products, spiro[benzo[*b*]indeno[1,2-*d*]thiophene-6,9'-fluorene] (**3la**) and spiro[anthra[1,9-*bc*]thiophene-6,9'-fluorene] (**3la'**) were isolated by column chromatography on silica gel using hexane as eluent followed by GPC (chloroform).

Spiro[benzo[*b*]indeno[1,2-*d*]thiophene-6,9'-fluorene] (**3la**). Purified by column silica gel column chromatography with hexane/toluene (1/0 to 3/1 to 2/1, v/v) as eluent followed by GPC (chloroform): 16 mg (44%, 0.10 mmol scale); white solid; m.p. 106.4-108.4 ºC; ^{1}H NMR ($CDCl_3$, 400 MHz): δ 8.27 (ddd, J = 8.0, 0.9, 0.9 Hz, 1H), 7.91 (ddd, J = 7.5, 1.0, 1.0 Hz, 1H), 7.85 (ddd, J = 7.6, 0.9, 0.9 Hz, 2H), 7.80 (ddd, J = 8.1, 0.8, 0.8 Hz, 1H), 7.54 (ddd, J = 8.0, 7.2, 1.0 Hz, 1H), 7.40 (ddd, J = 7.5, 7.5, 1.1 Hz, 2H), 7.38 (ddd, J = 7.9, 6.6, 0.8 Hz, 1H), 7.37 (ddd, J = 8.2, 7.1, 1.2 Hz, 1H), 7.14 (ddd, J = 7.5, 7.5, 1.1 Hz, 2H), 7.03 (ddd, J = 7.5, 7.5, 1.1 Hz, 1H), 6.85 (ddd, J = 7.6, 0.8, 0.8 Hz, 2H), 6.70 (ddd, J = 7.4, 0.9, 0.9 Hz, 1H). ^{13}C{^{1}H} NMR ($CDCl_3$, 100 MHz): δ 152.7, 151.8, 146.9, 145.6, 141.8, 141.2, 139.4, 133.0, 128.4, 128.1, 127.7, 125.6, 125.0, 124.3, 124.13, 124.08, 123.6, 122.2, 120.4, 119.3, 64.6. HRMS (APCI) m/z $(M+H)^+$ calcd for $C_{27}H_{17}S$: 373.1045, found: 373.1026.

Spiro[anthra[1,9-*bc*]thiophene-6,9'-fluorene] (**3la'**). Purified by column silica gel column chromatography with hexane as eluent followed by GPC (chloroform): 8.9 mg (24%, 0.10 mmol scale); white solid; m.p. 185.2-187.2 ºC; ^{1}H NMR ($CDCl_3$, 400 MHz): δ 7.94 (dd, J = 7.9, 1.1 Hz, 1H), 7.84 (d, J = 7.6 Hz, 2H), 7.76 (s, 1H), 7.67 (dd, J = 8.0, 0.7 Hz, 1H), 7.36 (ddd, J = 7.5, 7.5, 1.0 Hz, 2H), 7.26-7.22 (m, 1H), 7.14 (ddd, J = 7.5, 7.5, 1.1 Hz, 2H), 7.10 (dd, J = 7.7, 7.7 Hz, 1H), 6.98-6.93 (m, 3H), 6.48 (dd, J = 8.0, 1.0 Hz, 1H), 6.39 (dd, J = 7.5, 0.7 Hz, 1H). ^{13}C{^{1}H} NMR ($CDCl_3$, 100 MHz): δ 156.2, 140.0, 139.3, 139.2, 136.8, 135.2, 130.7, 130.0, 129.3, 128.5, 128.2, 127.7, 127.2, 126.3, 125.7, 123.8, 122.0, 120.5, 120.1, 117.3, 59.9. HRMS (APCI) m/z $(M+H)^+$ calcd for $C_{27}H_{17}S$: 373.1045, found: 373.1062.

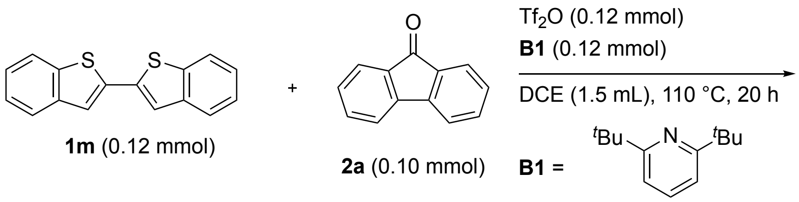

Synthesis of **3ma**: In a 20 mL screw cap test tube, fluorenone (**2a**, 0.10 mmol, 18 mg) and 2,2'-bibenzo[*b*]thiophene (**1m**, 0.12 mmol, 32 mg) were placed with a magnetic stir bar. The reaction vessel was vacuumed and refilled with dry N_2. DCE (1.5 mL) and 2,6-di-*tert*-butylpyridine (**B1**, 0.12 mmol, 23 mg) were subsequently added by a syringe. The resulting mixture was stirred for 3 min. Tf_2O (0.12 mmol, 20 μL) was then added by a syringe. The reaction mixture was heated at 110 ˚C in an oil bath for 20 h. After cooling, sat. $NaHCO_3$ aq was added. The resulting mixture was extracted three times with $CHCl_3$ (10 mL x 3). The combined organic layer was filtered through a short pad of celite/alumina and concentrated in vacuo. The desired product **3ma** was isolated by column chromatography on silica gel using hexane/ethyl acetate/toluene (10/0/0 to 10/1/0 to 5/1/0 to 1/0/2, v/v/v) as eluent followed by GPC (chloroform).

3ma. Purified by column silica gel column chromatography with hexane/ethyl acetate/toluene (10/0/0 to 10/1/0 to 5/1/0 to 1/0/2, v/v/v) as eluent followed by GPC (chloroform): 28 mg (66%, 0.10 mmol scale); white solid; m.p. > 300 ºC; ^{1}H NMR ($CDCl_3$, 400 MHz): δ 7.98 (ddd, J = 7.6, 0.8, 0.8 Hz, 2H), 7.82 (ddd, J = 8.1, 0.8, 0.8 Hz, 2H), 7.41 (ddd, J = 7.5, 7.5, 1.1 Hz, 2H), 7.13 (ddd, J = 8.2, 7.1, 1.2 Hz, 2H), 7.07 (ddd, J = 7.5, 7.5, 1.1 Hz, 2H), 6.99 (ddd, J = 8.0, 7.1, 1.0 Hz, 2H), 6.82 (ddd, J = 7.6, 0.8, 0.8 Hz, 2H), 6.60 (ddd, J = 8.0, 1.1, 0.7 Hz, 2H). ^{13}C{^{1}H} NMR ($CDCl_3$, 100 MHz): δ 148.3, 143.9, 143.6, 141.9, 139.3, 133.2, 128.4, 128.1, 125.1, 124.0, 123.8, 123.7, 120.6, 120.4, 62.5. HRMS (APCI) m/z $(M+H)^+$ calcd for $C_{29}H_{17}S_2$: 429.0766, found: 429.0744.

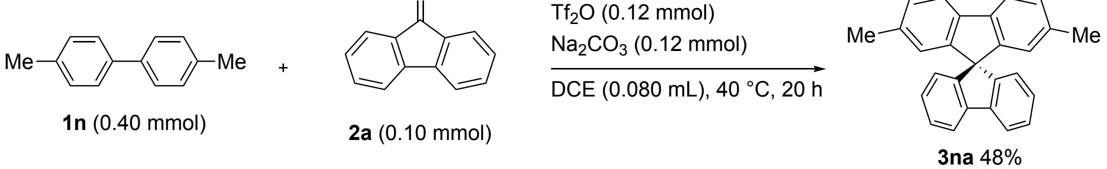

Synthesis of **3na**: In a 18*40 mm screw vial, 4,4'-dimethyl-1,1'-biphenyl (**1n**, 0.40 mmol, 73 mg), fluorenone (**2a**, 0.10 mmol, 18 mg), and Na_2CO_3 (0.12 mmol, 13 mg) were placed with a magnetic stir

bar. DCE (0.080 mL) and Tf_2O (0.12 mmol, 20 μL) were subsequently added by a syringe. The vial was flushed with N_2 and sealed. The reaction mixture was heated at 40 ˚C in an oil bath for 20 h. After cooling, sat. $NaHCO_3$ aq was added. The resulting mixture was extracted five times with $CHCl_3$ (2 mL x 5). The combined organic layer was filtered through a short pad of celite/alumina and concentrated in vacuo. The desired product 2,7-dimethyl-9,9'-spirobi[fluorene] (**3na**) was isolated by column chromatography on silica gel using hexane/toluene (1/0 to 2/1, v/v) as eluent followed by GPC (chloroform).

2,7-Dimethyl-9,9'-spirobi[fluorene] (**3na**). Purified by column silica gel column chromatography with hexane/toluene (1/0 to 2/1, v/v) as eluent followed by GPC (chloroform): 16 mg (48%, 0.10 mmol scale); white solid; m.p. 226.6-228.6 ºC; ^{1}H NMR ($CDCl_3$, 400 MHz): δ 7.84 (dd, J = 7.8, 0.6 Hz, 2H), 7.67 (d, J = 7.7 Hz, 2H), 7.36 (dd, J = 7.5, 7.5 Hz, 2H), 7.13 (dd, J = 8.1, 8.1 Hz, 2H), 7.11 (dd, J = 7.5, 7.5 Hz, 2H), 6.75 (d, J = 7.6 Hz, 2H), 6.50 (s, 2H), 2.17 (s, 6H). ^{13}C{^{1}H} NMR ($CDCl_3$, 100 MHz): δ 149.4, 148.9, 141.8, 139.4, 137.4, 128.8, 127.9, 127.7, 124.6, 124.3, 120.0, 119.5, 65.8, 21.6. HRMS (APCI) m/z $(M+H)^+$ calcd for $C_{27}H_{21}$: 345.1638, found: 345.1642.

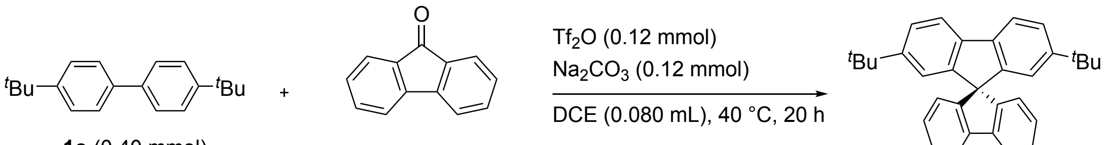

1o (0.40 mmol) **2a** (0.10 mmol) **3oa** 60%

Synthesis of **3oa**: In a 18*40 mm screw vial, 4,4'-oxybis(methylbenzene) (**1o**, 0.40 mmol, 79 mg), fluorenone (**2a**, 0.10 mmol, 18 mg), and Na_2CO_3 (0.12 mmol, 13 mg) were placed with a magnetic stir bar. DCE (0.080 mL) and Tf_2O (0.12 mmol, 20 μL) were subsequently added by a syringe. The vial was flushed with N_2 and sealed. The reaction mixture was heated at 40 ˚C in an oil bath for 20 h. After cooling, sat. $NaHCO_3$ aq was added. The resulting mixture was extracted five times with $CHCl_3$ (2 mL x 5). The combined organic layer was filtered through a short pad of celite/alumina and concentrated in vacuo. The desired product 2,7-di-*tert*-butyl-9,9'-spirobi[fluorene] (**3oa**) was isolated by column chromatography on silica gel using hexane/toluene (1/0 to 3/1, v/v) as eluent followed by GPC (chloroform).

2,7-Di-*tert*-butyl-9,9'-spirobi[fluorene] (**3oa**). Purified by column silica gel column chromatography with hexane/toluene (1/0 to 3/1, v/v) as eluent followed by GPC (chloroform): 26 mg (60%, 0.10 mmol scale); white solid; m.p. 172.9-174.9 ºC; ^{1}H NMR ($CDCl_3$, 400 MHz): δ 7.85 (ddd, J = 1.0, 1.0, 7.6 Hz, 2H), 7.71 (d, J = 8.0 Hz, 2H), 7.38-7.34 (m, 4H), 7.09 (ddd, J = 7.5, 7.5, 1.1 Hz, 2H), 6.73 (d, J = 7.6

Hz, 2H), 6.64 (d, J = 1.7 Hz, 2H), 1.14 (s, 18H). $^{13}C\{^1H\}$ NMR ($CDCl_3$, 100 MHz): δ 150.8, 149.7, 149.1, 141.9, 139.4, 127.8, 127.5, 124.8, 124.4, 120.8, 119.9, 119.1, 66.4, 35.0, 31.6. HRMS (APCI) m/z $(M+H)^+$ calcd for $C_{33}H_{33}$: 429.2577, found: 429.2591.

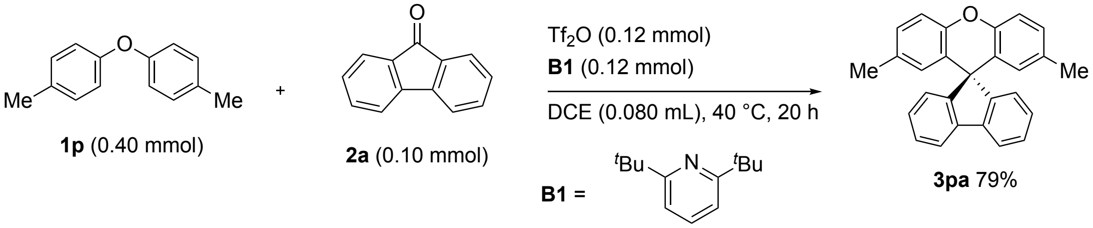

Synthesis of **3pa**: In a 18*40 mm screw vial, 4,4'-oxybis(methylbenzene) (**1p**, 0.40 mmol, 79 mg), fluorenone (**2a**, 0.10 mmol, 18 mg), and 2,6-di-*tert*-butylpyridine (**B1**, 0.12 mmol, 23 mg) were placed with a magnetic stir bar. DCE (0.080 mL) and Tf_2O (0.12 mmol, 20 μL) were subsequently added by a syringe. The vial was flushed with N_2 and sealed. The reaction mixture was heated at 40 ˚C in an oil bath for 20 h. After cooling, sat. $NaHCO_3$ aq was added. The resulting mixture was extracted five times with $CHCl_3$ (2 mL x 5). The combined organic layer was filtered through a short pad of celite/alumina and concentrated in vacuo. The desired product 2',7'-dimethylspiro[fluorene-9,9'-xanthene] (**3pa**) was isolated by column chromatography on silica gel using hexane/toluene (1/0 to 5/2, v/v) as eluent.

2',7'-Dimethylspiro[fluorene-9,9'-xanthene] (**3pa**). Purified by column silica gel column chromatography with hexane/toluene (1/0 to 5/2, v/v) as eluent: 29 mg (79%, 0.10 mmol scale); white solid; m.p. 170.6-172.6 °C; ^1H NMR ($CDCl_3$, 400 MHz): δ 7.81 (d, J = 7.6 Hz, 2H), 7.38 (dd, J = 7.5, 7.5 Hz, 2H), 7.22 (dd, J = 7.6, 7.6 Hz, 2H), 7.17 (d, J = 7.5 Hz, 2H), 7.09 (d, J = 8.3 Hz, 2H), 6.96 (dd, J = 8.3, 2.0 Hz, 2H), 6.15 (s, 2H), 2.01 (s, 6H). $^{13}C\{^1H\}$ NMR ($CDCl_3$, 100 MHz): δ 155.2, 149.6, 139.8, 132.4, 129.0, 128.5, 127.9, 127.8, 125.9, 124.6, 120.0, 116.6, 54.5, 20.8. HRMS (APCI) m/z $(M+H)^+$ calcd for $C_{27}H_{21}O$: 361.1587, found: 361.1614.

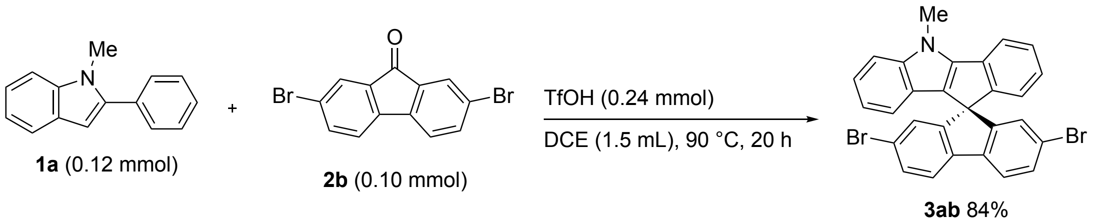

Synthesis of **3ab**: In a 20 mL screw cap test tube, 1-methyl-2-phenyl-indole (**1a**, 0.12 mmol, 25 mg) and

fluorenone (**2b**, 0.10 mmol, 34 mg) were placed with a magnetic stir bar. The reaction vessel was vacuumed and refilled with dry N_2. DCE (1.5 mL) was added by a syringe. The resulting mixture was stirred for 3 min. TfOH (0.24 mmol, 21 μL) was then added by a measuring pipette. The reaction mixture was heated at 90 ˚C in an oil bath for 20 h. After cooling, sat. $NaHCO_3$ aq was added. The resulting mixture was extracted three times with $CHCl_3$ (10 mL x 3). The combined organic layer was filtered through a short pad of celite/alumina and concentrated in vacuo. The desired product 2,7-dibromo-5'-methyl-5'*H*-spiro[fluorene-9,10'-indeno[1,2-*b*]indole] (**3ab**) was isolated by column chromatography on silica gel using hexane/ethyl acetate (1/0 to 15/1, v/v) as eluent.

2,7-Dibromo-5'-methyl-5'*H*-spiro[fluorene-9,10'-indeno[1,2-*b*]indole] (**3ab**). Purified by column silica gel column chromatography with hexane/ethyl acetate (1/0 to 15/1, v/v): 45 mg (84%, 0.10 mmol scale); white solid; m.p. >300 ºC; ^{1}H NMR ($CDCl_3$, 400 MHz): δ 7.70 (d, J = 7.5 Hz, 1H), 7.69 (d, J = 8.1 Hz, 2H), 7.47 (dd, J = 8.1, 1.8 Hz, 2H), 7.38 (d, J = 8.3 Hz, 1H), 7.34 (ddd, J = 7.6, 7.6, 1.0 Hz, 1H), 7.15 (ddd, J = 8.3, 7.1, 1.1 Hz, 1H), 7.03 (ddd, J = 7.6, 7.6, 1.0 Hz, 1H), 6.90 (d, J = 1.7 Hz, 2H), 6.90-6.86 (m, 1H), 6.69-6.65 (m, 2H), 4.16 (s, 3H). ^{13}C{^{1}H} NMR ($CDCl_3$, 100 MHz): δ 152.7, 149.6, 145.2, 142.3, 139.8, 135.5, 131.2, 128.0, 127.2, 126.4, 124.4, 122.5, 122.4, 121.93, 121.87, 121.5, 120.1, 118.6, 118.2, 110.1, 60.4, 31.5. HRMS (APCI) m/z $(M+H)^+$ calcd for $C_{28}H_{18}Br_2N$: 524.9728, found: 524.9753.

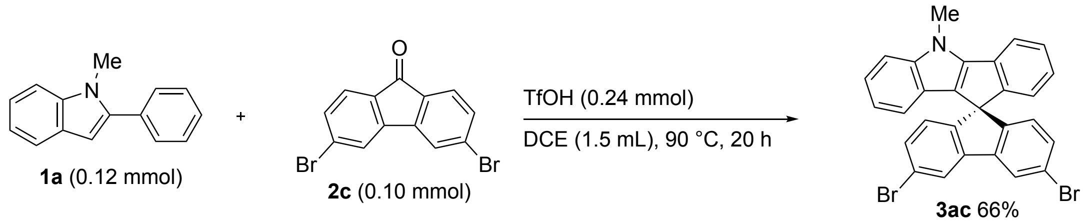

1a (0.12 mmol) **2c** (0.10 mmol) **3ac** 66%

Synthesis of **3ac**: In a 20 mL screw cap test tube, 1-methyl-2-phenyl-indole (**1a**, 0.12 mmol, 25 mg) and 3,6-dibromo-9*H*-fluoren-9-one (**2c**, 0.10 mmol, 34 mg) were placed with a magnetic stir bar. The reaction vessel was vacuumed and refilled with dry N_2. DCE (1.5 mL) was added by a syringe. The resulting mixture was stirred for 3 min. TfOH (0.24 mmol, 21 μL) was then added by a measuring pipette. The reaction mixture was heated at 90 ˚C in an oil bath for 20 h. After cooling, sat. $NaHCO_3$ aq was added. The resulting mixture was extracted three times with $CHCl_3$ (10 mL x 3). The combined organic layer was filtered through a short pad of celite/alumina and concentrated in vacuo. The desired product 5'-methyl-2,7-dioctyl-5'*H*-spiro[fluorene-9,10'-indeno[1,2-*b*]indole] (**3ac**) was isolated by column chromatography on silica gel using hexane/toluene (1/0 to 5/2, v/v) as eluent.

3,6-Dibromo-5'-methyl-5'*H*-spiro[fluorene-9,10'-indeno[1,2-*b*]indole] (**3ac**). Purified by column silica gel column chromatography with hexane/toluene (1/0 to 5/2, v/v): 35 mg (66%, 0.10 mmol scale); white solid; m.p. 230.6-232.6 °C; ^{1}H NMR ($CDCl_3$, 400 MHz): δ 7.95 (d, J = 1.8 Hz, 2H), 7.68 (d, J = 7.6 Hz, 1H), 7.36 (d, J = 8.3 Hz, 1H), 7.32 (ddd, J = 7.6, 7.6, 0.9 Hz, 1H), 7.20 (dd, J = 8.1, 1.8 Hz, 2H), 7.14 (ddd, J = 8.2, 7.1, 1.1 Hz, 1H), 7.01 (ddd, J = 7.6, 7.6, 0.9 Hz, 1H), 6.87 (ddd, J = 7.8, 7.8, 0.6 Hz, 1H), 6.653 (dd, J = 7.9, 7.9 Hz, 2H), 6.652 (d, J = 8.1 Hz, 2H), 4.15 (s, 3H). ^{13}C{^{1}H} NMR ($CDCl_3$, 100 MHz): δ 152.9, 146.8, 145.1, 142.6, 142.3, 135.6, 131.3, 127.9, 126.3, 125.6, 123.2, 123.6, 122.5, 122.4, 121.9, 121.8, 120.2, 118.6, 118.1, 110.0, 60.0, 31.5. HRMS (APCI) m/z $(M+H)^+$ calcd for $C_{28}H_{18}Br_2N$: 525.9801, found: 525.9789.

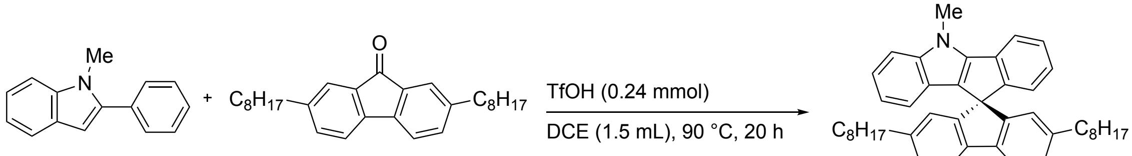

1a (0.12 mmol) **2d** (0.10 mmol) **3ad** 65%

Synthesis of **3ad**: In a 20 mL screw cap test tube, 1-methyl-2-phenyl-indole (**1a**, 0.12 mmol, 25 mg) and 2,7-dioctyl-9*H*-fluoren-9-one (**2d**, 0.10 mmol, 40 mg) were placed with a magnetic stir bar. The reaction vessel was vacuumed and refilled with dry N_2. DCE (1.5 mL) was added by a syringe. The resulting mixture was stirred for 3 min. TfOH (0.24 mmol, 21 μL) was then added by a measuring pipette. The reaction mixture was heated at 90 ˚C in an oil bath for 20 h. After cooling, sat. $NaHCO_3$ aq was added. The resulting mixture was extracted three times with $CHCl_3$ (10 mL x 3). The combined organic layer was filtered through a short pad of celite/alumina and concentrated in vacuo. The desired product 5'-methyl-2,7-dioctyl-5'*H*-spiro[fluorene-9,10'-indeno[1,2-*b*]indole] (**3ad**) was isolated by column chromatography on silica gel using hexane/ethyl acetate (1/0 to 20/1, v/v) as eluent followed by GPC (chloroform).

5'-Methyl-2,7-dioctyl-5'*H*-spiro[fluorene-9,10'-indeno[1,2-*b*]indole] (**3ad**). Purified by column silica gel column chromatography with hexane/ethyl acetate (1/0 to 20/1, v/v) as eluent followed by GPC (chloroform): 38 mg (65%, 0.10 mmol scale); oil; ^{1}H NMR ($CDCl_3$, 400 MHz): δ 7.71 (d, J = 7.9 Hz, 2H), 7.70 (dd, J = 8.2, 8.2 Hz, 1H), 7.36 (d, J = 7.9 Hz, 1H), 7.29 (dd, J = 7.6, 7.6 Hz, 1H), 7.13 (d, J = 7.7 Hz, 2H), 7.11 (dd, J = 8.2, 8.2 Hz, 1H), 6.98 (dd, J = 7.9, 7.9 Hz, 1H), 6.82 (dd, J = 7.9, 7.9 Hz, 1H), 6.69 (dd, J = 6.9, 6.9 Hz, 2H), 6.53 (s, 2H), 4.17 (s, 3H), 2.38 (t, J = 8.0 Hz, 4H), 1.42-1.39 (m, 4H),

1.22-1.14 (m, 20H), 0.83 (t, J = 6.8 Hz, 3H), 0.82 (t, J = 7.1 Hz, 3H). $^{13}C\{^1H\}$ NMR ($CDCl_3$, 100 MHz): δ 155.1, 147.5, 144.9, 142.24, 142.21, 139.7, 135.6, 127.7, 127.1, 126.0, 124.8, 124.5, 123.7, 122.8, 121.4, 119.7, 119.4, 119.1, 117.7, 109.7, 60.6, 36.1, 31.9, 31.6, 31.5, 29.49, 29.48, 29.3, 22.8, 14.2. HRMS (APCI) m/z $(M+H)^+$ calcd for $C_{44}H_{52}N$: 594.4094, found: 594.4075.

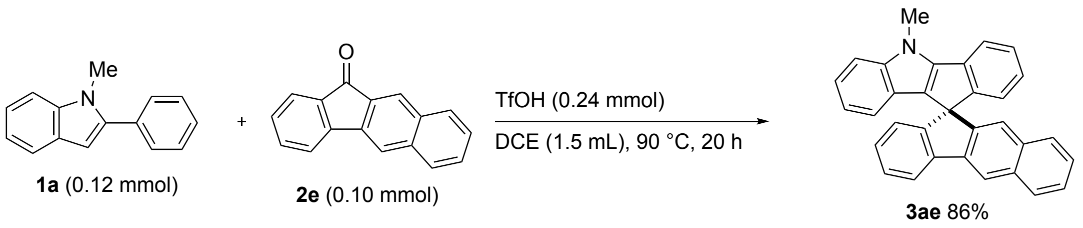

Synthesis of **3ae**: In a 20 mL screw cap test tube, 1-methyl-2-phenyl-indole (**1a**, 0.12 mmol, 25 mg) and 11*H*-benzo[*b*]fluoren-11-one (**2e**, 0.10 mmol, 23 mg) were placed with a magnetic stir bar. The reaction vessel was vacuumed and refilled with dry N_2. DCE (1.5 mL) was added by a syringe. The resulting mixture was stirred for 3 min. TfOH (0.24 mmol, 21 μL) was then added by a measuring pipette. The reaction mixture was heated at 90 ˚C in an oil bath for 20 h. After cooling, sat. $NaHCO_3$ aq was added. The resulting mixture was extracted three times with $CHCl_3$ (10 mL x 3). The combined organic layer was filtered through a short pad of celite/alumina and concentrated in vacuo. The desired product 5'-methyl-5'*H*-spiro[benzo[*b*]fluorene-11,10'-indeno[1,2-*b*]indole] (**3ae**) was isolated by column chromatography on silica gel using hexane/ethyl acetate (1/0 to 5/1, v/v) as eluent.

5'-Methyl-5'*H*-spiro[benzo[*b*]fluorene-11,10'-indeno[1,2-*b*]indole] (**3ae**). Purified by column silica gel column chromatography with hexane/ethyl acetate (1/0 to 5/1, v/v) as eluent: 36 mg (86%, 0.10 mmol scale); white solid; m.p. 262.6-264.6 °C; ^1H NMR ($CDCl_3$, 400 MHz): δ 8.29 (s, 1H), 8.01 (d, J = 7.6 Hz, 1H), 7.93 (d, J = 7.8 Hz, 1H), 7.71 (d, J = 7.6 Hz, 1H), 7.49 (d, J = 8.1 Hz, 1H), 7.40 (ddd, J = 7.2, 7.2, 1.2 Hz, 1H), 7.38 (ddd, J = 5.7, 5.7, 1.4 Hz, 1H), 7.35 (d, J = 8.0 Hz, 1H), 7.30 (ddd, J = 7.5, 7.5, 1.1 Hz, 1H), 7.30-7.26 (m, 1H), 7.25 (s, 1H), 7.11-7.07 (m, 2H), 6.97 (ddd, J = 7.6, 7.6, 1.0 Hz, 1H), 6.81 (d, J = 6.6 Hz, 1H), 6.97 (ddd, J = 7.0, 7.0, 0.9 Hz, 1H), 6.70 (d, J = 7.5 Hz, 1H), 6.67 (d, J = 7.7 Hz, 1H), 4.17 (s, 3H). $^{13}C\{^1H\}$ NMR ($CDCl_3$, 100 MHz): δ 155.7, 148.4, 145.7, 144.8, 142.3,141.3, 140.5, 135.4, 133.8, 133.7, 128.5, 128.3, 128.2, 128.0, 127.4, 126.1, 125.7, 125.5, 125.4, 124.6, 124.1, 122.6, 122.4, 121.6, 120.8, 119.9, 118.8, 118.2, 117.9, 109.9, 60.2, 31.5. HRMS (APCI) m/z $(M+H)^+$ calcd for $C_{32}H_{22}N$: 420.1747, found: 420.1735.

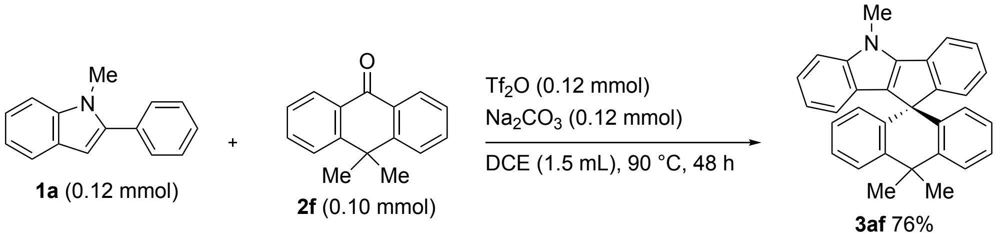

Synthesis of **3af**: In a 20 mL screw cap test tube, 1-methyl-2-phenyl-indole (**1a**, 0.12 mmol, 25 mg), 11*H*-benzo[*b*]fluoren-11-one (**2f**, 0.10 mmol, 22 mg), and Na_2CO_3 (0.12 mmol, 13 mg) were placed with a magnetic stir bar. The reaction vessel was vacuumed and refilled with dry N_2. DCE (1.5 mL) was added by a syringe. The resulting mixture was stirred for 3 min. Tf_2O (0.12 mmol, 20 μL) was then added by a syringe. The reaction mixture was heated at 90 ˚C in an oil bath for 48 h. After cooling, sat. $NaHCO_3$ aq was added. The resulting mixture was extracted three times with $CHCl_3$ (10 mL x 3). The combined organic layer was filtered through a short pad of celite/alumina and concentrated in vacuo. The desired product 5',10,10-trimethyl-5'*H*,10*H*-spiro[anthracene-9,10'-indeno[1,2-*b*]indole**]** (**3af**) was isolated by column chromatography on silica gel using hexane/ethyl acetate (1/0 to 10/1 to 5/1, v/v) as eluent followed by GPC (chloroform).

5',10,10-Trimethyl-5'*H*,10*H*-spiro[anthracene-9,10'-indeno[1,2-*b*]indole] (**3af**). Purified by column silica gel column chromatography with hexane/ethyl acetate (1/0 to 10/1 to 5/1, v/v) as eluent followed by GPC (chloroform): 31 mg (76%, 0.10 mmol scale); white solid; m.p. >300 °C; ^{1}H NMR ($CDCl_3$, 400 MHz): δ 7.67 (d, J = 7.6 Hz, 1H), 7.60 (d, J = 8.0 Hz, 2H), 7.36 (d, J = 8.3 Hz, 1H), 7.25 (dd, J = 7.5, 7.5 Hz, 1H), 7.16 (dd, J = 7.2, 7.2 Hz, 2H), 7.12 (dd, J = 8.2, 8.2 Hz, 1H), 7.00 (dd, J = 7.6, 7.6 Hz, 1H), 6.94 (dd, J = 7.9, 0.7 Hz, 1H), 6.88-6.82 (m, 2H), 6.79 (dd, J = 7.8, 7.8 Hz, 2H), 6.48 (d, J = 8.0 Hz, 2H), 4.17 (s, 3H), 1.96 (s, 3H), 1.95 (s, 3H). ^{13}C{^{1}H} NMR ($CDCl_3$, 100 MHz): δ 164.2, 143.8, 143.3, 142.4, 143.9, 134.2, 133.9, 127.8, 127.2, 126.9, 126.8, 126.5, 126.3, 126.0, 122.3, 121.7, 119.9, 118.8, 118.0, 109.8, 52.7, 37.5, 36.3, 35.3, 31.5. HRMS (APCI) m/z $(M+H)^+$ calcd for $C_{31}H_{26}N$: 412.2060, found: 412.2072.

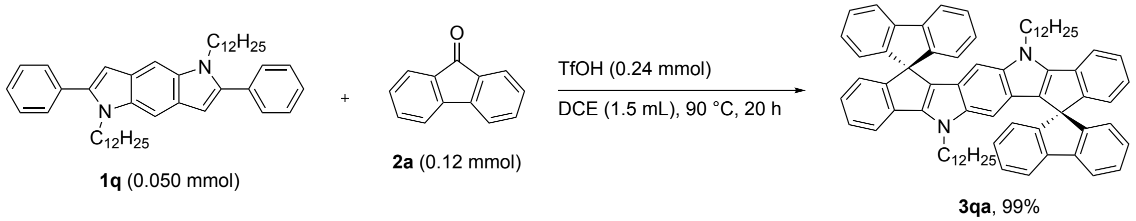

Synthesis of **3qa**: In a Schlenk tube with pressure resistance, 1,5-didodecyl-2,6-diphenyl-1,5-dihydropyrrolo[2,3-*f*]indole (**1q**, 0.050 mmol, 19 mg) and fluorenone (**2a**, 0.12 mmol, 22 mg) were placed with a magnetic stir bar. The reaction vessel was vacuumed and refilled with dry N_2. DCE (1.5 mL) was added by a syringe. The resulting mixture was stirred for 3 min. TfOH (0.24 mmol, 21 μL) was then added by a measuring pipette. The reaction mixture was heated at 90 ˚C in an oil bath for 20 h. After cooling, sat. $NaHCO_3$ aq was added. The resulting mixture was extracted three times with toluene (10 mL x 3). The combined organic layer was filtered through a short pad of celite/alumina and concentrated in vacuo. The desired product 5',12'-didodecyl-5',12'-dihydrodispiro[fluorene-9,7'-indeno[1,2-*b*]indeno[2',1':4,5]pyrrolo[2,3-*f*]indole-14',9''-fluorene] (**3qa**) was isolated by column chromatography on silica gel (Silica gel 60 N spherical neutral, Kanto Chemical Co.) using hexane/toluene (1/0 to 2/1, v/v) as eluent. The single crystals of **3qa** suitable for X-ray analysis were grown from CD_2Cl_2.
Note: This compound rapidly decomposed in $CHCl_3$ solution.

5',12'-Didodecyl-5',12'-dihydrodispiro[fluorene-9,7'-indeno[1,2-*b*]indeno[2',1':4,5]pyrrolo[2,3-*f*]indole-14',9''-fluorene] (**3qa**). Purified by column silica gel column chromatography with hexane/toluene (1/0 to 2/1, v/v): 52 mg (99%, 0.050 mmol scale); yellow solid; m.p. 211.3-213.3 ºC; ^{1}H NMR (CD_2Cl_2, 400 MHz): δ 7.86 (d, J = 7.6 Hz, 4H), 7.45 (d, J = 7.6 Hz, 2H), 7.29 (dd, J = 7.5, 7.5 Hz, 4H), 7.15 (dd, J = 7.6, 7.6 Hz, 2H), 6.98 (dd, J = 7.4, 7.4 Hz, 4H), 6.83 (dd, J = 7.5, 7.5 Hz, 2H), 6.62 (d, J = 7.6 Hz, 4H), 6.48 (d, J = 7.5 Hz, 2H), 6.32 (s, 2H), 4.14 (t, J = 7.1 Hz, 4H), 1.66-1.61 (m, 4H), 1.18-1.09 (m, 36H), 0.80 (t, J = 7.0 Hz, 6H). $^{13}C\{^1H\}$ NMR (CD_2Cl_2, 100 MHz): δ 154.3, 147.7, 145.2, 141.9, 139.0, 135.9, 127.53, 127.51, 127.3, 125.3, 123.5, 123.3, 122.5, 120.1, 119.8, 117.9, 97.1, 60.6, 44.8, 32.0, 30.3, 29.67, 29.66, 29.6, 29.44, 29.39, 29.3, 26.9, 22.7, 13.9. HRMS (FAB) m/z (M)$^+$ calcd for $C_{72}H_{76}N_2$: 968.6009, found: 968.6032.

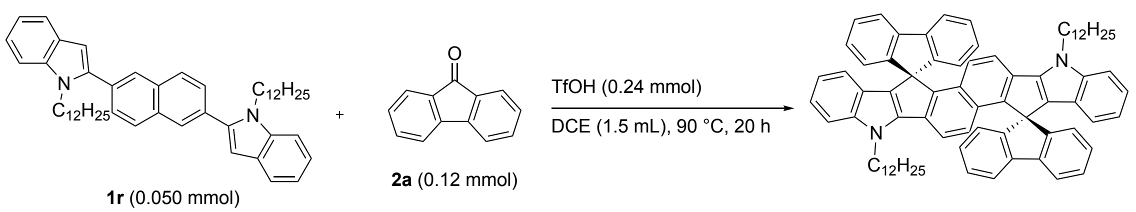

1r (0.050 mmol) **2a** (0.12 mmol) **3ra**, 58%

Synthesis of **3ra**: In a 20 mL screw cap test tube, 2,6-bis(1-dodecyl-1*H*-indol-2-yl)naphthalene (**1r**, 0.050

mmol, 22 mg) and fluorenone (**2a**, 0.12 mmol, 22 mg) were placed with a magnetic stir bar. The reaction vessel was vacuumed and refilled with dry N_2. DCE (1.5 mL) was added by a syringe. The resulting mixture was stirred for 3 min. TfOH (0.24 mmol, 21 μL) was then added by a measuring pipette. The reaction mixture was heated at 90 ˚C in an oil bath for 20 h. After cooling, sat. $NaHCO_3$ aq was added. The resulting mixture was extracted three times with toluene (10 mL x 3). The combined organic layer was filtered through a short pad of celite/alumina and concentrated in vacuo. The desired product **3ra** was isolated by column chromatography on silica gel using hexane/toluene (1/0 to 2/3, v/v) as eluent followed by GPC (chloroform). The single crystals of **3ra** suitable for X-ray analysis were grown from DCE/$MeNO_2$.

3ra. Purified by column silica gel column chromatography with hexane/toluene (1/0 to 2/3, v/v) as eluent followed by GPC (chloroform): 30 mg (58%, 0.050 mmol scale); yellow solid; m.p. >300 °C; ^{1}H NMR ($CDCl_3$, 400 MHz): δ 8.03 (d, J = 7.6 Hz, 4H), 7.41 (d, J = 7.5 Hz, 2H), 7.40 (dd, J = 7.6, 7.6 Hz, 4H), 7.26 (d, J = 8.3 Hz, 2H), 7.05 (ddd, J = 7.4, 7.4, 1.0 Hz, 4H), 7.01-6.97 (m, 2H), 6.88 (d, J = 8.6 Hz, 2H), 6.73 (dd, J = 7.2, 7.2 Hz, 2H), 6.71 (d, J = 7.6 Hz, 4H), 6.53 (d, J = 7.8 Hz, 2H), 4.36 (t, J = 7.4 Hz, 4H), 1.95-1.87 (m, 4H), 1.29-1.22 (m, 36H), 0.88 (t, J = 7.0 Hz, 6H). ^{13}C{^{1}H} NMR ($CDCl_3$, 100 MHz): δ 148.4, 147.8, 143.9, 141.6, 141.4, 133.2, 128.8, 128.0, 127.7, 125.6, 124.2, 124.0, 122.2, 121.0, 120.5, 119.7, 118.3, 117.9, 110.0, 61.5, 45.1, 32.1, 31.1, 29.73 (2C), 29.68, 29.6, 29.50, 29.48, 27.3, 22.8, 14.3. HRMS (FAB) m/z $(M)^+$ calcd for $C_{76}H_{78}N_2$: 1018.6160, found: 1018.6158.

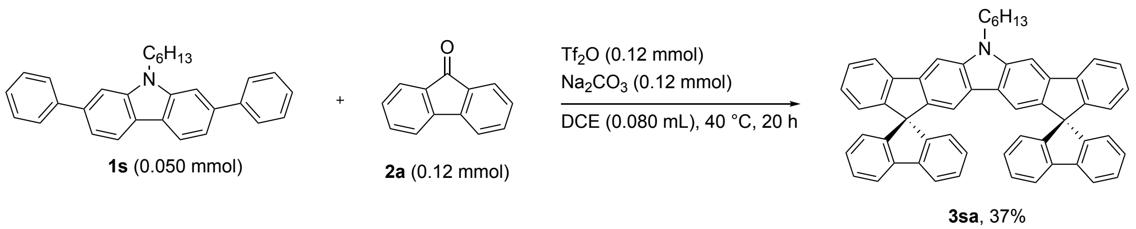

Synthesis of **3sa**: In a 18*40 mm screw vial, 9-hexyl-2,7-diphenyl-9H-carbazole (**1s**, 0.050 mmol, 20 mg), fluorenone (**2a**, 0.12 mmol, 22 mg), and Na_2CO_3 (0.12 mmol, 13 mg) were placed with a magnetic stir bar. DCE (0.080 mL) and Tf_2O (0.12 mmol, 20 μL) were subsequently added by a syringe. The vial was flushed with N_2 and sealed. The reaction mixture was heated at 40 ˚C in an oil bath for 20 h. After cooling, sat. $NaHCO_3$ aq was added. The resulting mixture was extracted five times with $CHCl_3$ (2 mL x 5). The combined organic layer was filtered through a short pad of celite/alumina and concentrated in vacuo. The residue was purified by column chromatography on silica gel using hexane/toluene (1/0 to

3/1 to 2/1, v/v) as eluent. The obtained solid was further purified by filtration through a short pad of alumina using chloroform as eluent. After evaporation, addition of cold Et_2O, decantation, and drying afforded the desired product 6'-hexyl-6'*H*-dispiro[fluorene-9,12'-diindeno[1,2-*b*:2',1'-*h*]carbazole-15',9''-fluorene] (**3sa**). The single crystals of **3sa** suitable for X-ray analysis were grown from DCE/CH_3CN.

6'-Hexyl-6'*H*-dispiro[fluorene-9,12'-diindeno[1,2-*b*:2',1'-*h*]carbazole-15',9''-fluorene] (**3sa**). Purified by column silica gel column chromatography with hexane/toluene (1/0 to 3/1 to 2/1, v/v) as eluent followed by washing with cold Et_2O.: 14 mg (37%, 0.050 mmol scale); white solid; m.p. >300 °C; ^{1}H NMR ($CDCl_3$, 400 MHz): δ 7.92 (d, J = 7.6 Hz, 2H), 7.78 (d, J = 7.6 Hz, 4H), 7.74 (s, 2H), 7.36 (ddd, J = 7.5, 7.5, 0.8 Hz, 2H), 7.28 (ddd, J = 7.6, 7.6, 0.8 Hz, 4H), 7.07 (ddd, J = 7.5, 7.5, 0.8 Hz, 2H), 7.05 (s, 2H), 7.28 (ddd, J = 7.5, 7.5, 0.8 Hz, 4H), 6.69 (d, J = 7.5 Hz, 2H), 6.66 (d, J = 7.6 Hz, 4H), 4.45 (t, J = 7.3 Hz, 2H), 2.08-2.00 (m, 2H), 1.62-1.55 (m, 2H), 1.51-1.36 (m, 4H), 0.96 (t, J = 7.1 Hz, 3H). ^{13}C{^{1}H} NMR ($CDCl_3$, 100 MHz): δ 149.9, 149.8, 142.4, 141.7, 141.6, 140.0, 139.9, 127.9, 127.73, 127.70, 127.6, 124.24, 124.17, 123.4, 120.0, 119.8, 115.7, 99.7, 65.5, 43.6, 31.9, 29.2, 27.3, 22.8, 14.3. HRMS (APCI) m/z $(M+H)^+$ calcd for $C_{56}H_{42}N$: 728.3312, found: 728.3289.

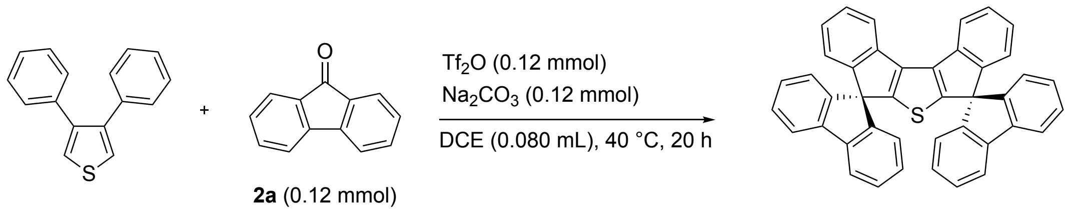

1t (0.050 mmol) **2a** (0.12 mmol) **3ta**, 97%

Synthesis of **3ta**: In a 18*40 mm screw vial, 3,4-diphenylthiophene (**1t**, 0.050 mmol, 12 mg), fluorenone (**2a**, 0.12 mmol, 22 mg), and Na_2CO_3 (0.12 mmol, 13 mg) were placed with a magnetic stir bar. DCE (0.080 mL) and Tf_2O (0.12 mmol, 20 μL) were subsequently added by a syringe. The vial was flushed with N_2 and sealed. The reaction mixture was heated at 40 ˚C in an oil bath for 20 h. After cooling, sat. $NaHCO_3$ aq was added. The resulting mixture was extracted five times with $CHCl_3$ (2 mL x 5). The combined organic layer was filtered through a short pad of celite/alumina and concentrated in vacuo. The desired product 6'-hexyl-6'*H*- dispiro[fluorene-9,5'-diindeno[2,1-*b*:1',2'-*d*]thiophene-7',9''-fluorene] (**3ta**) was isolated by column chromatography on silica gel using hexane/toluene (1/0 to 3/1 to 5/2, v/v) as eluent. The single crystals of **3ta** suitable for X-ray analysis were grown from $CHCl_3$/hexane.

Dispiro[fluorene-9,5'-diindeno[2,1-*b*:1',2'-*d*]thiophene-7',9''-fluorene] (**3ta**). Purified by column

silica gel column chromatography with hexane/toluene (1/0 to 3/1 to 5/2, v/v): 30 mg (97%, 0.050 mmol scale); white solid; m.p. >300 °C; ^{1}H NMR ($CDCl_3$, 400 MHz): δ 8.06 (d, J = 7.3 Hz, 2H), 7.74 (d, J = 7.6 Hz, 4H), 7.44 (dd, J = 7.5, 7.5 Hz, 2H), 7.32 (dd, J = 7.5, 7.5 Hz, 4H), 7.12 (dd, J = 7.5, 7.5 Hz, 4H), 7.05 (dd, J = 7.6, 7.6 Hz, 2H), 6.91 (dd, J = 7.6, 0.8 Hz, 4H), 6.69 (d, J = 7.5 Hz, 2H). ^{13}C{^{1}H} NMR ($CDCl_3$, 100 MHz): δ 154.8, 152.7, 147.4, 141.5, 139.6, 138.9, 128.2, 128.1, 127.8, 125.8, 124.0, 123.9, 120.8, 120.2, 64.9. HRMS (APCI) m/z $(M+H)^+$ calcd for $C_{42}H_{25}S$: 561.1671, found: 561.1675.

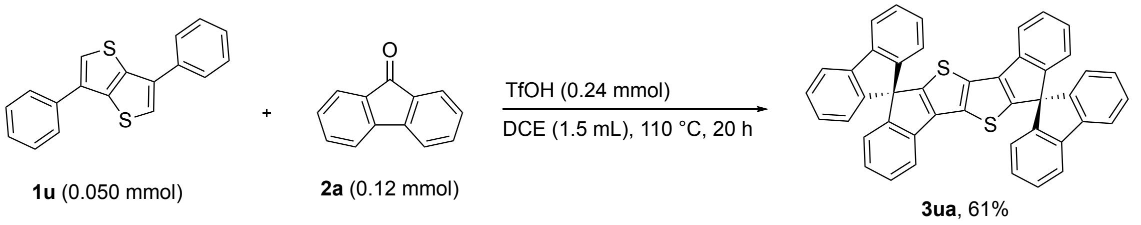

Synthesis of **3ua**: In a schlenk tube with pressure resistance, 3,6-diphenylthieno[3,2-*b*]thiophene (**1u**, 0.050 mmol, 15 mg) and fluorenone (**2a**, 0.12 mmol, 22 mg) were placed with a magnetic stir bar. The reaction vessel was vacuumed and refilled with dry N_2. DCE (1.5 mL) was subsequently added by a syringe. The resulting mixture was stirred for 3 min. TfOH (0.24 mmol, 21 μL) was then added by a measuring pipette. The reaction mixture was heated at 110 ˚C in an oil bath for 20 h. After cooling, sat. $NaHCO_3$ aq was added. The resulting mixture was extracted three times with $CHCl_3$ (10 mL x 3). The combined organic layer was filtered through a short pad of celite/alumina and concentrated in vacuo. The residue was purified by column chromatography on silica gel using hexane/toluene (1/0 to 1/1, v/v). The desired product dispiro[fluorene-9,6'-indeno[2,1-*b*]indeno[1',2':4,5]thieno[2,3-*d*]thiophene-12',9''-fluorene] (**3ua**) was obtained by addition of hexane, decantation, and drying.

Dispiro[fluorene-9,6'-indeno[2,1-*b*]indeno[1',2':4,5]thieno[2,3-*d*]thiophene-12',9''-fluorene] (**3ua**). Purified by column silica gel column chromatography with hexane/toluene (1/0 to 1/1, v/v) followed by washing with hexane: 19 mg (61%, 0.050 mmol scale); pale red solid; m.p. >300 °C; ^{1}H NMR ($CDCl_3$, 400 MHz): δ 7.87 (d, J = 7.6 Hz, 4H), 7.45 (d, J = 7.4 Hz, 4H), 7.42 (ddd, J = 7.5, 7.5, 1.0 Hz, 2H), 7.28 (ddd, J = 7.5, 7.5, 0.9 Hz, 2H), 7.18 (ddd, J = 7.5, 7.5, 1.0 Hz, 4H), 7.00 (ddd, J = 7.6, 7.6, 1.0 Hz, 2H), 6.93 (dd, J = 7.6 Hz, 4H), 6.69 (d, J = 7.6 Hz, 2H). ^{13}C{^{1}H} NMR ($CDCl_3$, 100 MHz): δ 152.5, 151.2, 147.3, 141.7, 139.6, 137.6, 134.8, 128.5, 128.2, 127.8, 126.0, 124.1, 123.6, 120.4, 119.5, 65.2. HRMS (APCI) m/z $(M+H)^+$ calcd for $C_{44}H_{25}S_2$: 617.1392, found: 617.1397.

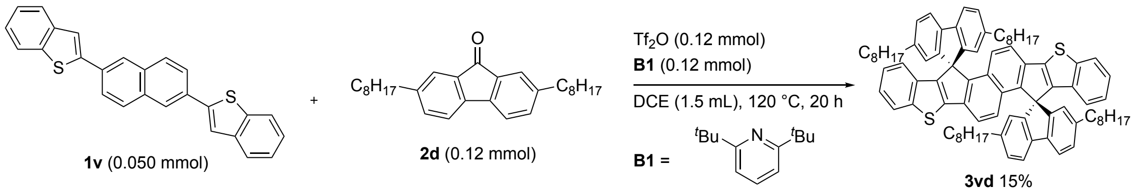

Synthesis of **3vd**: In a Schlenk tube with pressure resistance, 2,6-bis(benzo[*b*]thiophen-2-yl)naphthalene (**1v**, 0.050 mmol, 20 mg) and fluorenone (**2d**, 0.12 mmol, 49 mg) were placed with a magnetic stir bar. DCE (1.5 mL) and 2,6-di-*tert*-butylpyridine (**B1**, 0.12 mmol, 23 mg) were subsequently added by a syringe. The resulting mixture was stirred for 3 min. Tf_2O (0.12 mmol, 20 μL) was then added by a syringe. The reaction mixture was heated at 120 ˚C in an oil bath for 20 h. After cooling, sat. $NaHCO_3$ aq was added. The resulting mixture was extracted three times with $CHCl_3$ (10 mL x 3). The combined organic layer was filtered through a short pad of celite/alumina and concentrated in vacuo. The desired product **3vd** was isolated by column chromatography on silica gel using hexane/toluene (1/0 to 3/1 to 2/1/ to 0/1, v/v) as eluent followed by GPC (ethyl acetate then chloroform).

3vd. Purified by column silica gel column chromatography with hexane/toluene (1/0 to 3/1 to 2/1/ to 0/1, v/v) as eluent followed by GPC (ethyl acetate then chloroform): 9 mg (15%, 0.050 mmol scale); pale yellow solid; m.p. 218.3-220.3 ºC; ^{1}H NMR ($CDCl_3$, 400 MHz): δ 7.90 (d, J = 7.8 Hz, 4H), 7.73 (d, J = 8.0 Hz, 2H), 7.39 (d, J = 8.4 Hz, 2H), 7.21 (dd, J = 7.9, 1.3 Hz, 4H), 7.07 (dd, J = 8.1, 7.1, 1.1 Hz, 2H), 6.95-6.93 (m, 2H), 6.92 (d, J = 8.3 Hz, 2H), 6.57 (d, J = 8.0 Hz, 2H), 6.45 (d, J = 0.7 Hz, 4H), 2.40-2.28 (m, 8H), 1.37-1.30 (m, 8H), 1.18-1.03 (m, 40H), 0.76 (t, J = 7.0 Hz, 12H). ^{13}C{^{1}H} NMR ($CDCl_3$, 100 MHz): δ 147.2, 146.5, 146.4, 143.9, 143.2, 142.7, 139.5, 136.5, 133.4, 128.7, 128.2, 124.7, 124.4, 124.1, 123.6, 123.5, 120.8, 120.1, 119.5, 64.8, 36.0, 31.9, 31.3, 29.4, 29.3, 29.2, 22.7, 14.2. HRMS (APCI) m/z $(M + H)^+$ calcd for $C_{84}H_{93}S_2$: 1165.6726, found: 1165.6713.

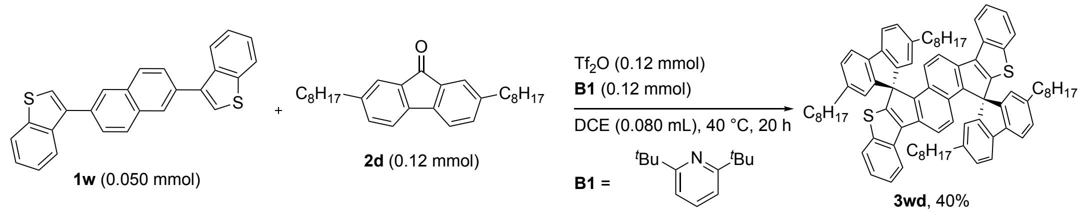

Synthesis of **3wd**: In a 18*40 mm screw vial, 2,6-bis(benzo[b]thiophen-3-yl)naphthalene (**1w**, 0.050 mmol, 20 mg), fluorenone (**2d**, 0.12 mmol, 49 mg), and 2,6-di-*tert*-butylpyridine (**B1**, 0.12 mmol, 23 mg) were placed with a magnetic stir bar. DCE (0.080 mL) and Tf_2O (0.12 mmol, 20 μL) were

subsequently added by a syringe. The vial was flushed with N_2 and sealed. The reaction mixture was heated at 40 ˚C in an oil bath for 20 h. After cooling, sat. $NaHCO_3$ aq was added. The resulting mixture was extracted five times with $CHCl_3$ (2 mL x 5). The combined organic layer was filtered through a short pad of celite/alumina and concentrated in vacuo. The desired product **3wd** was isolated by column chromatography on silica gel using hexane/toluene (1/0 to 2/1, v/v) as eluent followed by GPC (chloroform). The single crystals of **3wd** suitable for X-ray analysis were grown from DCE/$MeNO_2$.

3wd. Purified by column silica gel column chromatography with hexane/toluene (1/0 to 2/1, v/v) as eluent followed by GPC (chloroform): 24 mg (40%, 0.050 mmol scale); pale yellow solid; m.p. 71.2-73.2 °C; ^{1}H NMR ($CDCl_3$, 400 MHz): δ 8.11 (d, J = 8.0 Hz, 2H), 7.86 (d, J = 7.8 Hz, 4H), 7.75 (d, J = 8.7 Hz, 2H), 7.72 (d, J = 8.0 Hz, 2H), 7.41 (dd, J = 7.2, 7.2 Hz, 2H), 7.26 (dd, J = 7.2, 7.2 Hz, 2H), 7.23 (d, J = 7.6 Hz, 4H), 6.96 (d, J = 8.8 Hz, 2H), 6.53 (s, 4H), 2.39 (t, J = 7.0 Hz, 4H), 2.38 (t, J = 7.0 Hz, 4H), 1.39-1.37 (m, 8H), 1.12-1.07 (m, 40H), 0.71 (t, J = 7.0 Hz, 12H). ^{13}C{^{1}H} NMR ($CDCl_3$, 100 MHz): δ 153.9, 147.7, 146.7, 145.4, 143.0, 140.1, 139.3, 136.8, 133.0, 128.5, 128.1, 124.7, 124.2, 124.0, 123.8 (2C), 121.8, 120.2, 119.3, 65.3, 36.1, 31.9, 31.6, 29.5, 29.4, 29.3, 22.7, 14.2. HRMS (FAB) m/z $(M)^+$ calcd for $C_{84}H_{92}N_2$: 1164.6635, found: 1164.6648.

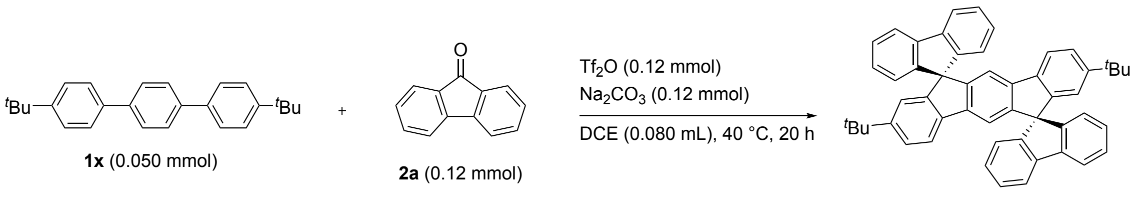

1x (0.050 mmol)

2a (0.12 mmol)

3xa 54%

Synthesis of **3xa**: In a 18*40 mm screw vial, 4,4''-di-*tert*-butyl-1,1':4',1''-terphenyl (**1x**, 0.050 mmol, 17 mg), fluorenone (**2a**, 0.12 mmol, 22 mg), and Na_2CO_3 (0.12 mmol, 13 mg) were placed with a magnetic stir bar. DCE (0.080 mL) and Tf_2O (0.12 mmol, 20 μL) were subsequently added by a syringe. The vial was flushed with N_2 and sealed. The reaction mixture was heated at 40 ˚C in an oil bath for 20 h. After cooling, sat. $NaHCO_3$ aq was added. The resulting mixture was extracted five times with $CHCl_3$ (2 mL x 5). The combined organic layer was filtered through a short pad of celite/alumina and concentrated in vacuo. The desired product 2',8'-di-*tert*-butyldispiro[fluorene-9,6'-indeno[1,2-*b*]fluorene-12',9''-fluorene] (**3xa**) was isolated by column chromatography on silica gel using hexane/ethylacetate (1/0 to 15/1, v/v), hexane/toluene (1/0 to 2/1, v/v), and finally hexane/toluene (1/0 to 3/1, v/v). The single crystals of **3xa** suitable for X-ray analysis were grown from $CHCl_3$/hexane.

2',8'-Di-*tert*-butyldispiro[fluorene-9,6'-indeno[1,2-*b*]fluorene-12',9''-fluorene] (**3xa**). Purified by column silica gel column chromatography with hexane/ethyl acetate (1/0 to 15/1, v/v), hexane/toluene (1/0 to 2/1, v/v), and finally hexane/toluene (1/0 to 3/1, v/v): 18 mg (54%, 0.050 mmol scale); white solid; m.p. >300 ºC; ^{1}H NMR ($CDCl_3$, 400 MHz): δ 7.91 (d, J = 7.6 Hz, 4H), 7.41 (ddd, J = 7.5, 7.5, 1.0 Hz, 4H), 7.40 (d, J =8.1 Hz, 2H), 7.23 (d, J = 8.1, 1.8 Hz, 2H), 7.14 (ddd, J = 7.5, 7.5, 1.0 Hz, 4H), 7.03 (s, 2H), 6.79 (d, J = 7.6 Hz, 4H), 6.63 (d, J = 1.5 Hz, 2H), 1.09 (s, 18H). ^{13}C{^{1}H} NMR ($CDCl_3$, 100 MHz): δ 151.0, 149.6, 149.1, 148.9, 141.9, 141.5, 139.3, 127.9, 127.7, 124.9, 124.5, 120.6, 120.0, 119.4, 115.4, 66.1, 34.9, 31.5. HRMS (APCI) m/z $(M+H)^+$ calcd for $C_{52}H_{43}$: 667.3359, found: 667.3387.

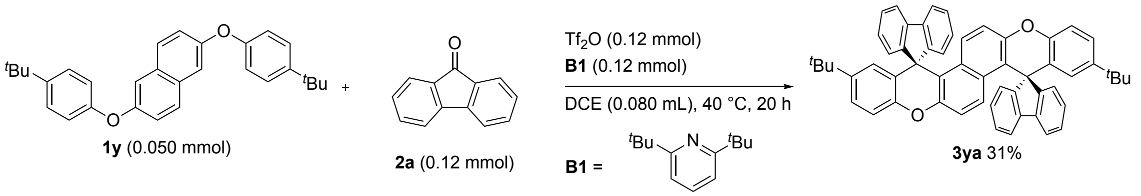

Synthesis of **3ya**: In a 18*40 mm screw vial, 2,6-bis(4-(*tert*-butyl)phenoxy)naphthalene (**1y**, 0.050 mmol, 21 mg), fluorenone (**2a**, 0.12 mmol, 22 mg), and **B1** (0.12 mmol, 23 mg) were placed with a magnetic stir bar. DCE (0.080 mL) and Tf_2O (0.12 mmol, 20 µL) were subsequently added by a syringe. The vial was flushed with N_2 and sealed. The reaction mixture was heated at 40 ˚C in an oil bath for 20 h. After cooling, sat. $NaHCO_3$ aq was added. The resulting mixture was extracted five times with $CHCl_3$ (2 mL x 5). The combined organic layer was filtered through a short pad of celite/alumina and concentrated in vacuo. The residue was purified by column chromatography on silica gel using hexane/toluene (1/0 to 5/2, v/v) as eluent. After evaporation, addition of hexane, decantation, and drying, afforded the desired product 3',11'-di-*tert*-butyldispiro[fluorene-9,5'-xantheno[2,1-*a*]xanthene-13',9''-fluorene] (**3ya**).

3',11'-Di-*tert*-butyldispiro[fluorene-9,5'-xantheno[2,1-*a*]xanthene-13',9''-fluorene] (**3ya**). Purified by column silica gel column chromatography with hexane/toluene (1/0 to 5/2, v/v) as eluent followed by washing with hexane: 12 mg (31%, 0.050 mmol scale); white solid; m.p. >300 ºC; ^{1}H NMR ($CDCl_3$, 400 MHz): δ 7.94 (d, J = 7.6 Hz, 4H), 7.39 (dd, J = 7.4, 7.4 Hz, 4H), 7.14 (dd, J = 7.2, 7.2 Hz, 4H), 7.05 (d, J = 7.6 Hz, 4H), 7.01 (dd, J = 8.6, 2.3 Hz, 2H), 6.91 (d, J = 8.5 Hz, 2H), 6.80 (d, J = 9.5 Hz, 2H), 6.75 (d, J = 9.5 Hz, 2H), 6.14 (d, J = 2.2 Hz, 2H), 0.89 (s, 18H). ^{13}C{^{1}H} NMR ($CDCl_3$, 100 MHz): δ 156.8, 148.6, 147.1, 145.4, 139.4, 129.6, 128.6, 127.7, 126.7, 125.7, 124.8, 124.5, 123.7, 120.3, 118.2, 115.6, 114.4, 54.3, 34.1, 31.2. HRMS (APCI) m/z $(M+H)^+$ calcd for $C_{56}H_{45}O_2$: 749.3414, found: 749.3410.

References

(S1) C. M. So, C. P. Lau, A. S. C. Chan and F. Y. Kwong, *J. Org. Chem.,* 2008, **73**, 7731.

(S2) M. S. Eom, J. Noh, H.-S. Kim, S. Yoo, M. S. Han and S. Lee, *Org. Lett.,* 2016, **18**, 1720.

(S3) P. Tosatti and A. Pfaltz, *Angew. Chem., Int. Ed.,* 2017, **56**, 4579.

(S4) T. Qi, Y. Guo, Y. Liu, H. Xi, H. Zhang, X. Gao, Y. Liu, K. Lu, C. Du, G. Yu and D. Zhu, *Chem. Commun.,* 2008, **2008**, 6227.

(S5) Y. Kurota, Y. Sakamoto, T. Suzuki, E. Kayahara and S. Yamago, *J. Org. Chem.,* 2016, **81**, 3356.

(S6) P. Wang, J. Min, J. C. Nwachukwu, V. Cavett, K. E. Carlson, P. Guo, M. Zhu, Y. Zheng, C. Dong, J. A. Katzenellenbogen, K. W. Nettles and H.-B. Zhou, *J. Med. Chem.,* 2012, **55**, 2324.

(S7) T. Iitsuka, K. Hirano, T. Satoh and M. Miura, *J. Org. Chem.,* 2015, **80**, 2804.

(S8) (*a*) Y. Wang, S. Zou, J. Guo, H. Zhang, G. Lai, H. Xie, C. Yang, H. Li and W. Hu, *RSC Adv.,* 2015, **5**, 31018; (*b*) A. F. Littke, C. Dai and G. C. Fu, *J. Am. Chem. Soc.,* 2000, **122**, 4020.

(S9) H. A. Yemam, A. Mahl, J. S. Tinkham, J. T. Koubek, U. Greife and A. Sellinger, *Chem. Eur. J.,* 2017, **23**, 8921.

(S10) A. K. Mishra, A. Verma and S. Biswas, *J. Org. Chem.,* 2017, **82**, 3403.

(S11) H. Tsuji, Y. Yokoi, C. Mitsui, L. Ilies, Y. Sato and E. Nakamura, *Chem. Asian J.,* 2009, **4**, 655.

(S12) S. Naoe, T. Saito, M. Uchiyama, S. Oishi, N. Fujii and H. Ohno, *Org. Lett.,* 2015, **17**, 1774.

[^{1}H and ^{13}C{^{1}H} NMR Spectra of **1q**]

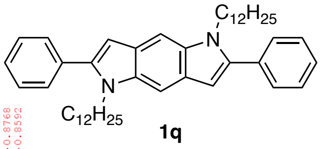

1q

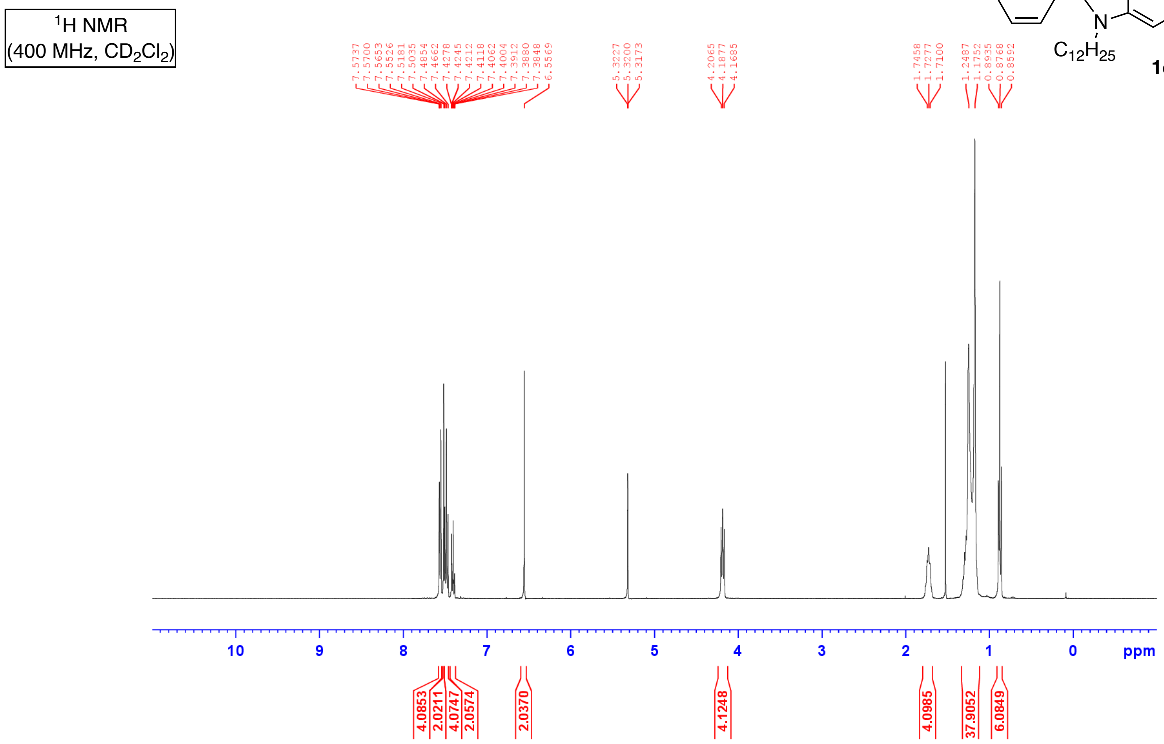

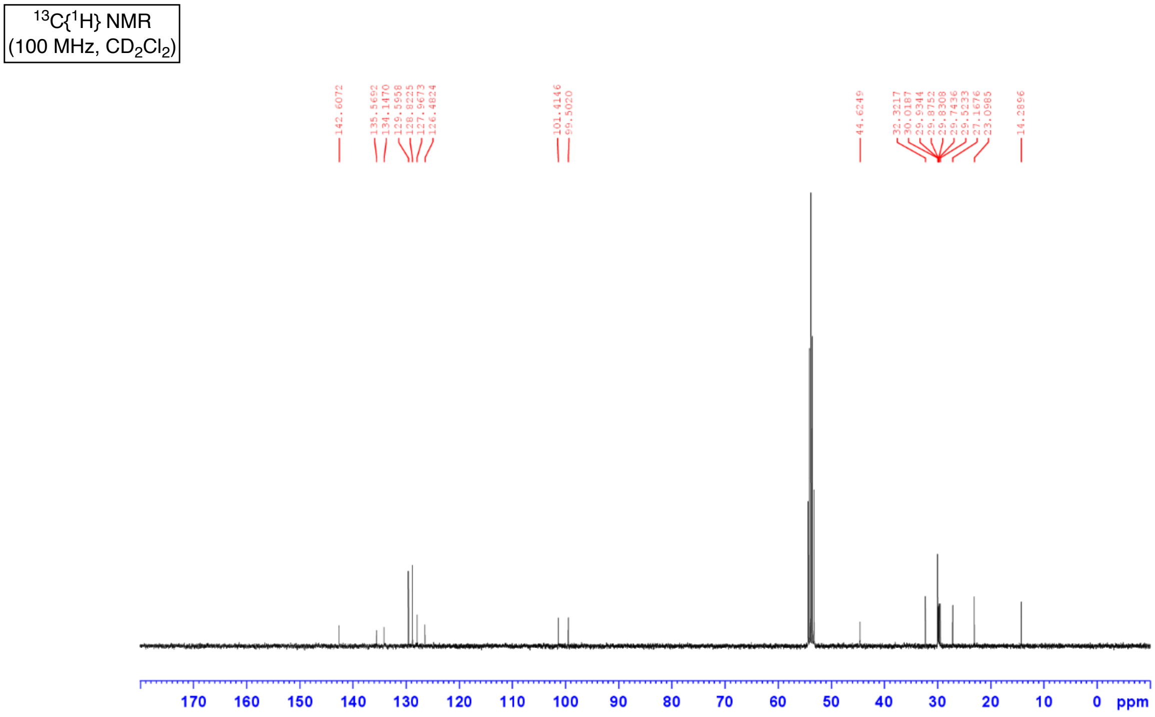

[^{1}H and ^{13}C{^{1}H} NMR Spectra of **2d**]

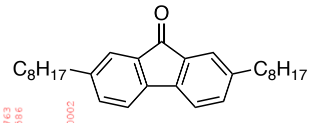

2d

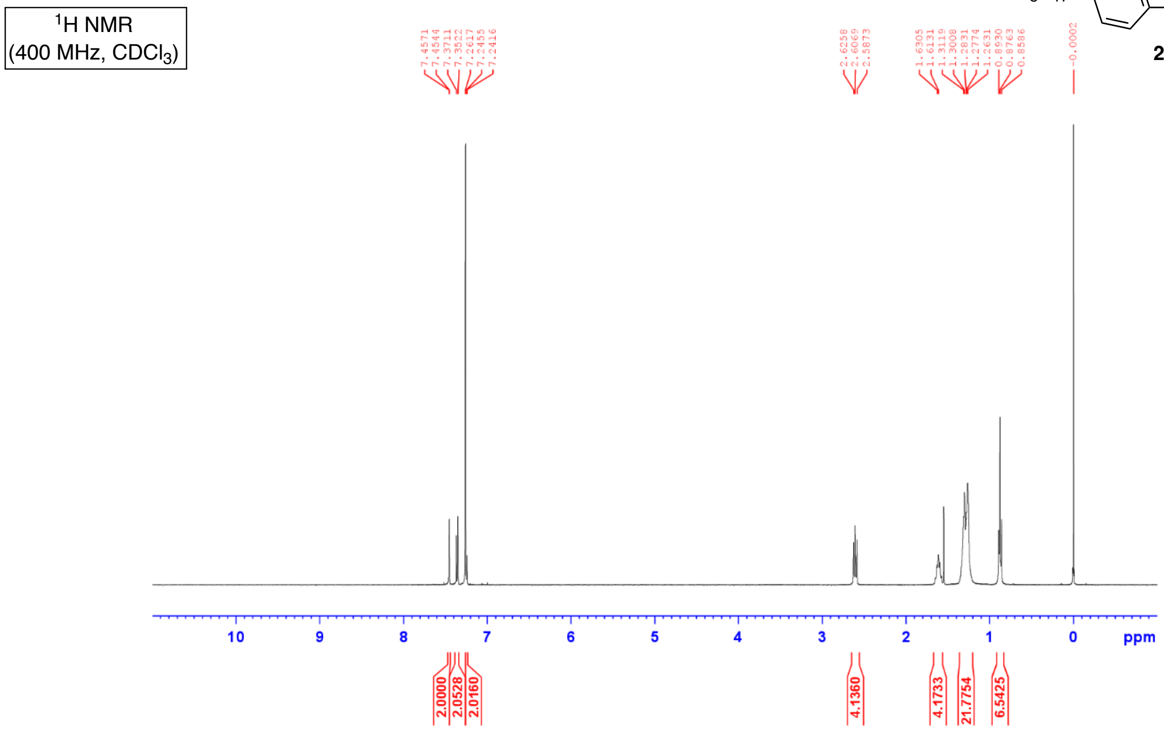

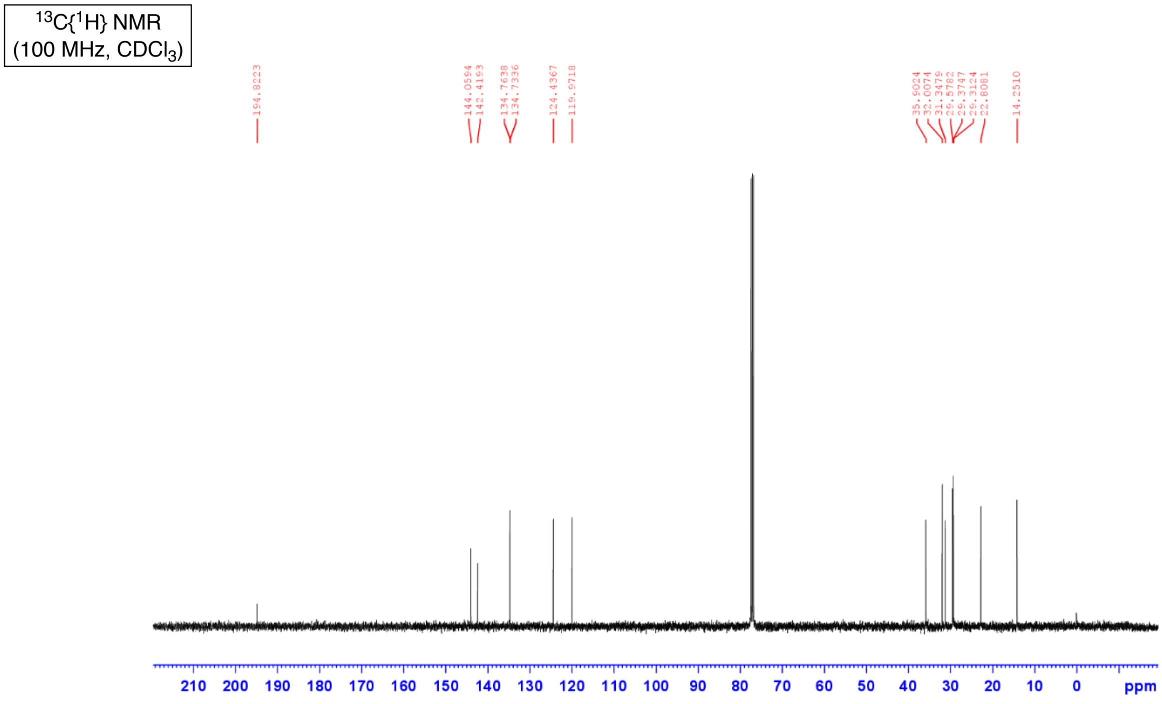

[^{1}H and ^{13}C{^{1}H} NMR Spectra of **3aa**]

^{1}H NMR
(400 MHz, $CDCl_3$)

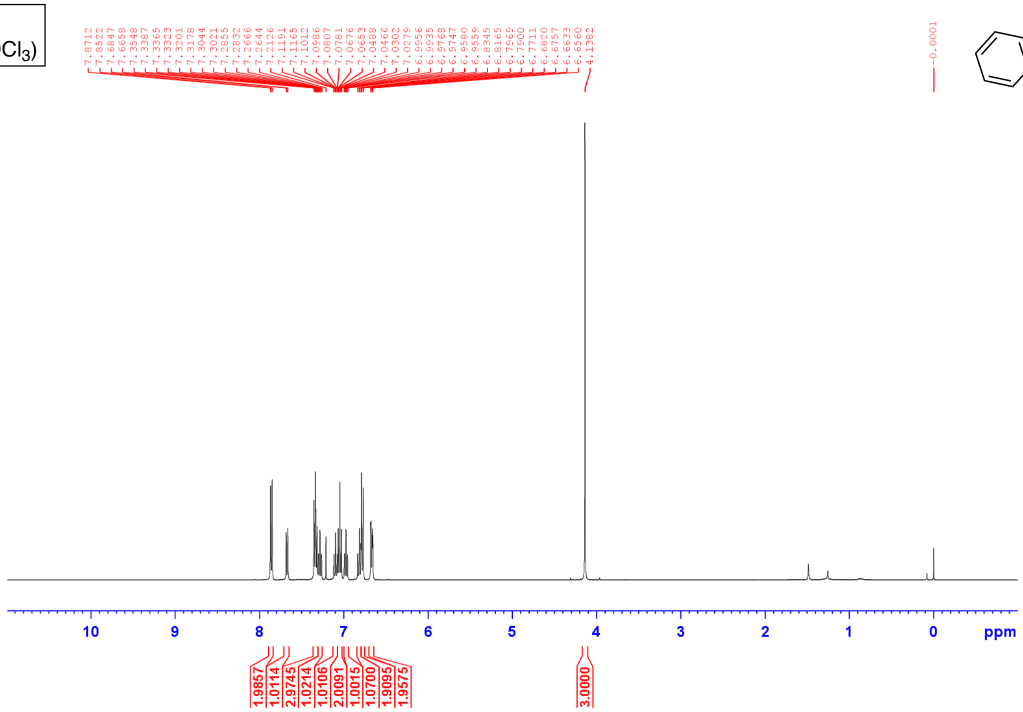

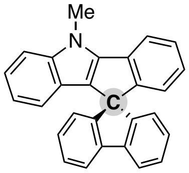

3aa

^{13}C{^{1}H} NMR
(100 MHz, $CDCl_3$)

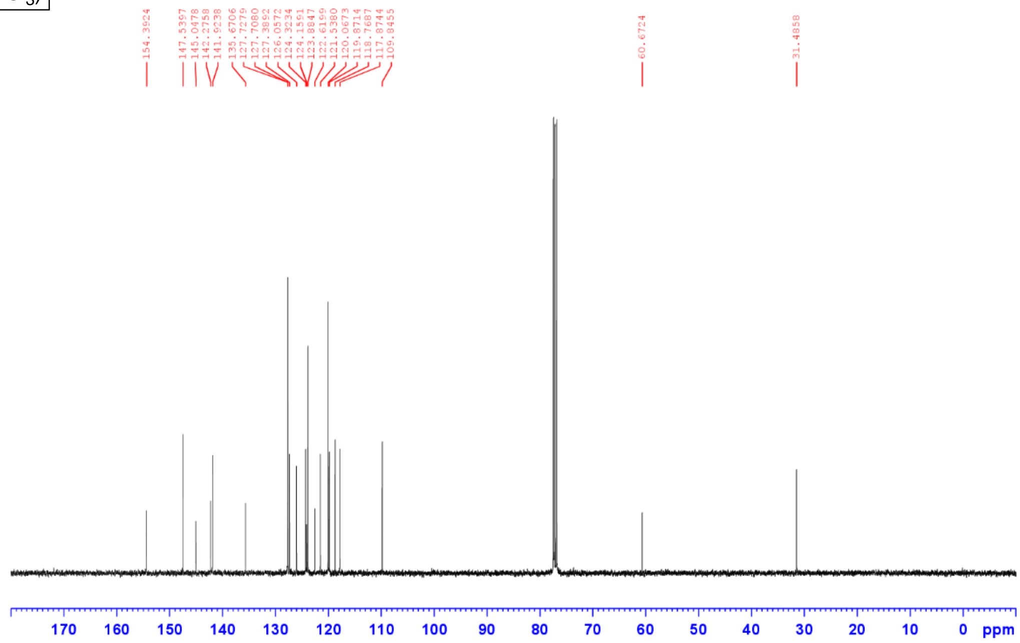

[^{1}H and ^{13}C{^{1}H} NMR Spectra of **3ba**]

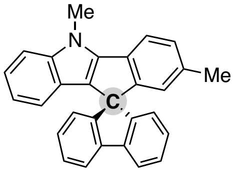

3ba

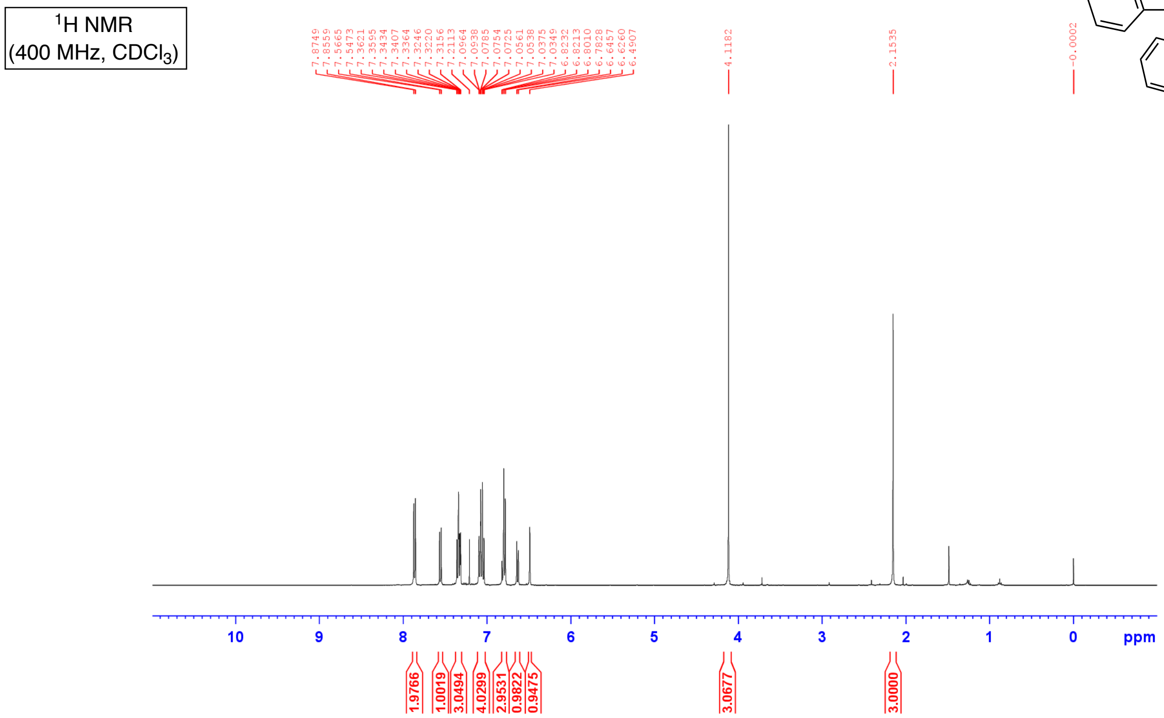

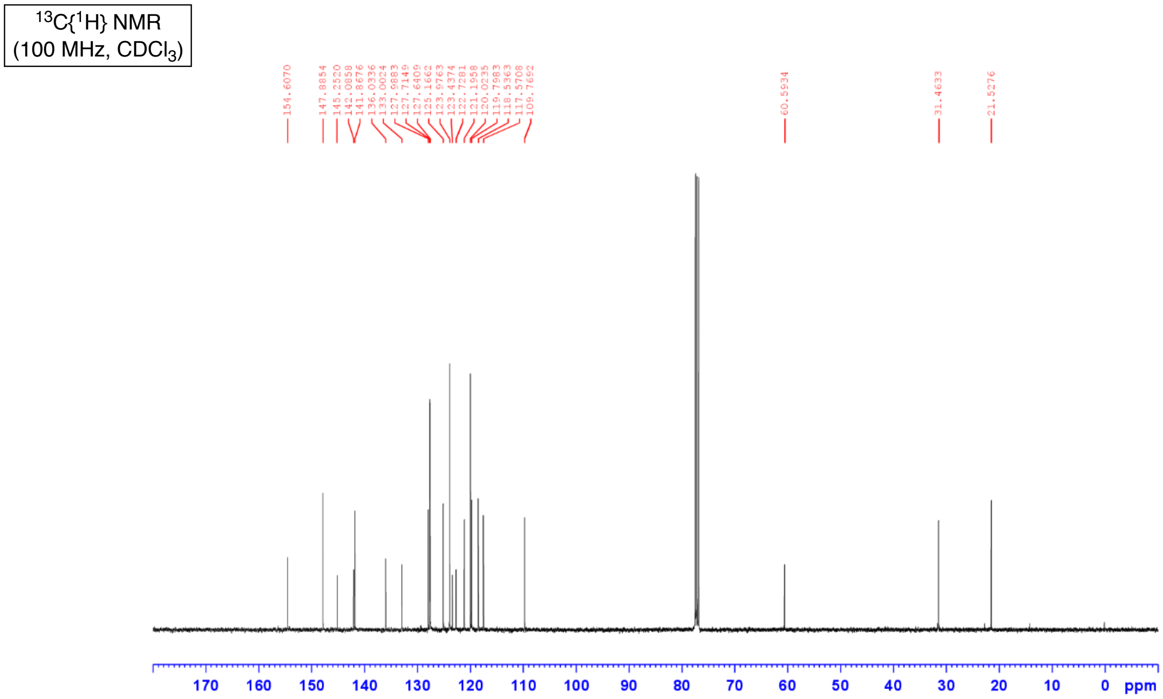

[^{1}H and ^{13}C{^{1}H} NMR Spectra of **3ca**]

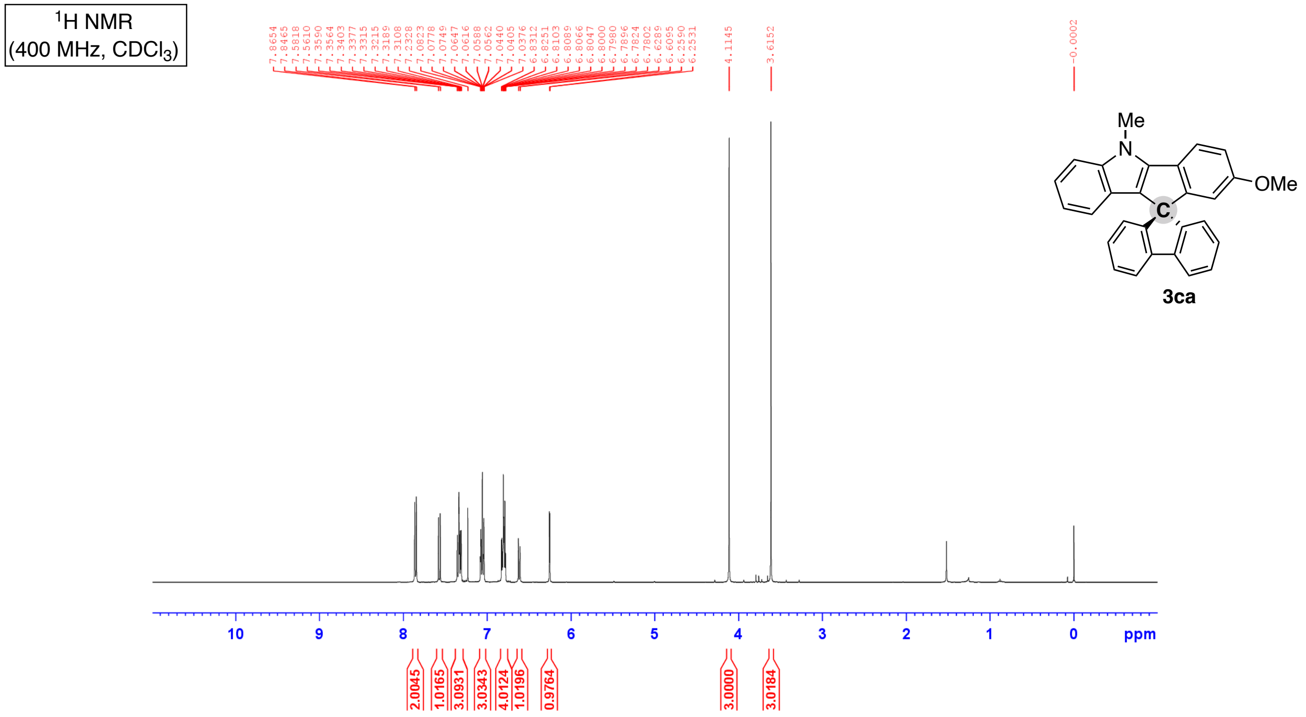

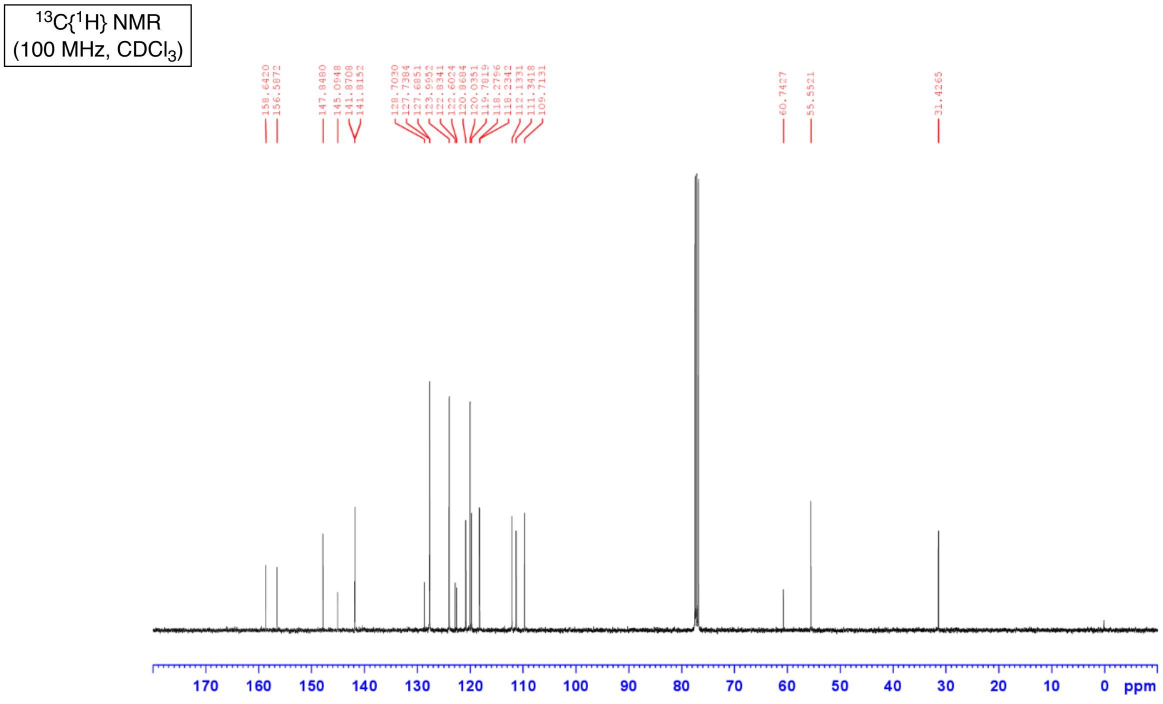

[^{1}H and ^{13}C{^{1}H} NMR Spectra of **3da**]

^{1}H NMR
(400 MHz, $CDCl_3$)

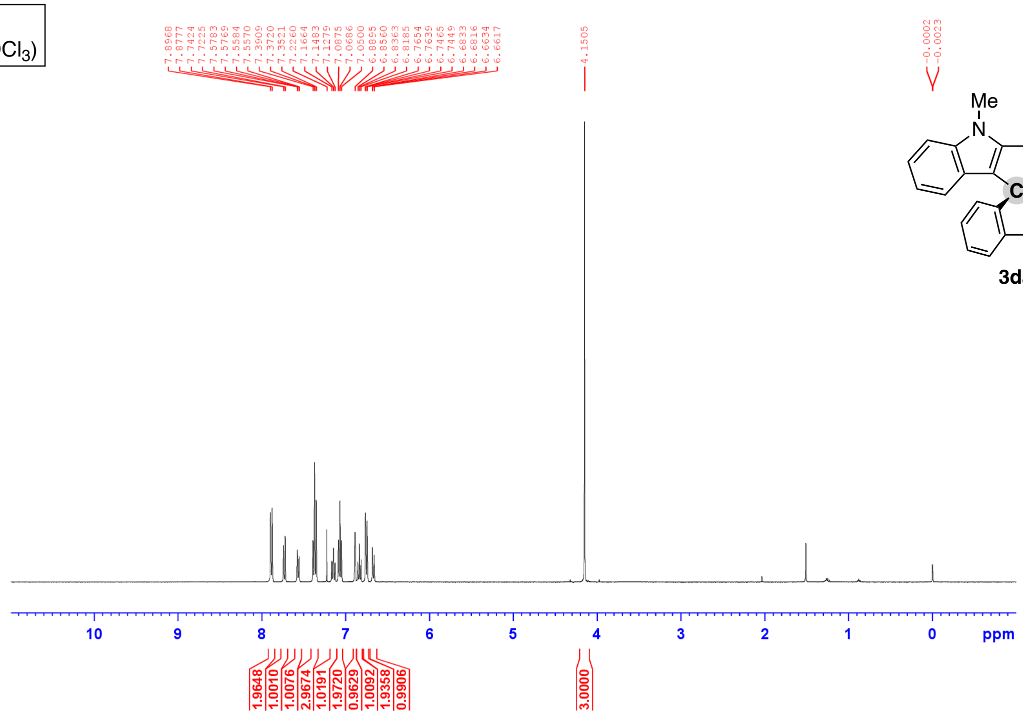

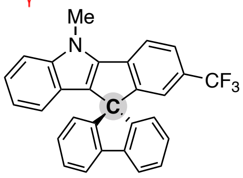

3da

^{13}C{^{1}H} NMR
(100 MHz, $CDCl_3$)

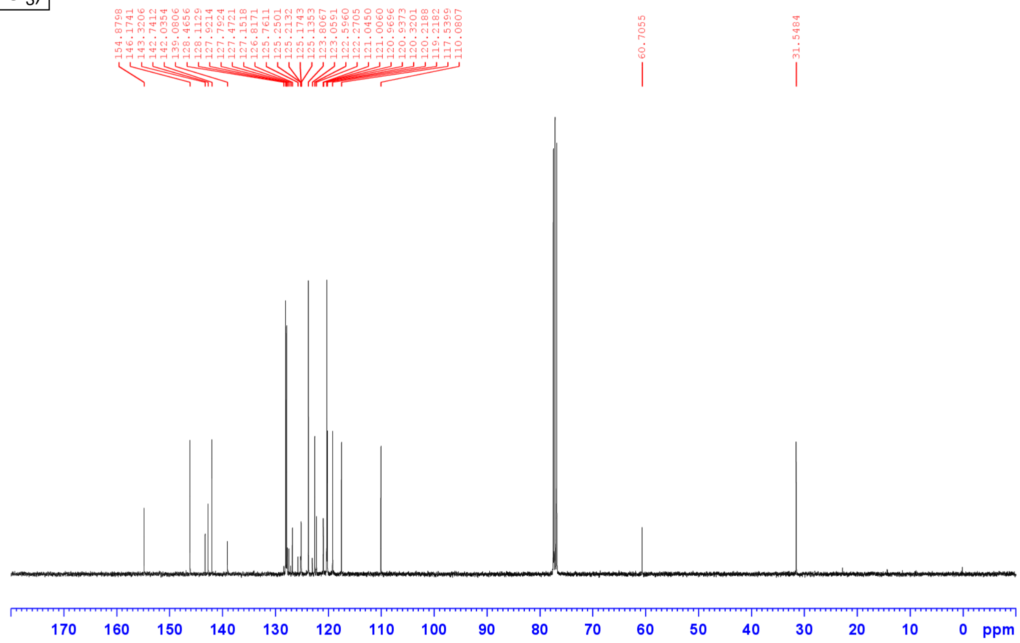

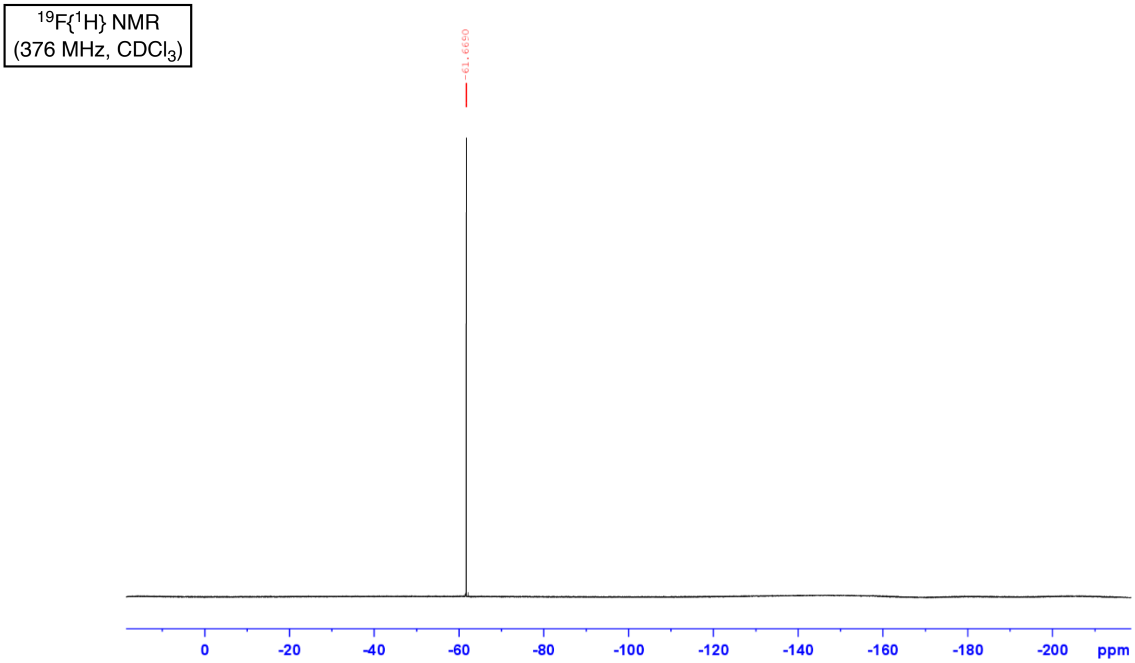
$^{19}F\{^1H\}$ NMR
(376 MHz, $CDCl_3$)
-61.6650
0
-20
-40
-60
-80
-100
-120
-140
-160
-180
-200
ppm

[^{1}H and ^{13}C{^{1}H} NMR Spectra of **3ea**]

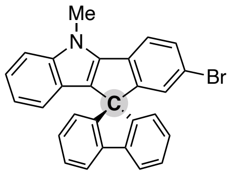

3ea

^{1}H NMR
(400 MHz, $CDCl_3$)

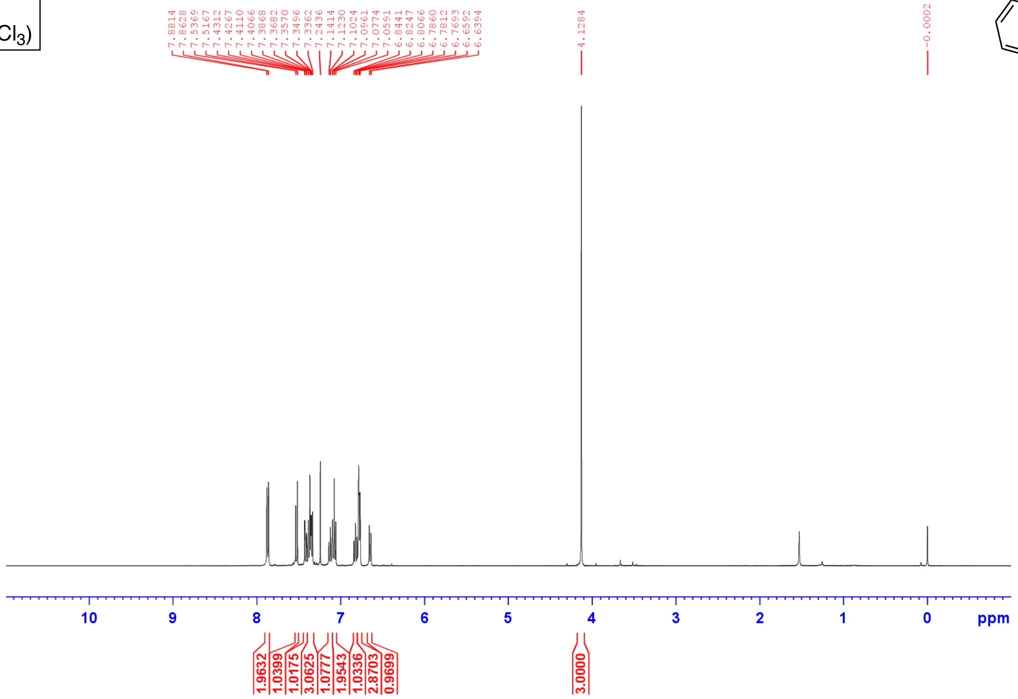

^{13}C{^{1}H} NMR
(100 MHz, $CDCl_3$)

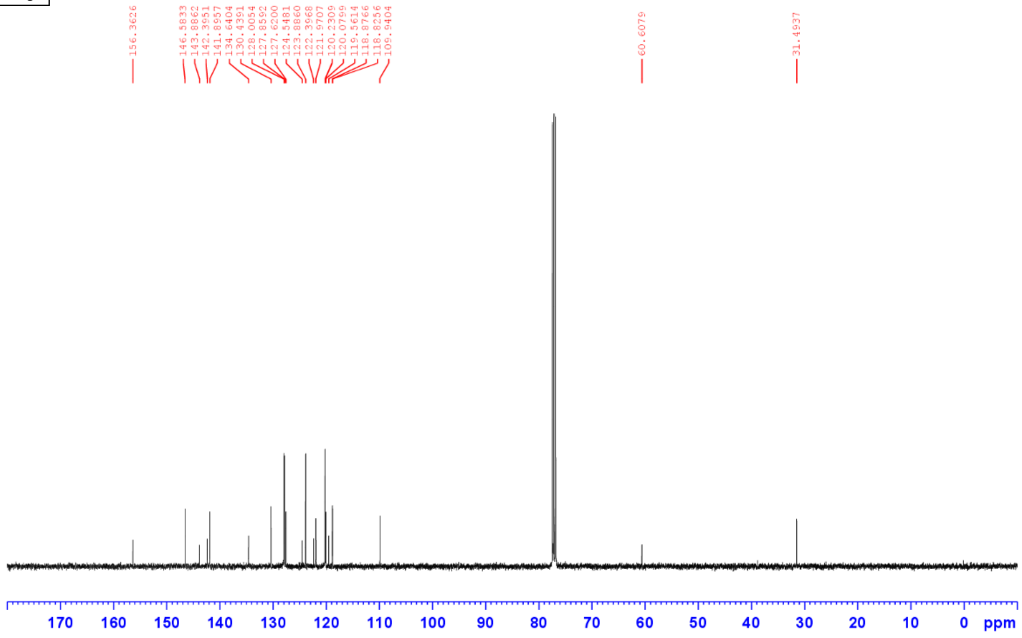

[^{1}H and ^{13}C{^{1}H} NMR Spectra of **3fa**]

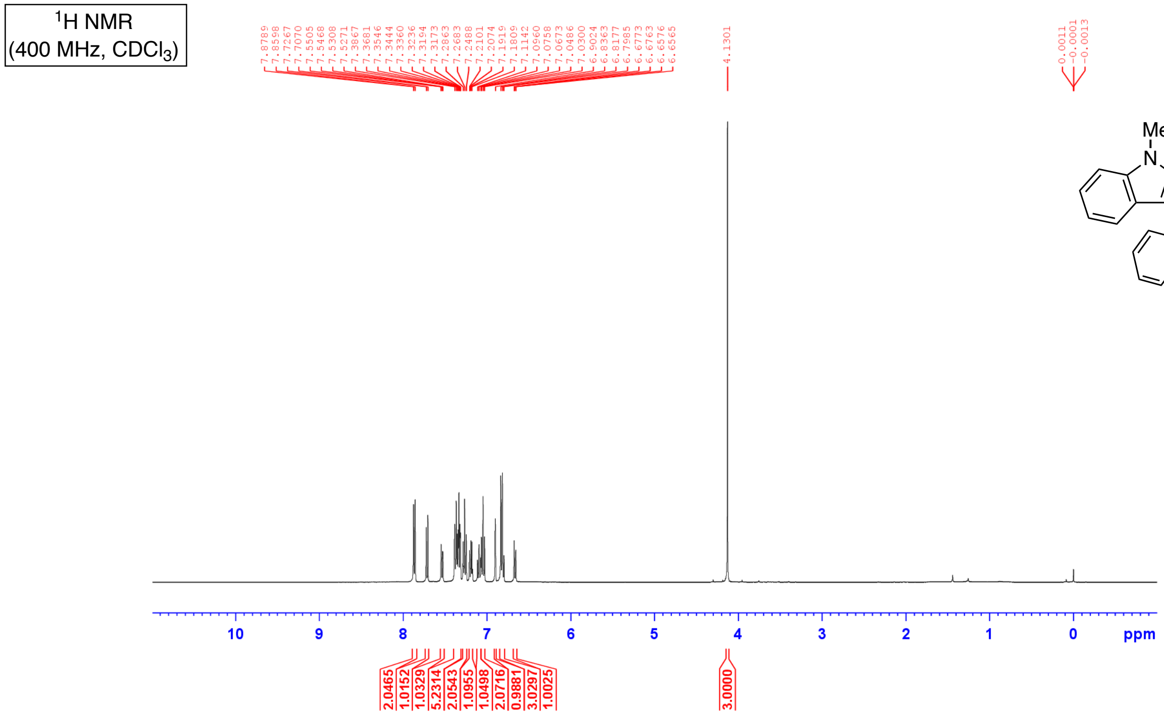

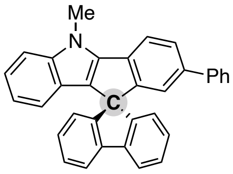

3fa

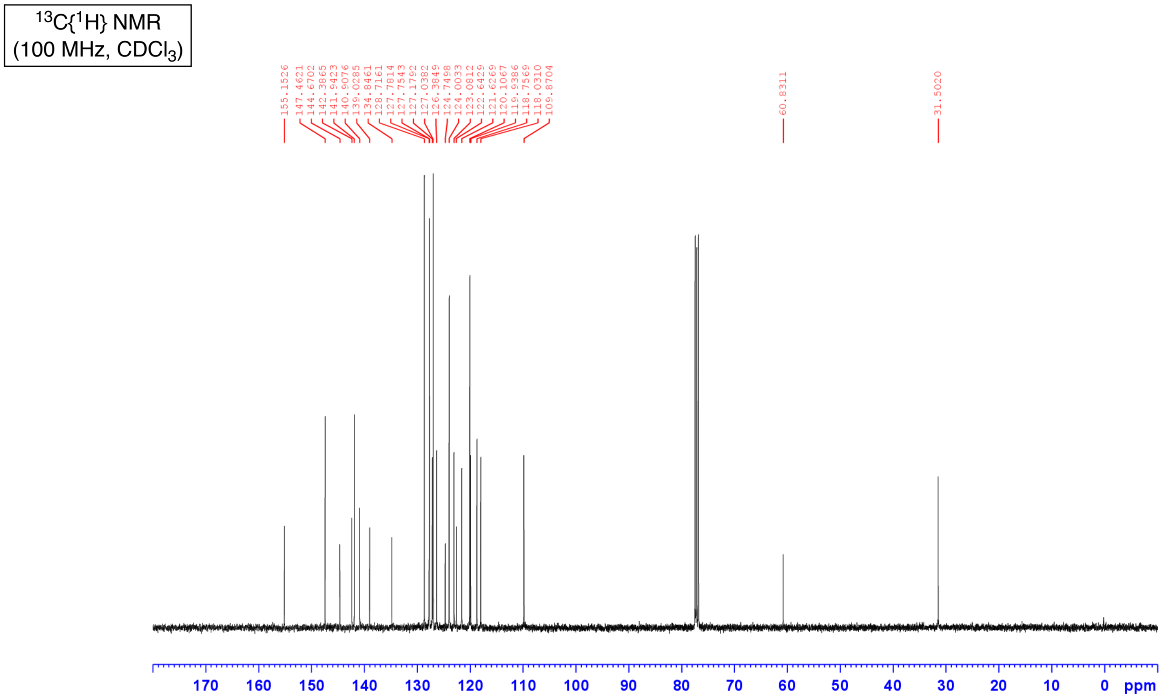

[^{1}H and ^{13}C{^{1}H} NMR Spectra of **3ga**]

^{1}H NMR
(400 MHz, $CDCl_3$)

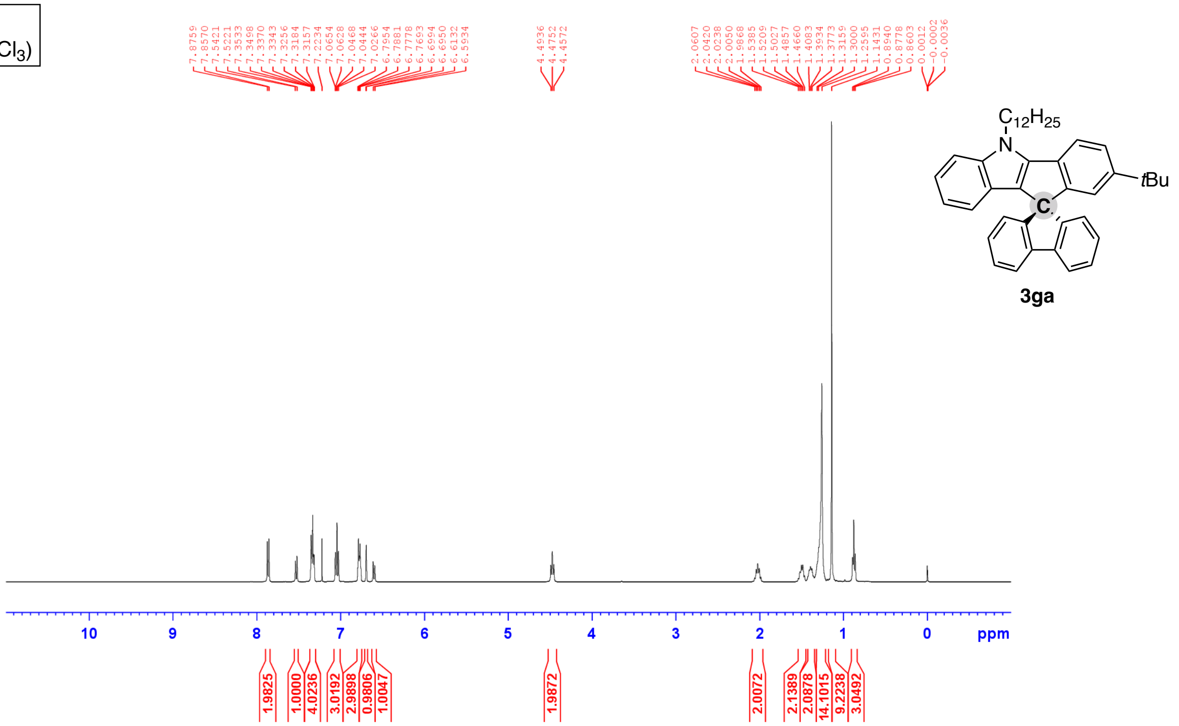

^{13}C{^{1}H} NMR
(100 MHz, $CDCl_3$)

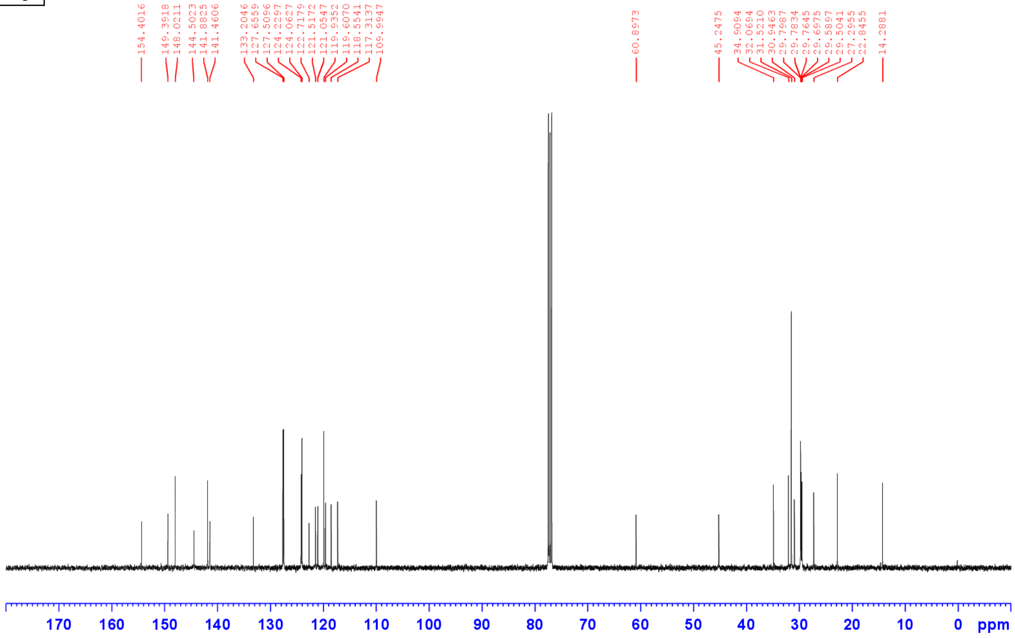

[^{1}H and $^{13}C\{^{1}H\}$ NMR Spectra of **3ha**]

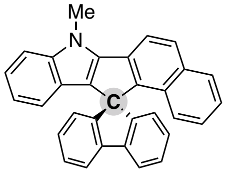

3ha

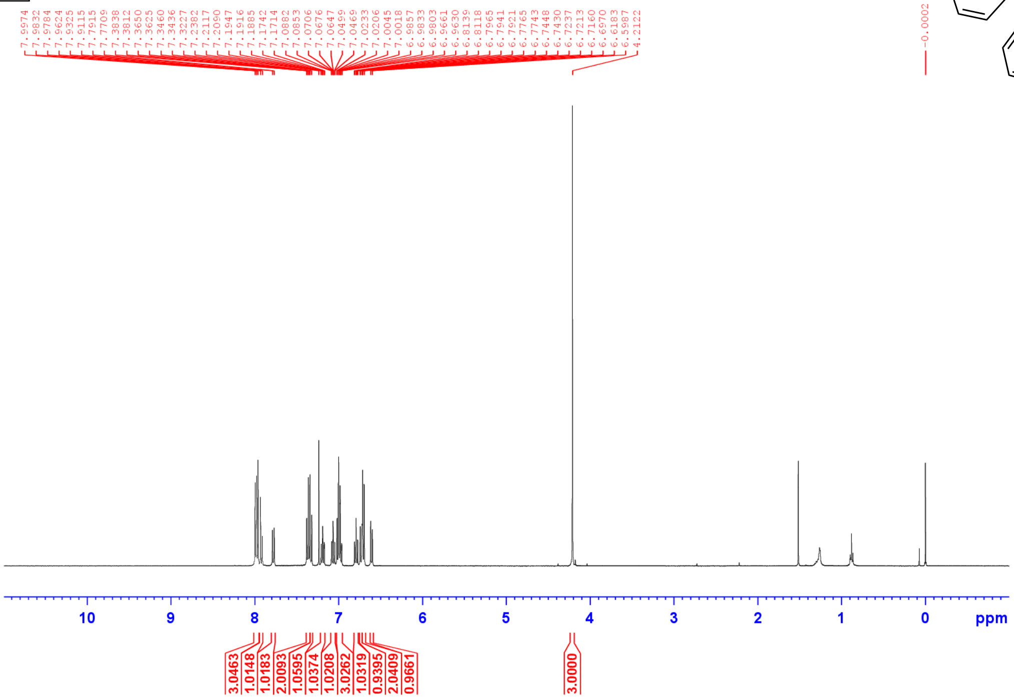

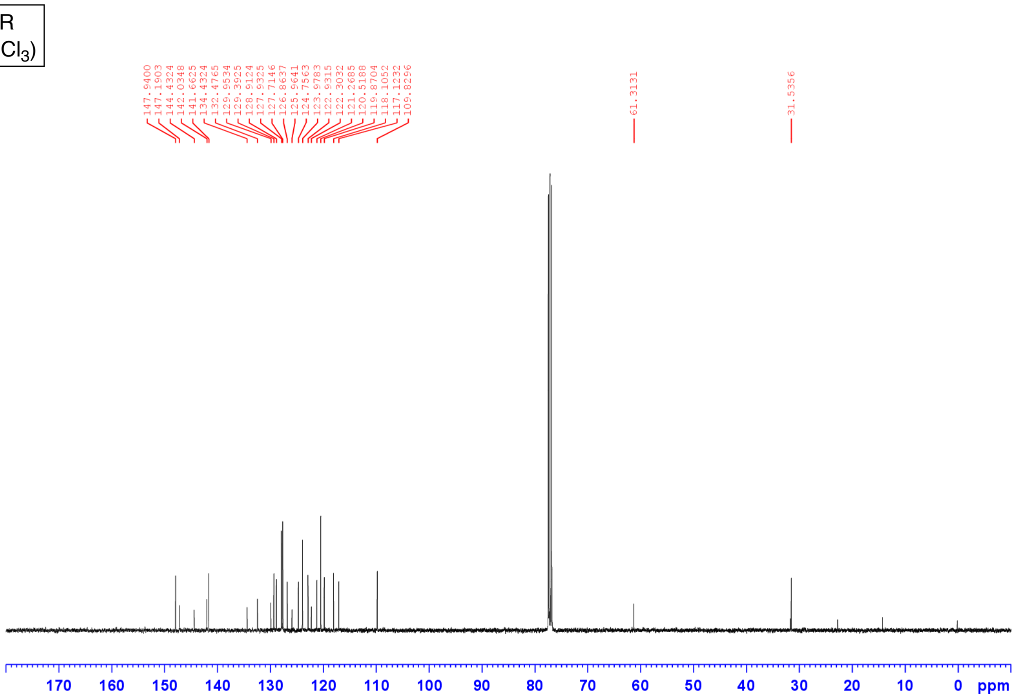

[^{1}H and ^{13}C{^{1}H} NMR Spectra of **3ia'**]

^{1}H NMR
(400 MHz, $CDCl_3$)

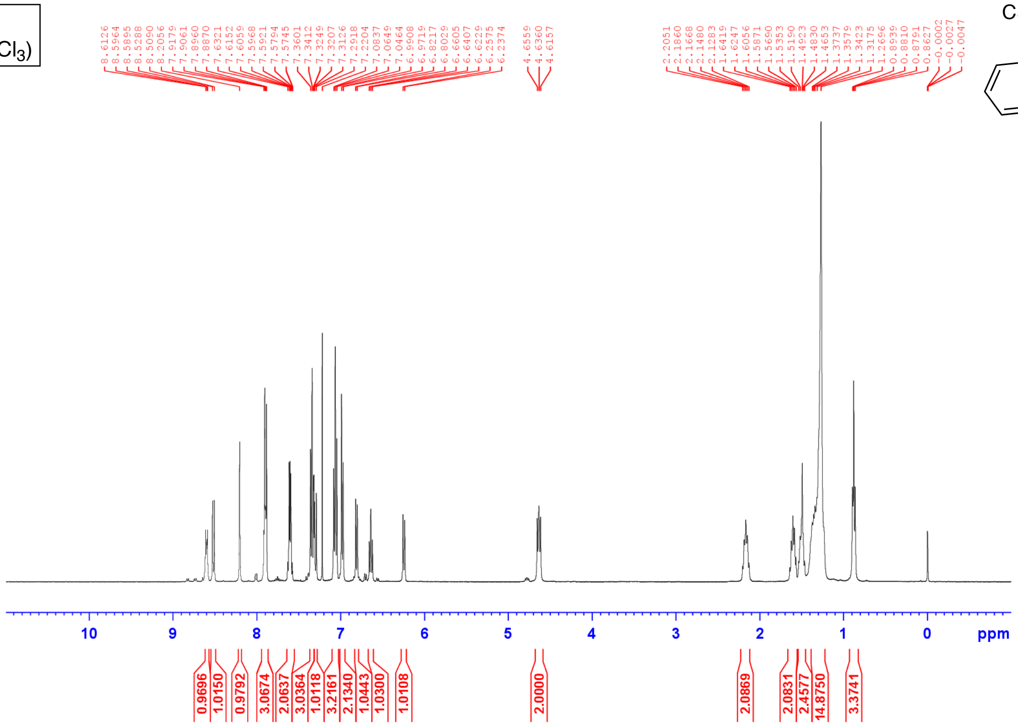

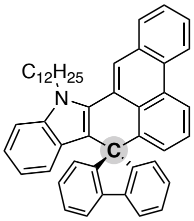

3ia'

^{13}C{^{1}H} NMR
(100 MHz, $CDCl_3$)

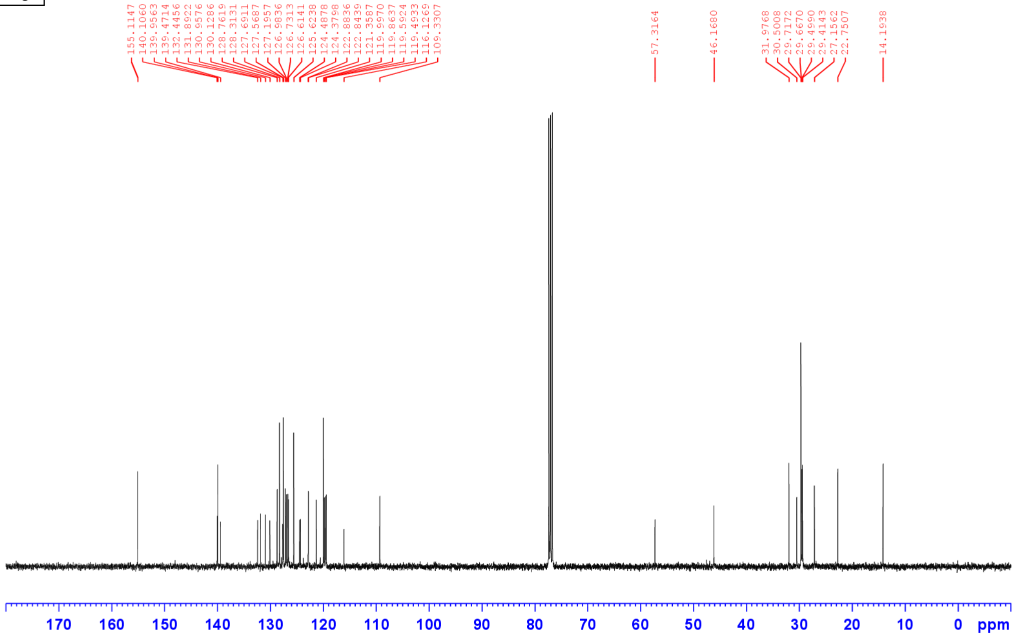

[^{1}H and ^{13}C{^{1}H} NMR Spectra of **3ja**]

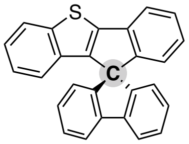

3ja

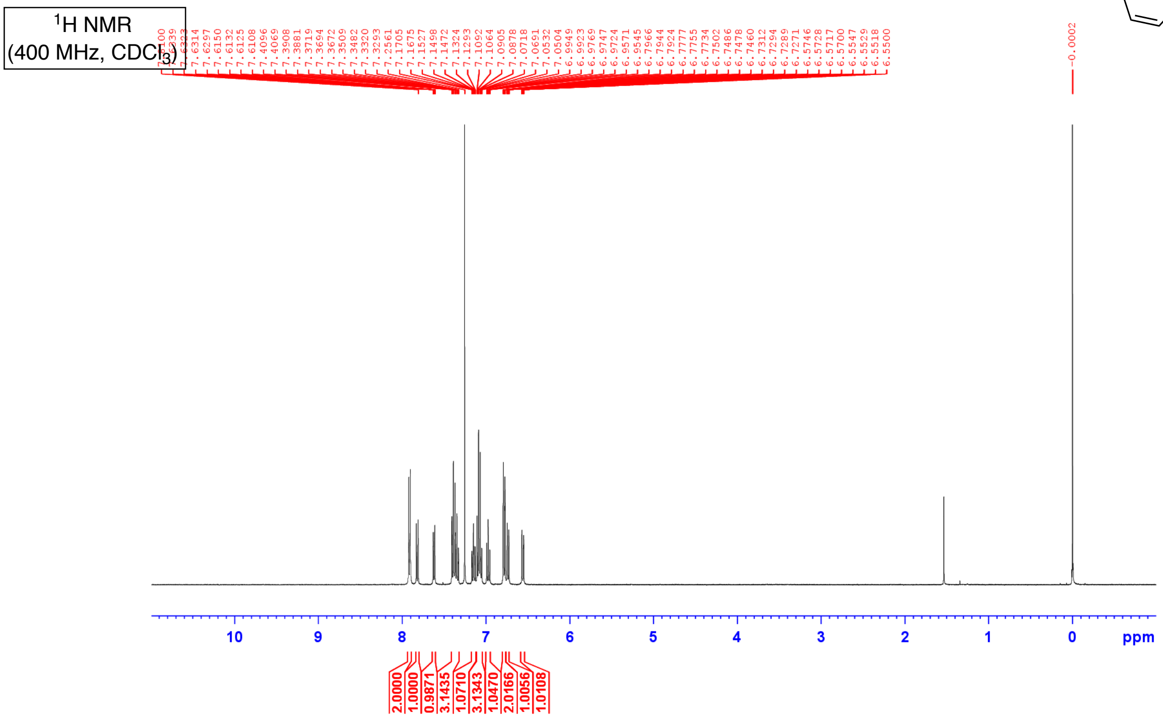

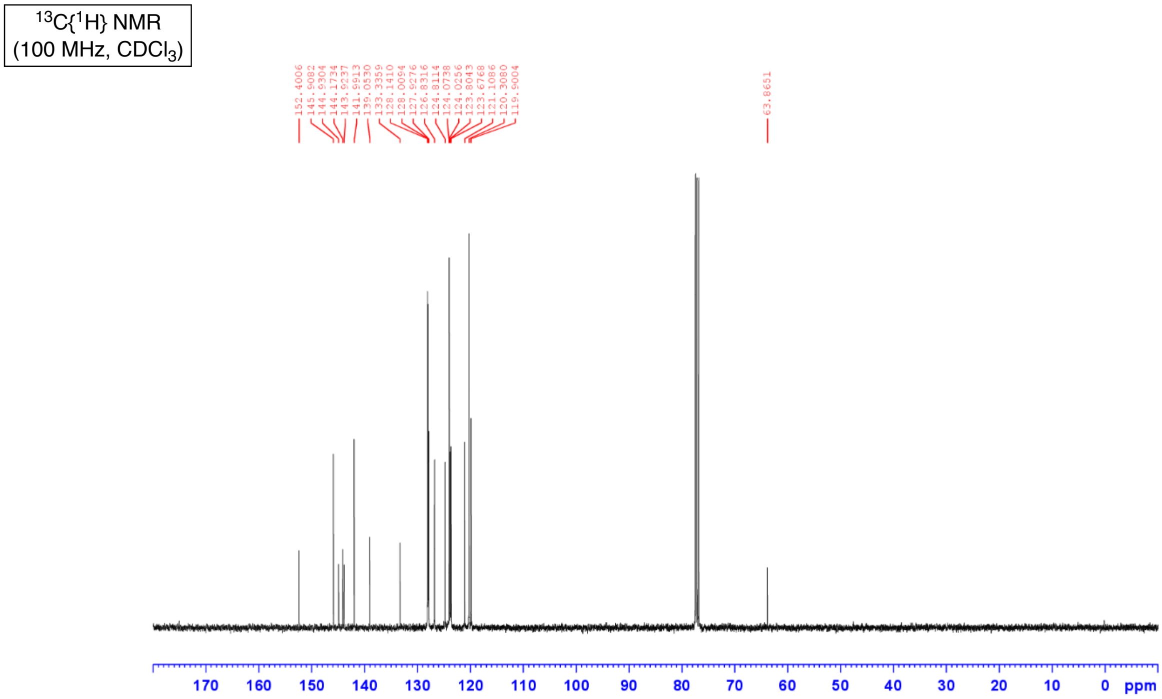

[^{1}H and ^{13}C{^{1}H} NMR Spectra of **3ka**]

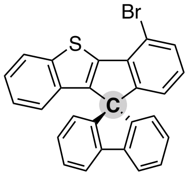

3ka

^{1}H NMR
(400 MHz, $CDCl_3$)

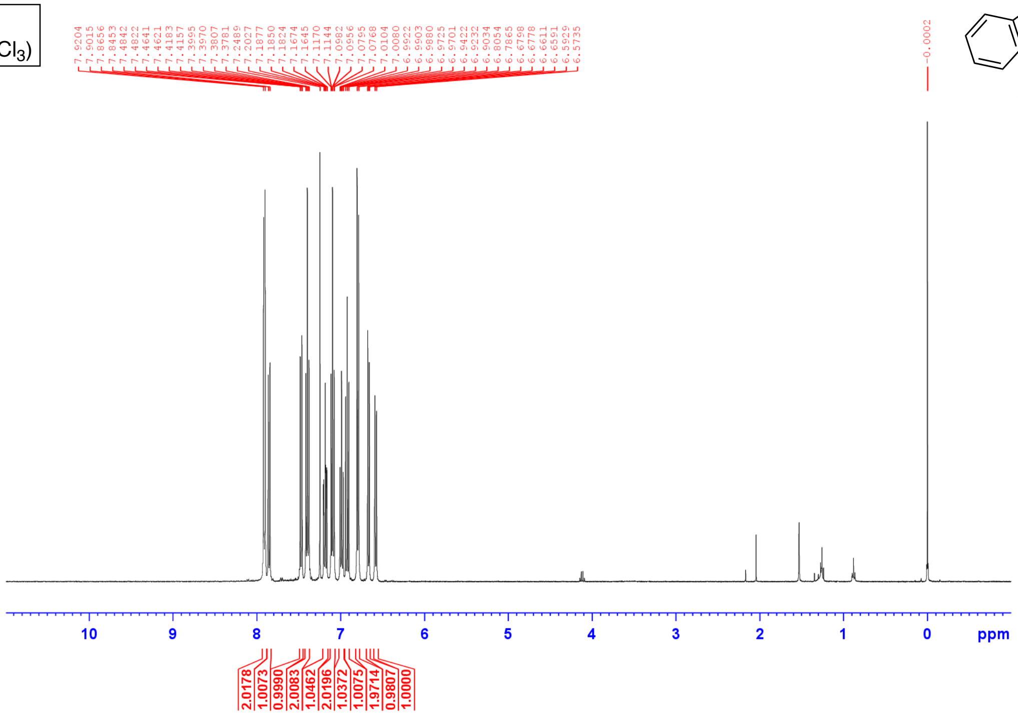

^{13}C{^{1}H} NMR
(100 MHz, $CDCl_3$)

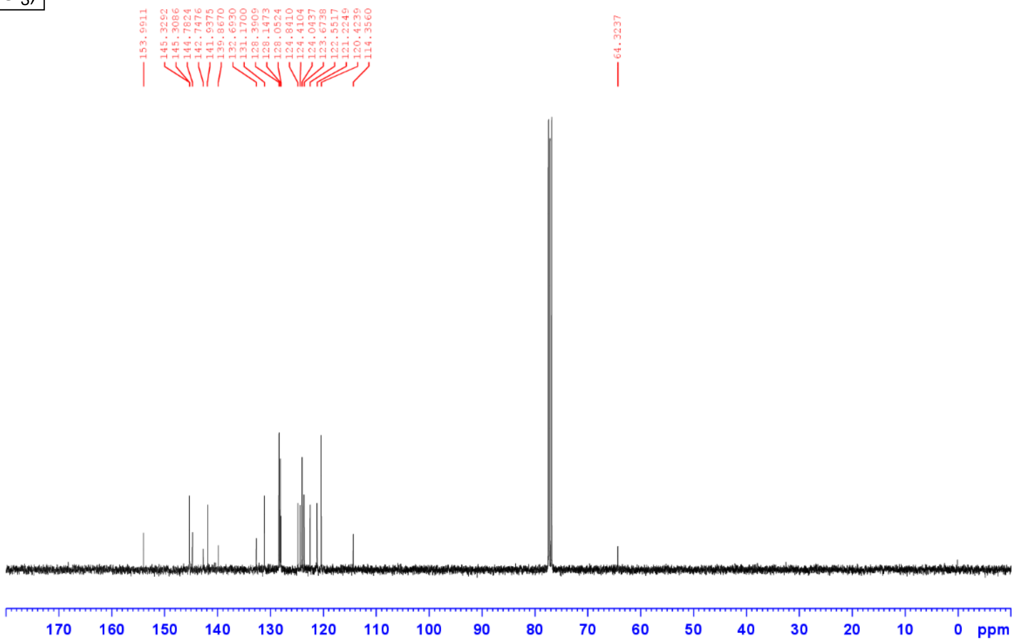

[^{1}H and ^{13}C{^{1}H} NMR Spectra of **3la**]

^{1}H NMR
(400 MHz, $CDCl_3$)

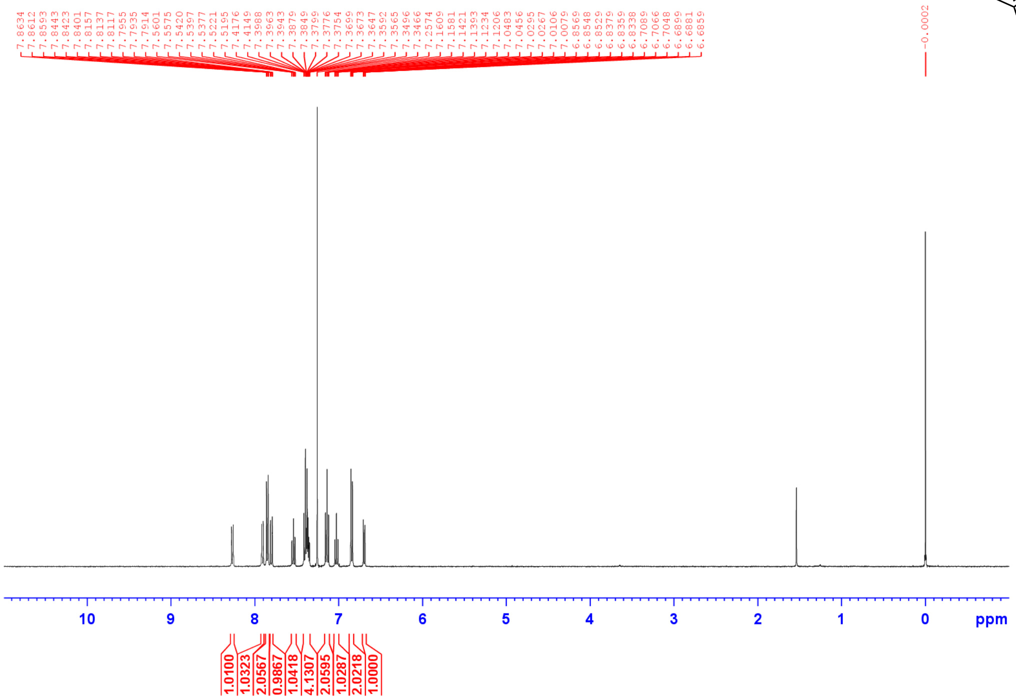

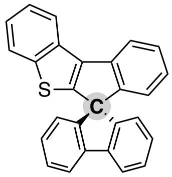

3la

^{13}C{^{1}H} NMR
(100 MHz, $CDCl_3$)

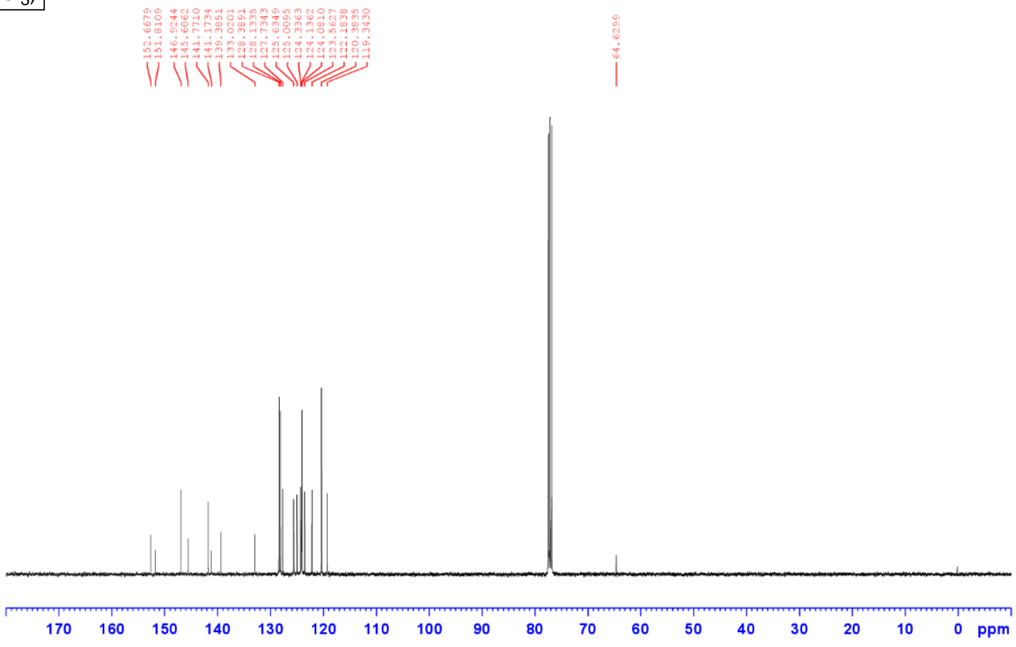

[^{1}H and $^{13}C\{^{1}H\}$ NMR Spectra of **3la’**]

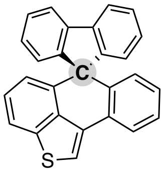

3la’

^{1}H NMR
(400 MHz, $CDCl_3$)

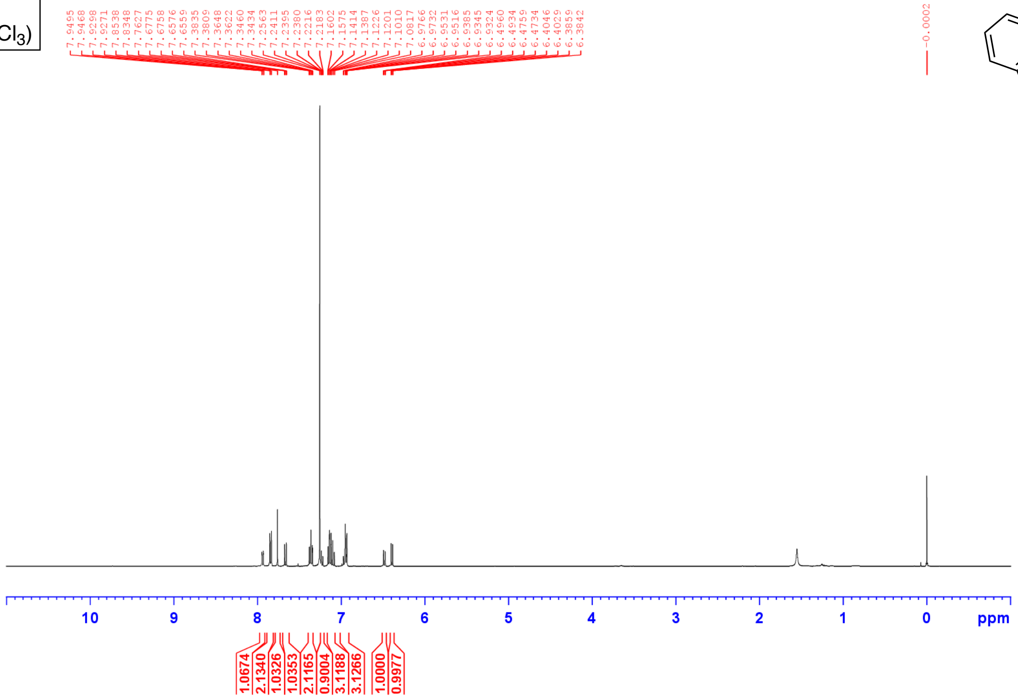

$^{13}C\{^{1}H\}$ NMR
(100 MHz, $CDCl_3$)

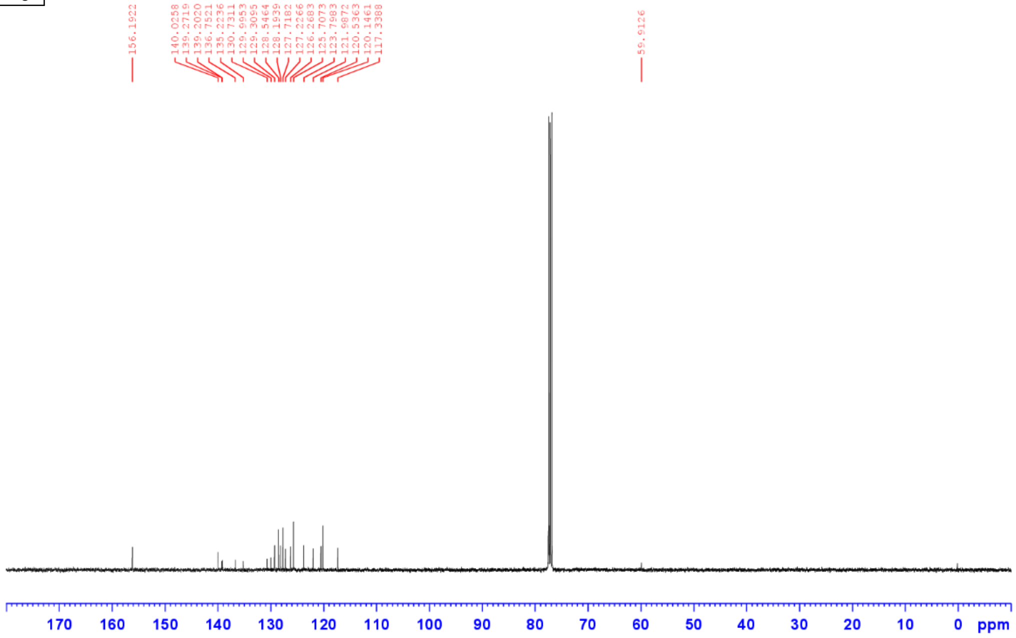

[^{1}H and ^{13}C{^{1}H} NMR Spectra of **3ma**]

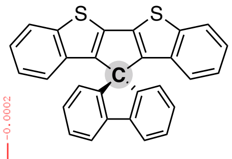

3ma

^{1}H NMR
(400 MHz, $CDCl_3$)

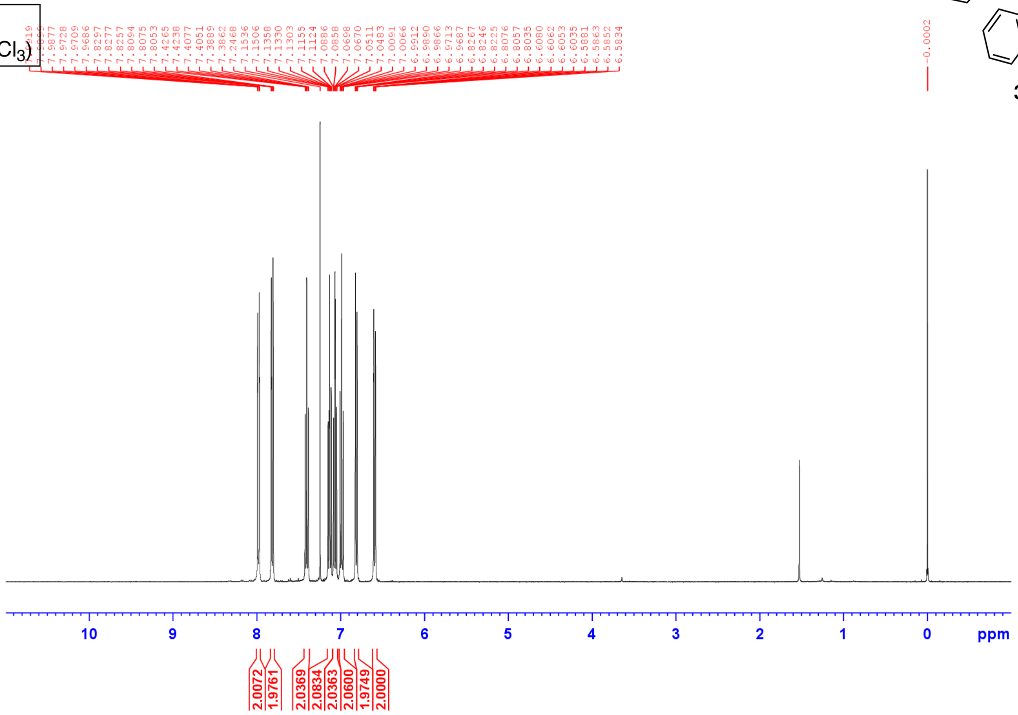

^{13}C{^{1}H} NMR
(100 MHz, $CDCl_3$)

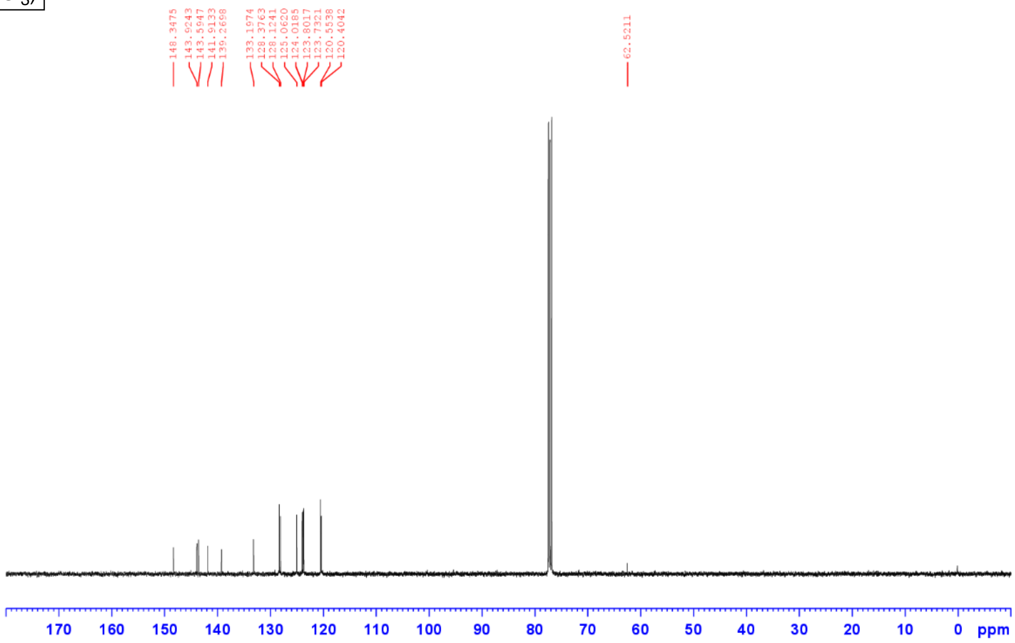

[^{1}H and ^{13}C{^{1}H} NMR Spectra of **3na**]

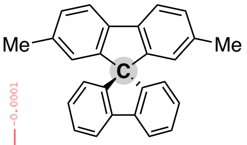

3na

^{1}H NMR
(400 MHz, $CDCl_3$)

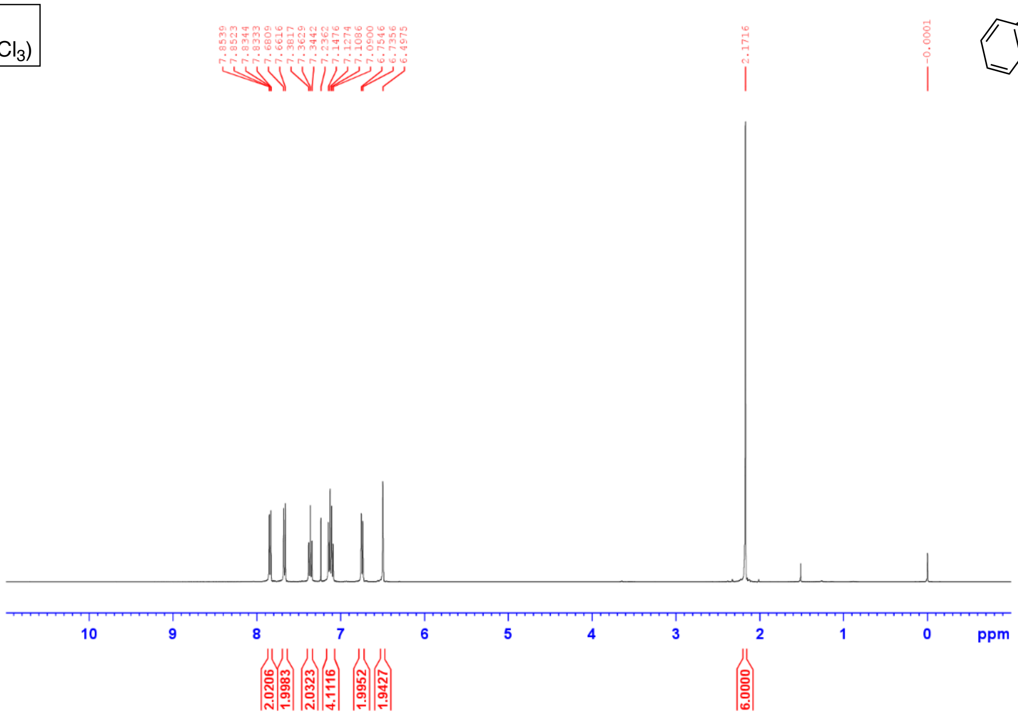

^{13}C{^{1}H} NMR
(100 MHz, $CDCl_3$)

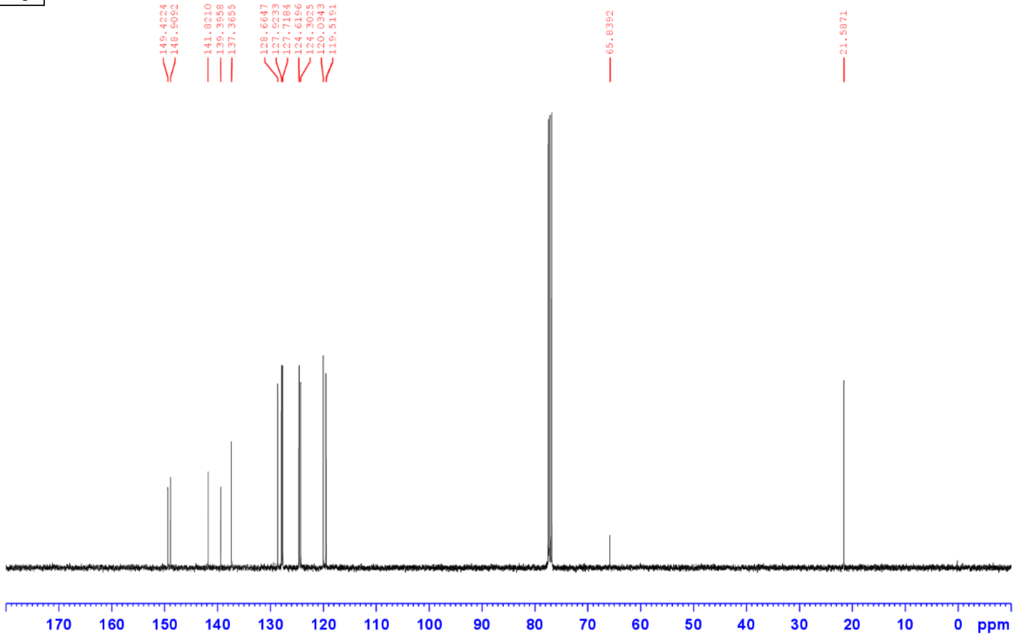

[^{1}H and ^{13}C{^{1}H} NMR Spectra of **3oa**]

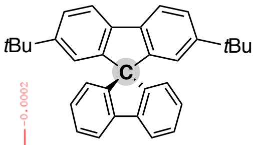

3oa

^{1}H NMR
(400 MHz, $CDCl_3$)

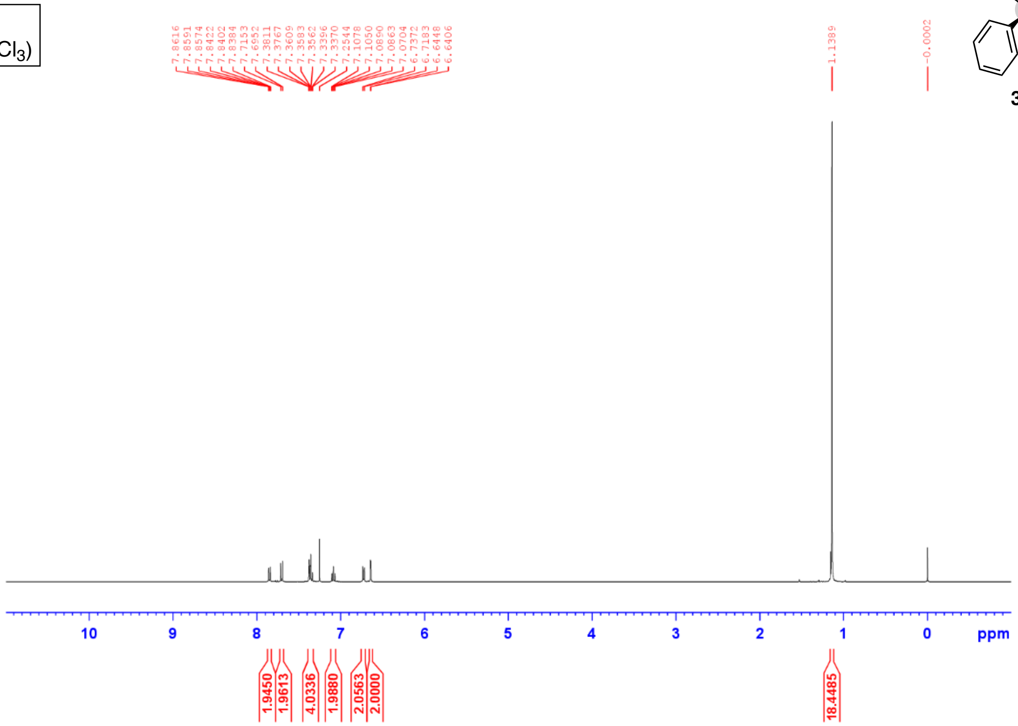

^{13}C{^{1}H} NMR
(100 MHz, $CDCl_3$)

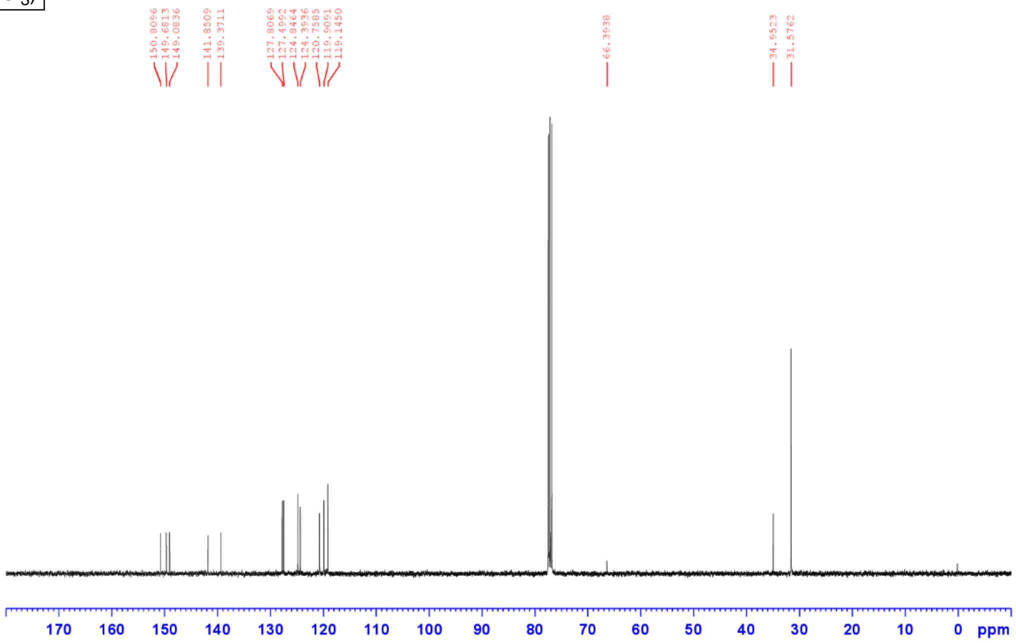

[^{1}H and ^{13}C{^{1}H} NMR Spectra of **3pa**]

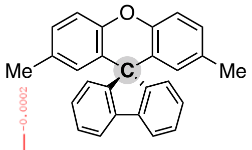

3pa

^{1}H NMR
(400 MHz, $CDCl_3$)

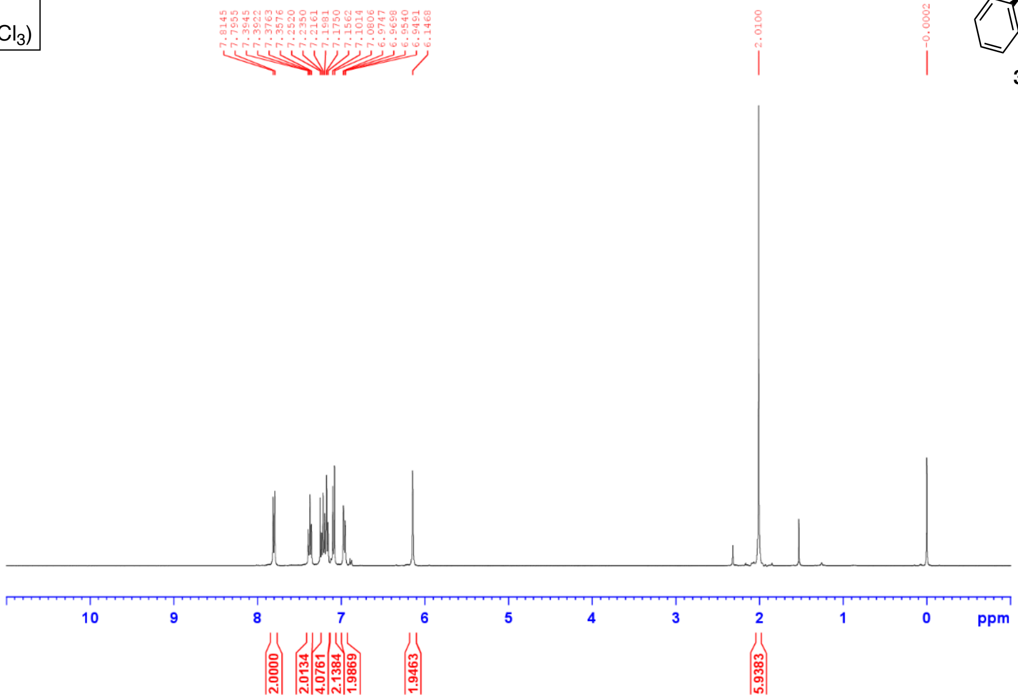

^{13}C{^{1}H} NMR
(100 MHz, $CDCl_3$)

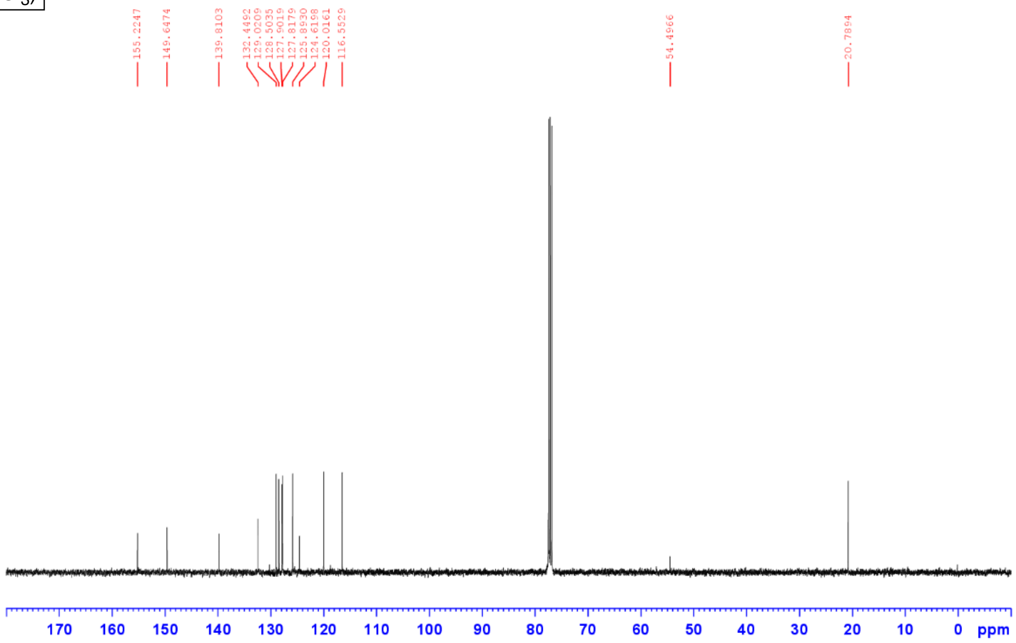

[^{1}H and ^{13}C{^{1}H} NMR Spectra of **3ab**]

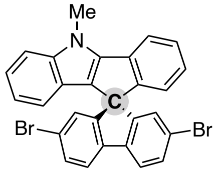

3ab

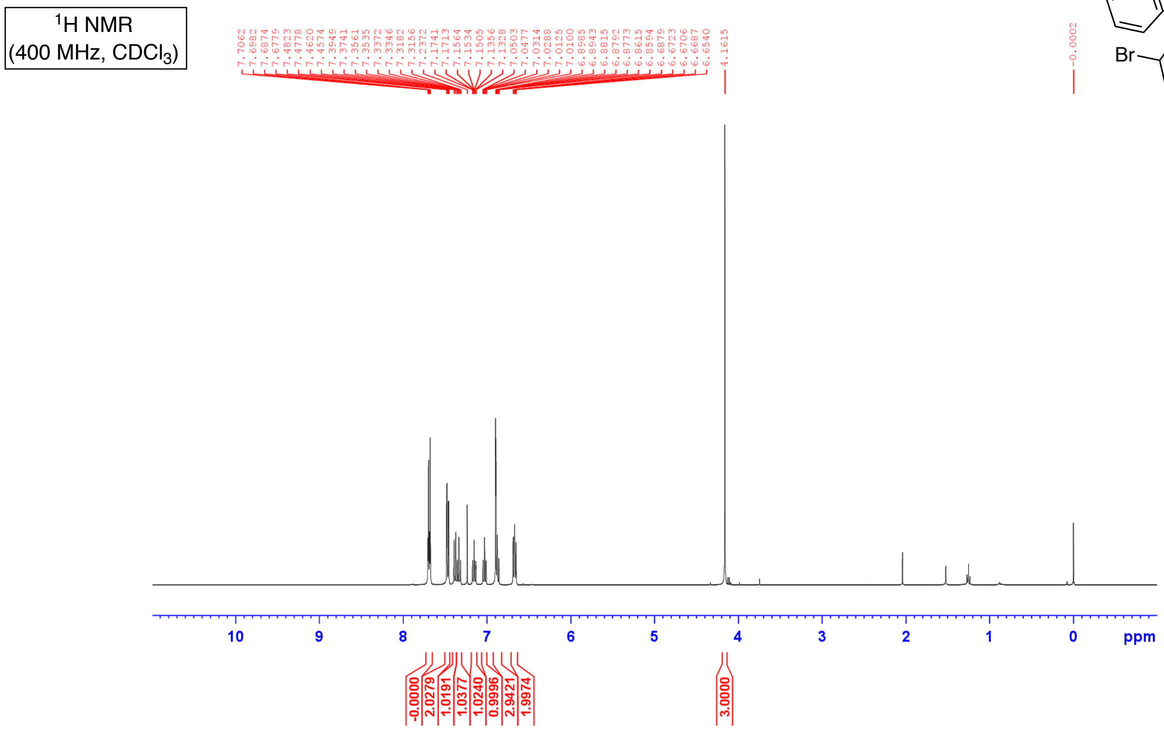

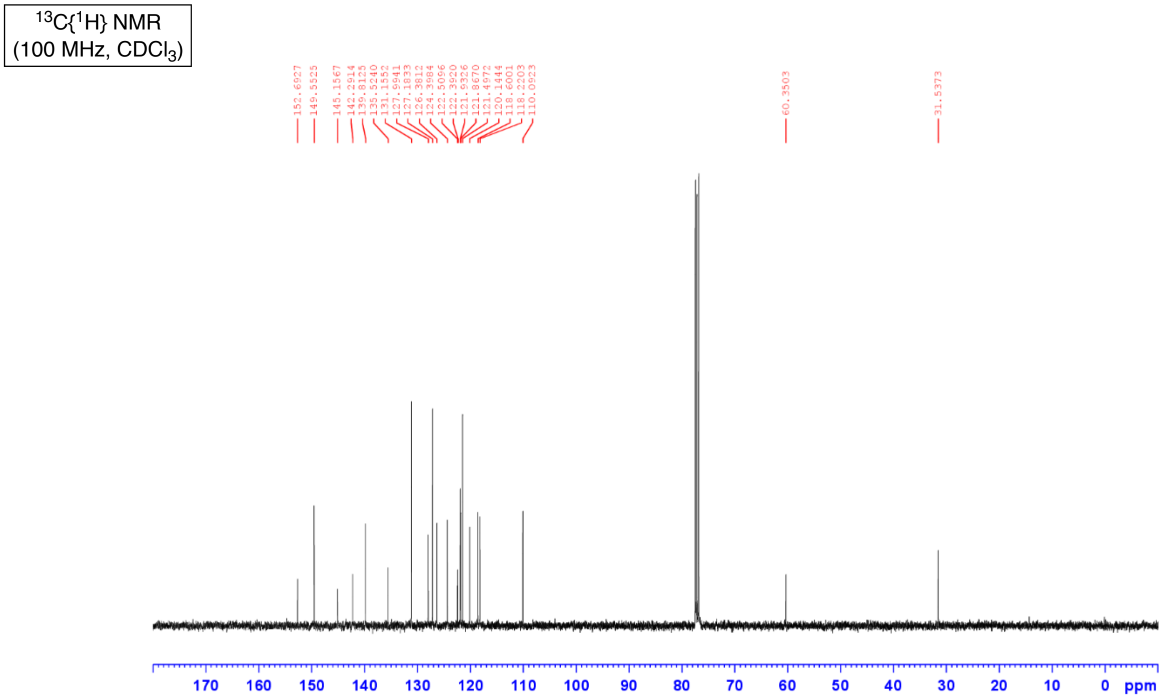

[^{1}H and ^{13}C{^{1}H} NMR Spectra of **3ac**]

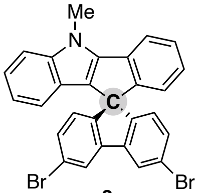

3ac

^{1}H NMR
(400 MHz, $CDCl_3$)

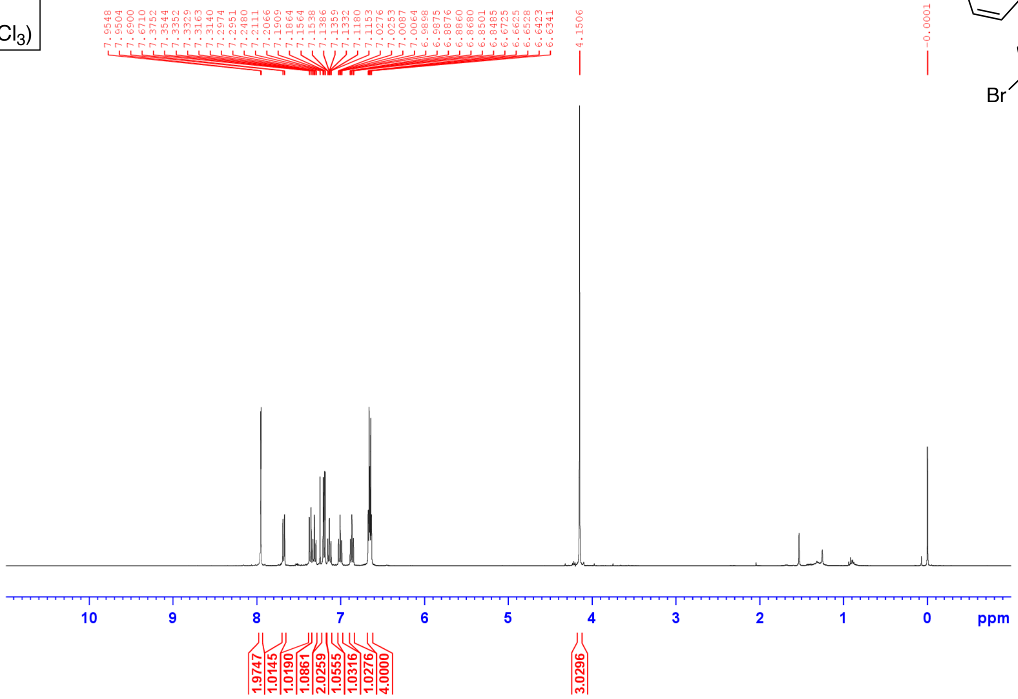

^{13}C{^{1}H} NMR
(100 MHz, $CDCl_3$)

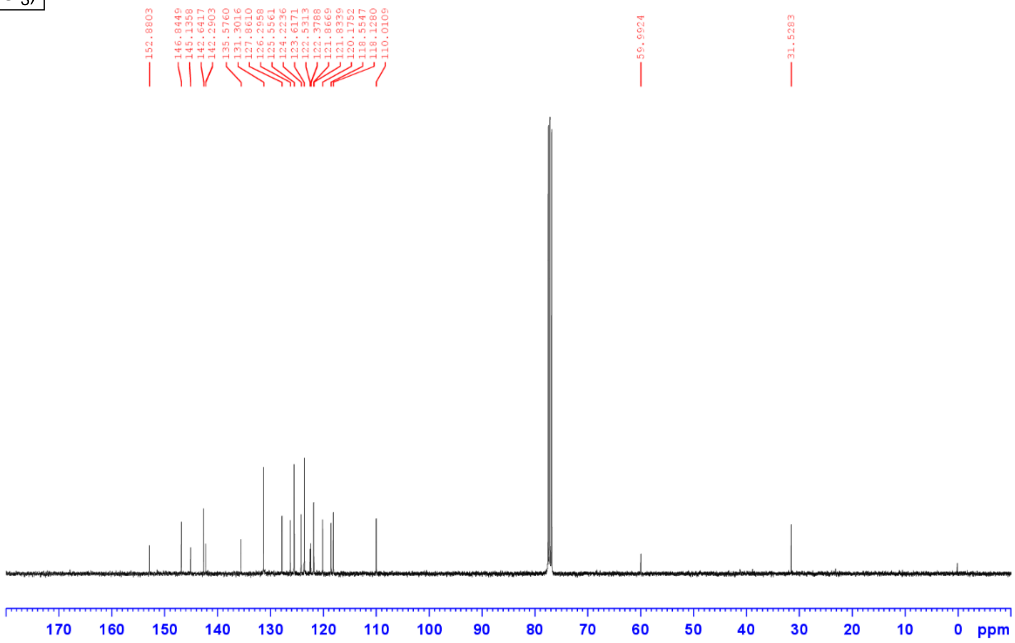

[^{1}H and ^{13}C{^{1}H} NMR Spectra of **3ad**]

^{1}H NMR
(400 MHz, $CDCl_3$)

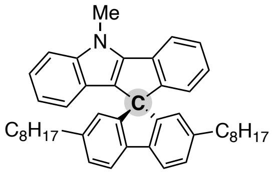

3ad

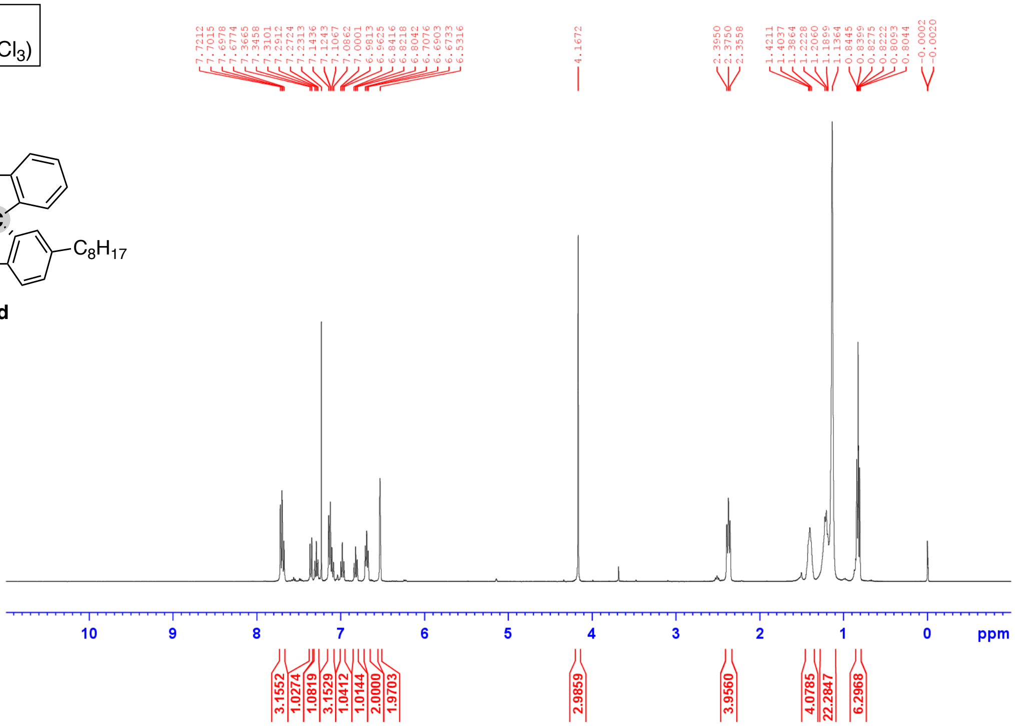

^{13}C{^{1}H} NMR
(100 MHz, $CDCl_3$)

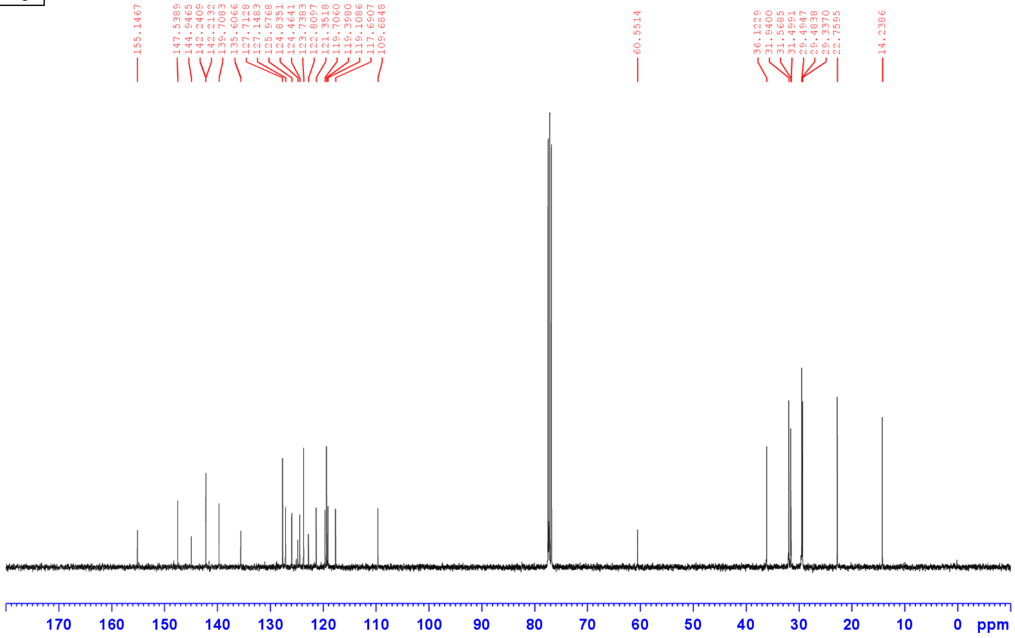

[^{1}H and ^{13}C{^{1}H} NMR Spectra of **3ae**]

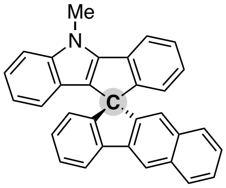

3ae

^{1}H NMR
(400 MHz, $CDCl_3$)

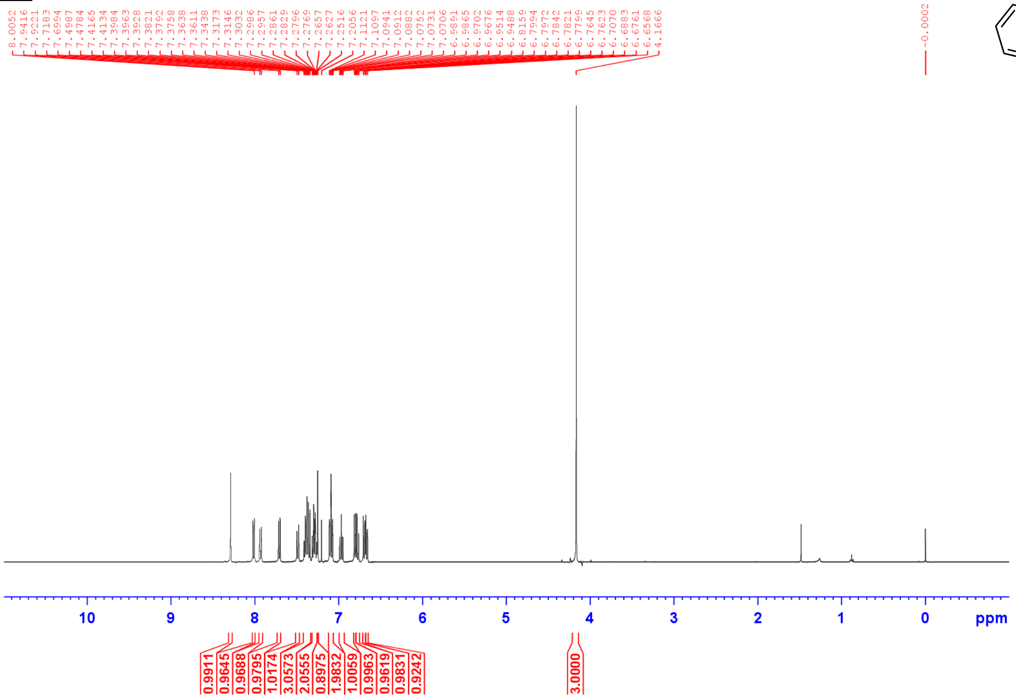

^{13}C{^{1}H} NMR
(100 MHz, $CDCl_3$)

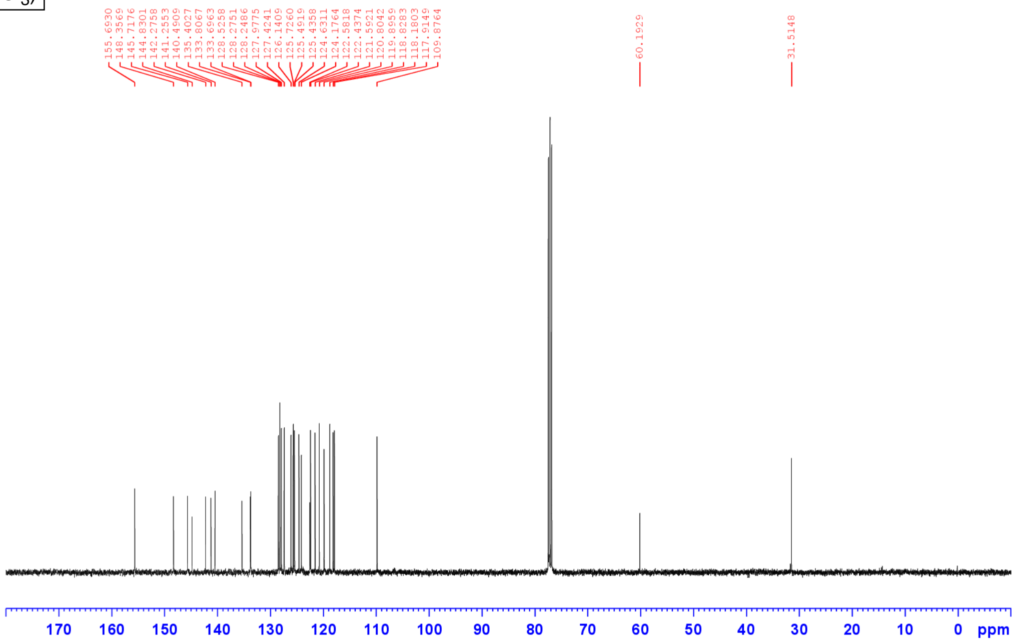

[^{1}H and ^{13}C{^{1}H} NMR Spectra of **3af**]

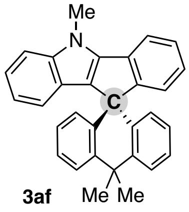

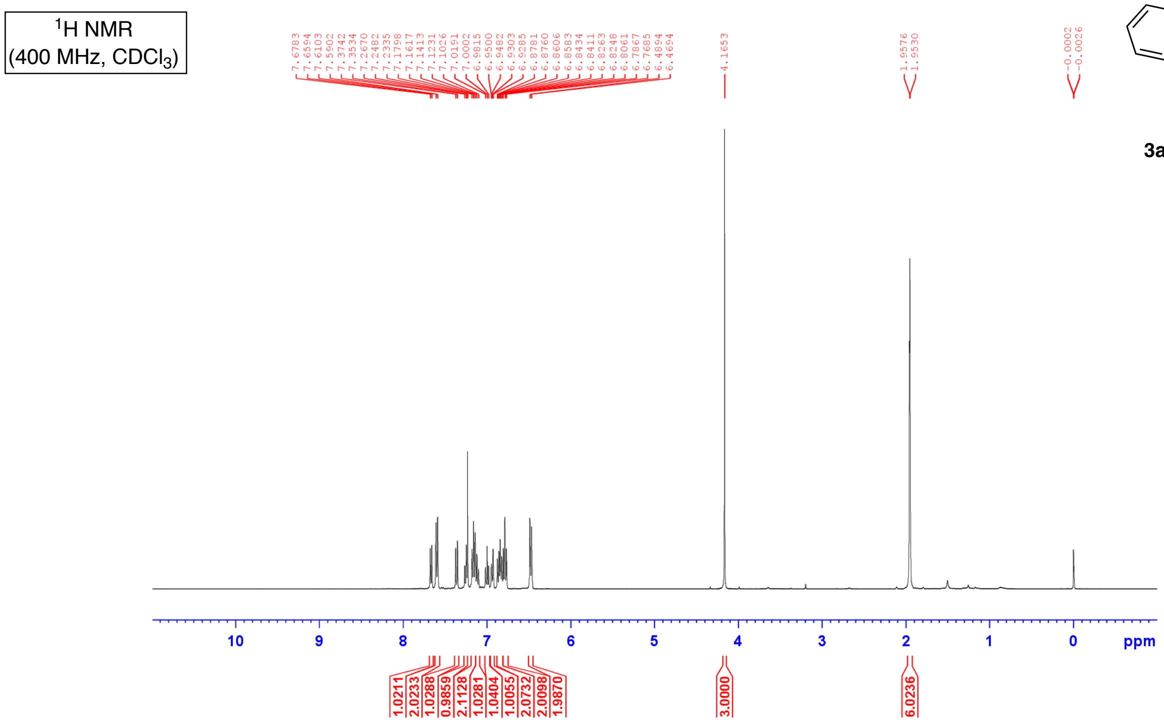

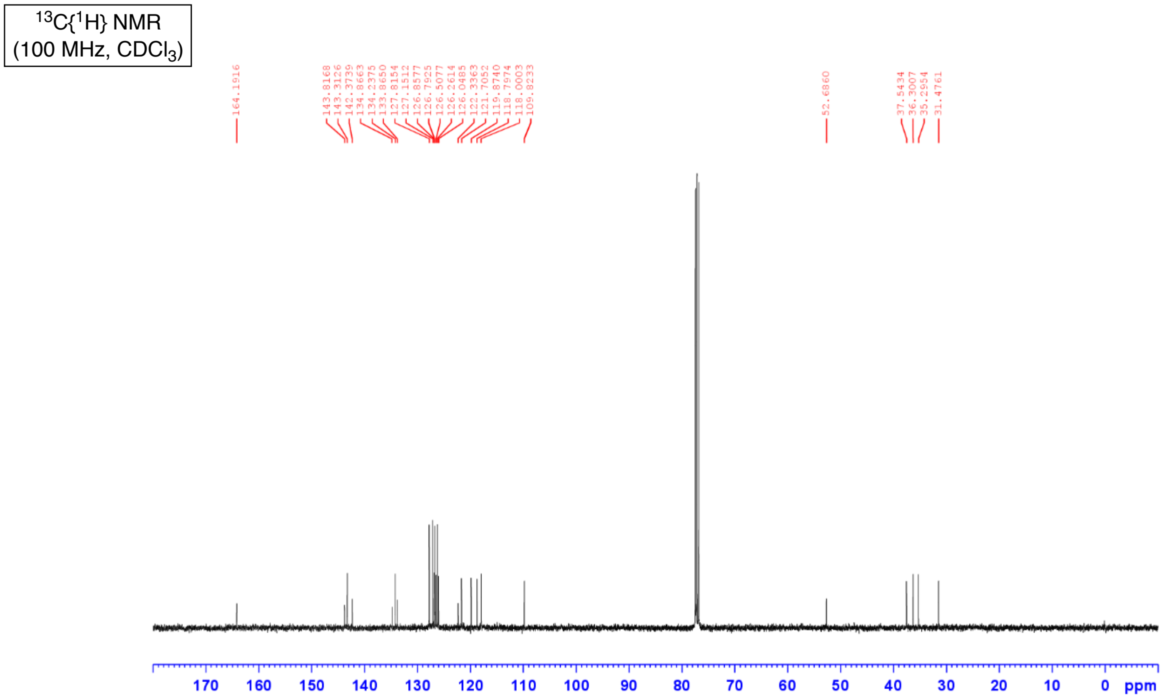

[^{1}H and ^{13}C{^{1}H} NMR Spectra of **3qa**]

^{1}H NMR
(400 MHz, CD_2Cl_2)

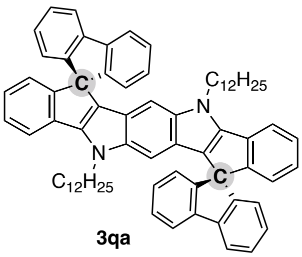

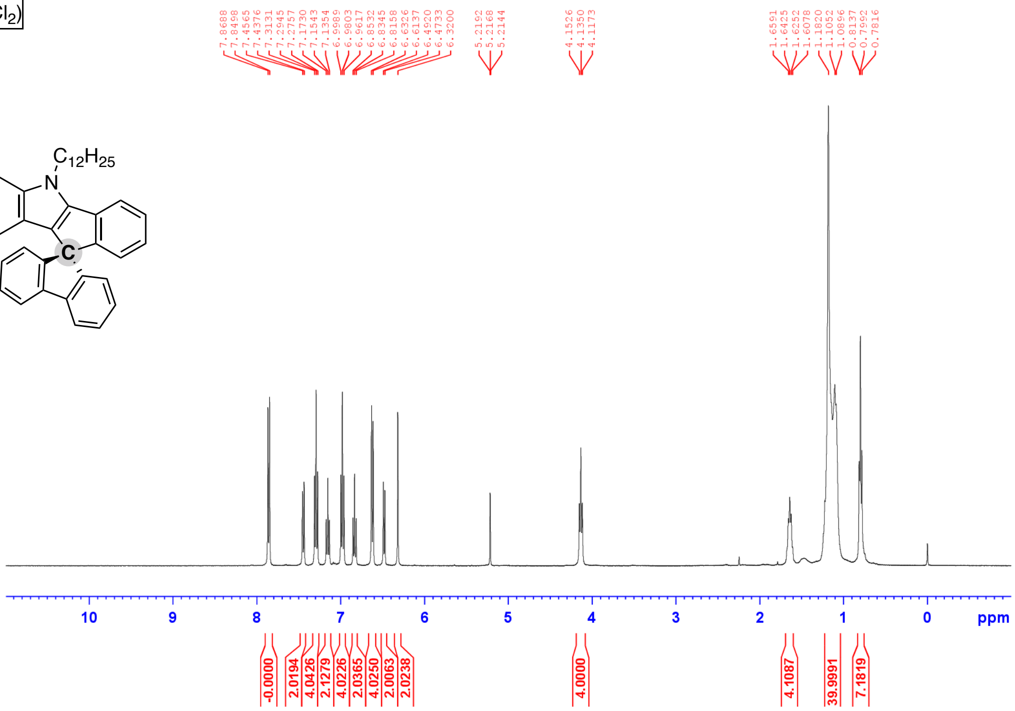

^{13}C{^{1}H} NMR
(100 MHz, CD_2Cl_2)

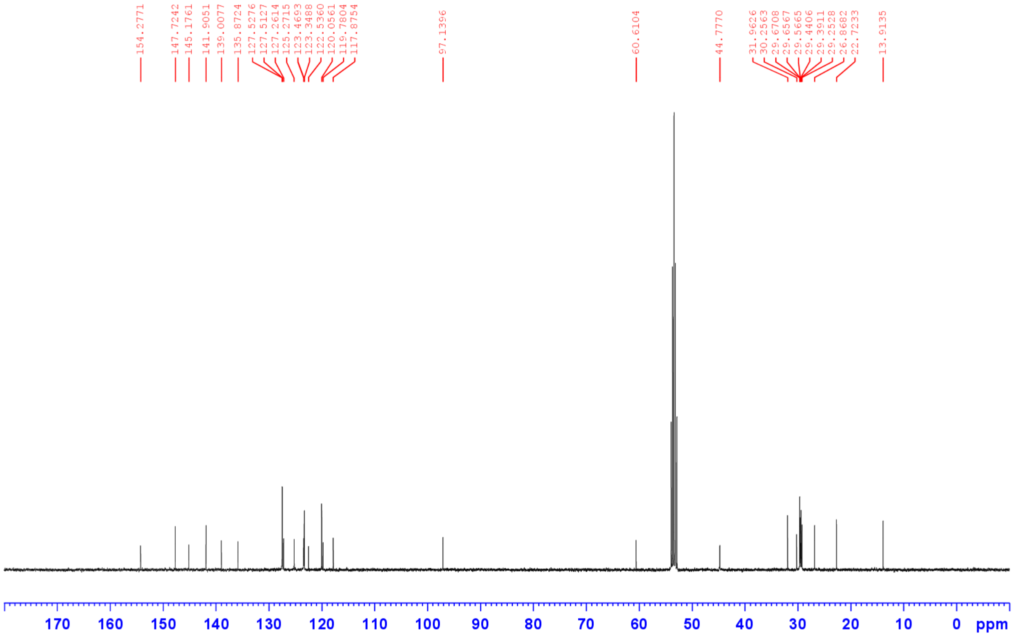

[^{1}H and ^{13}C{^{1}H} NMR Spectra of **3ra**]

^{1}H NMR
(400 MHz, $CDCl_3$)

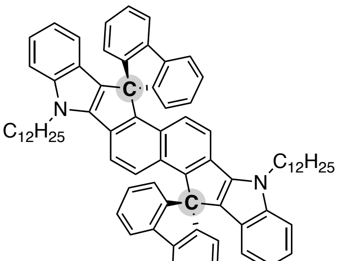

3ra

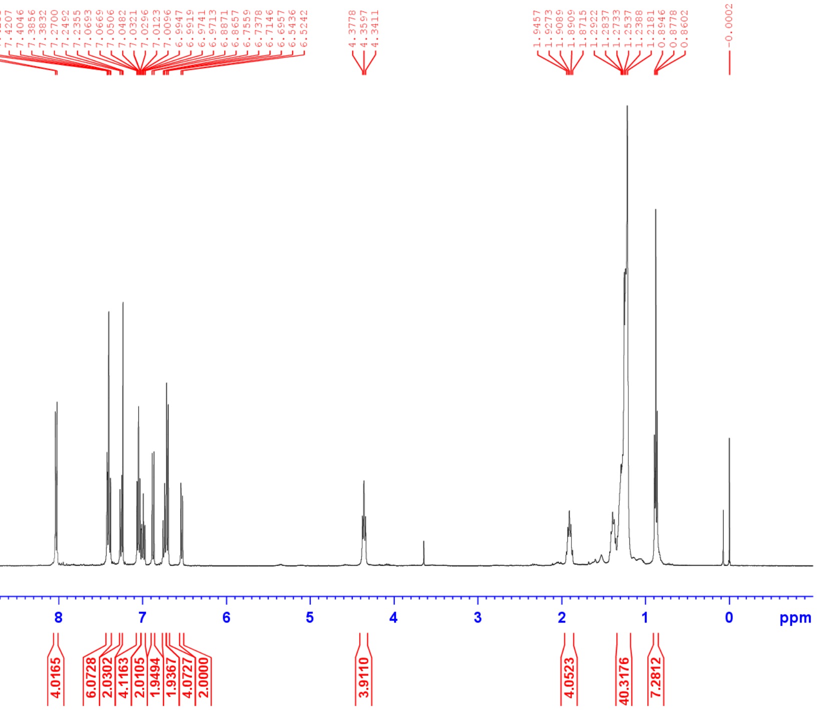

^{13}C{^{1}H} NMR
(100 MHz, $CDCl_3$)

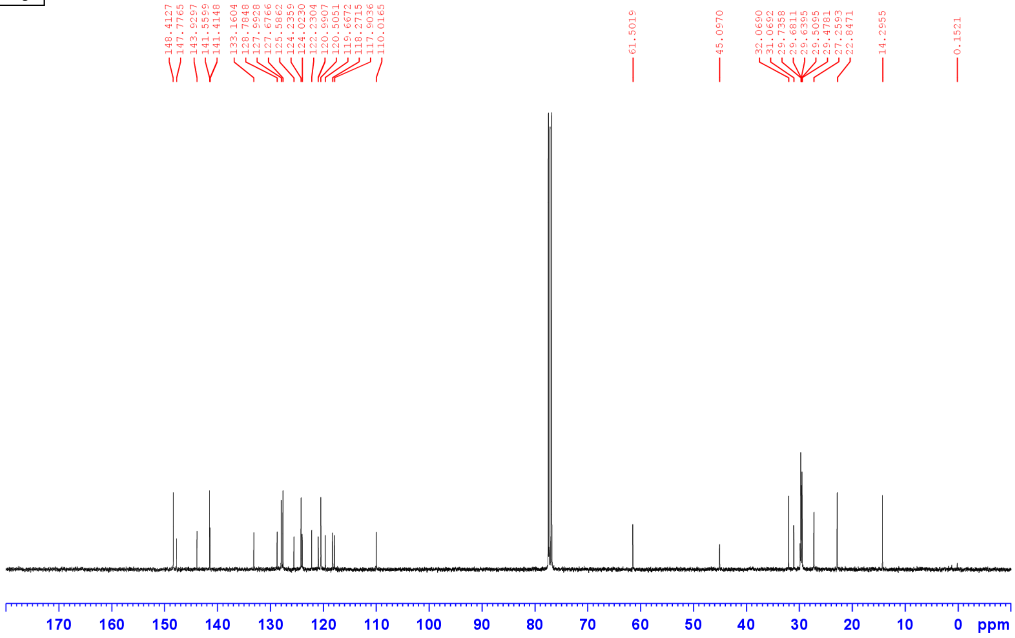

[^{1}H and ^{13}C{^{1}H} NMR Spectra of **3sa**]

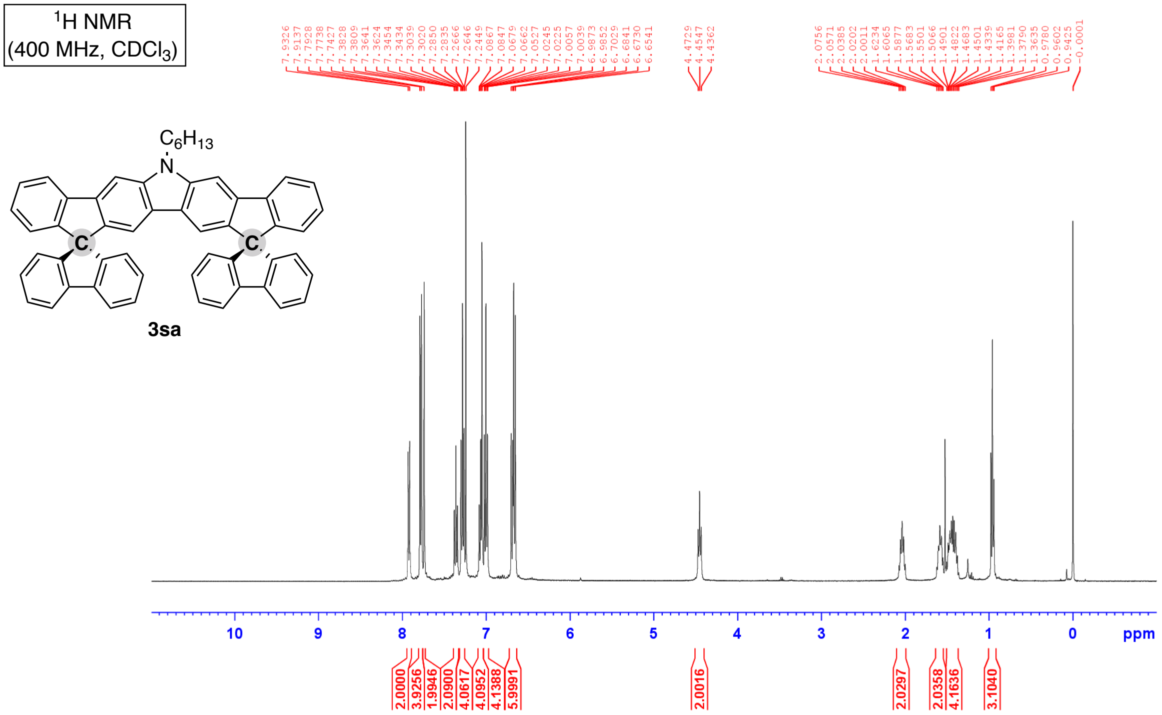

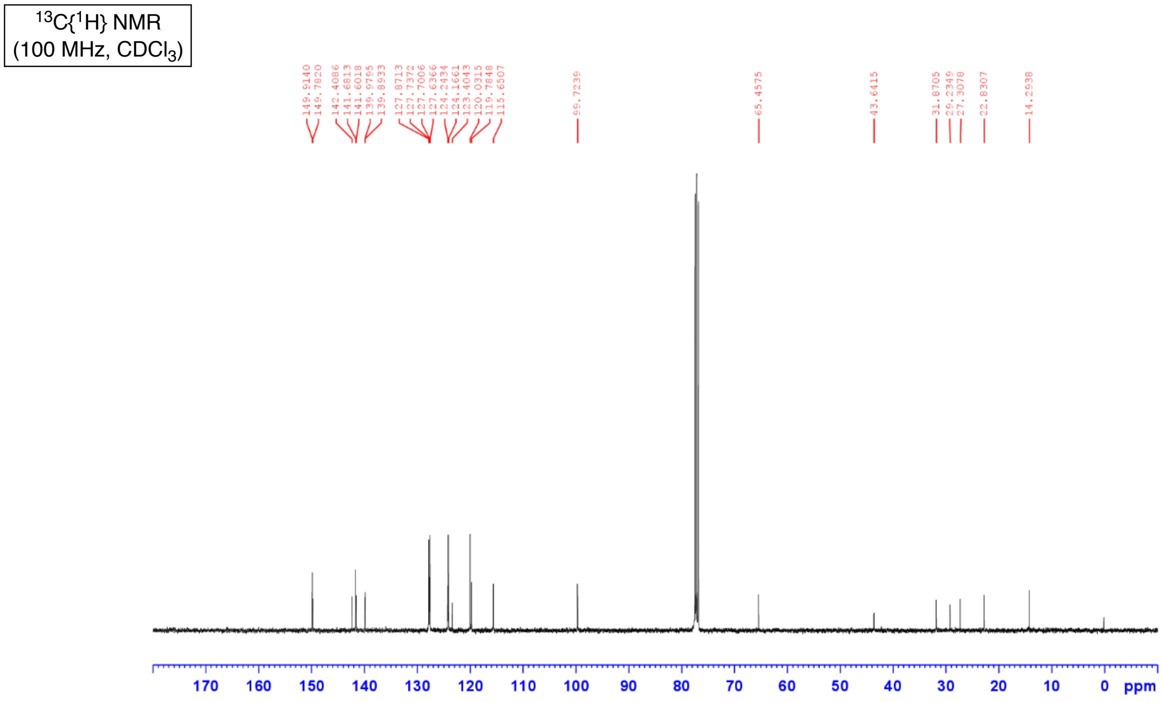

[^{1}H and $^{13}C\{^{1}H\}$ NMR Spectra of **3ta**]

^{1}H NMR
(400 MHz, $CDCl_3$)

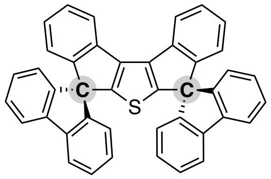

3ta

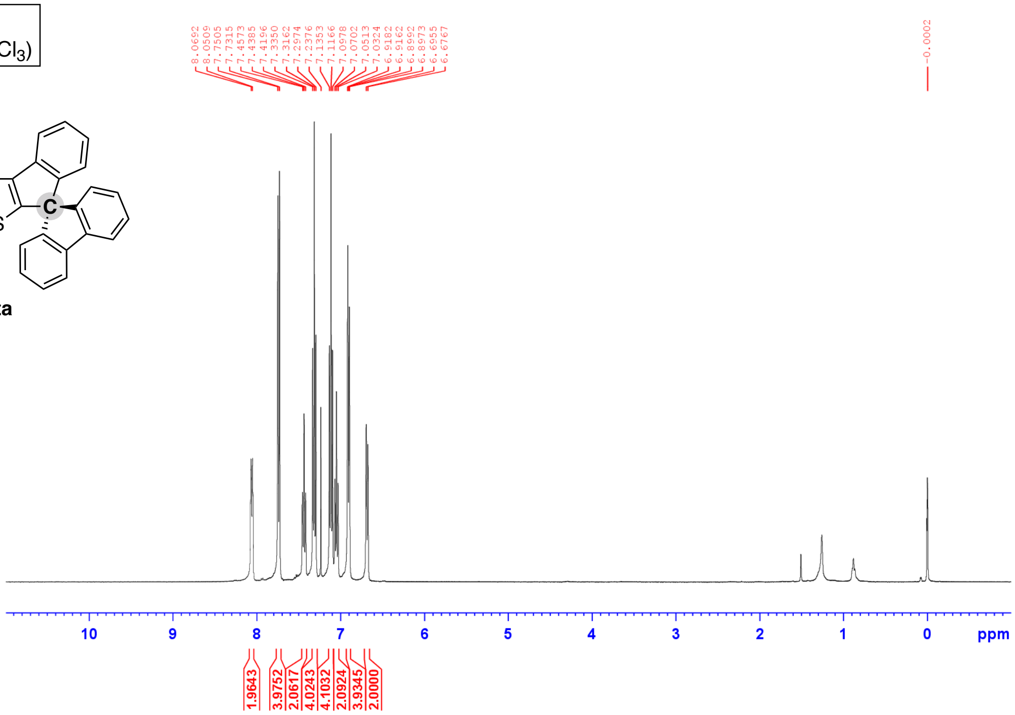

$^{13}C\{^{1}H\}$ NMR
(100 MHz, $CDCl_3$)

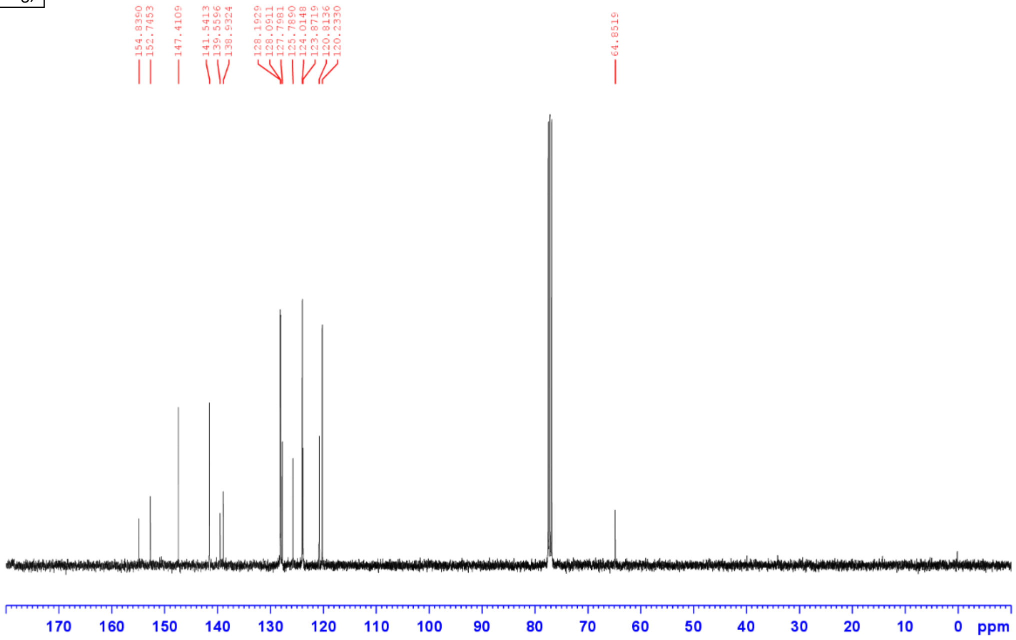

[^{1}H and $^{13}C\{^{1}H\}$ NMR Spectra of **3ua**]

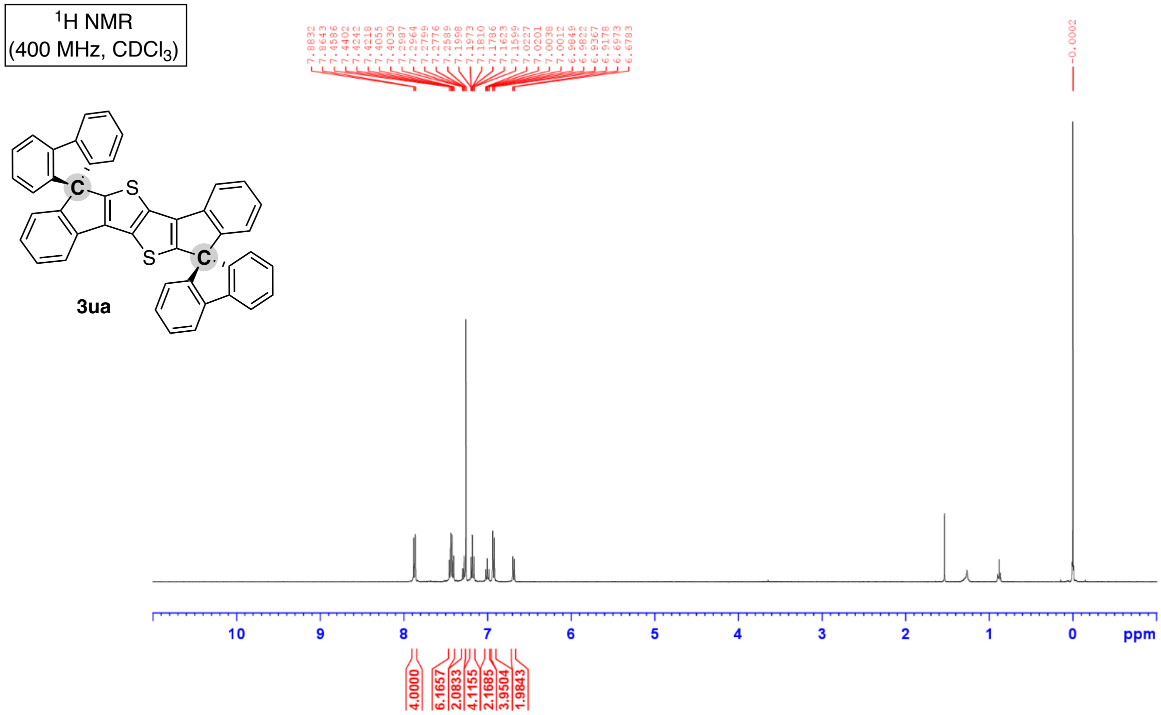

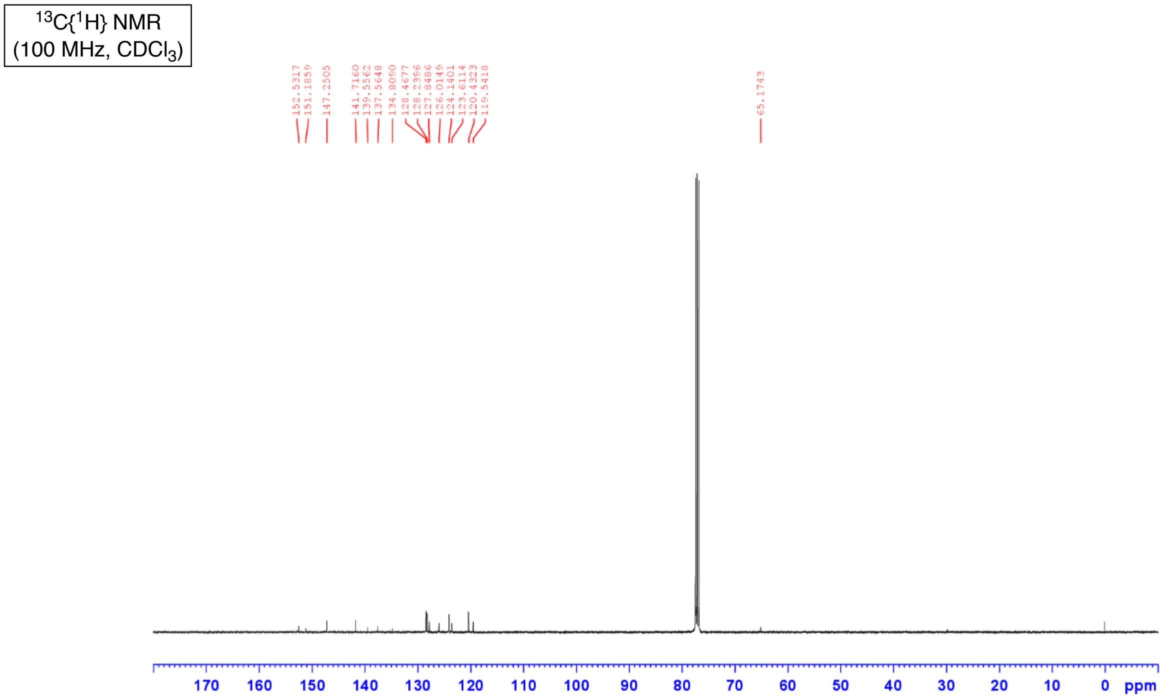

[^{1}H and ^{13}C{^{1}H} NMR Spectra of **3vd**]

^{1}H NMR
(400 MHz, $CDCl_3$)

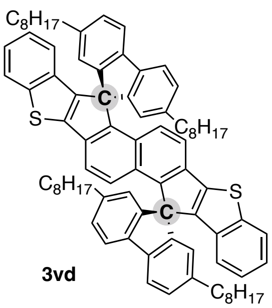

3vd

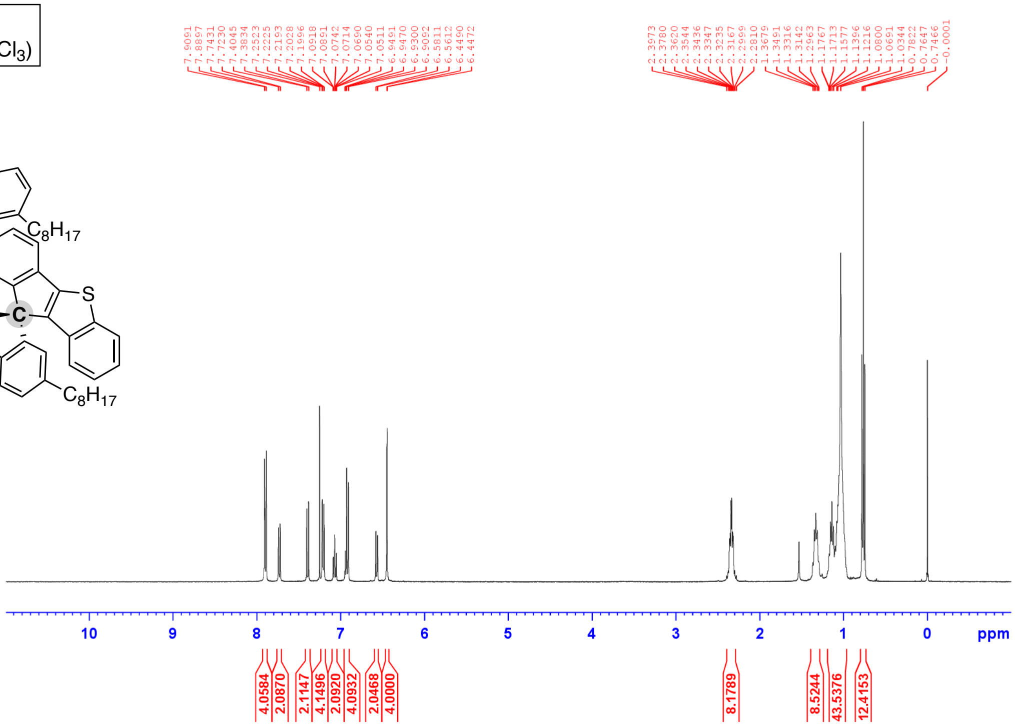

^{13}C{^{1}H} NMR
(100 MHz, $CDCl_3$)

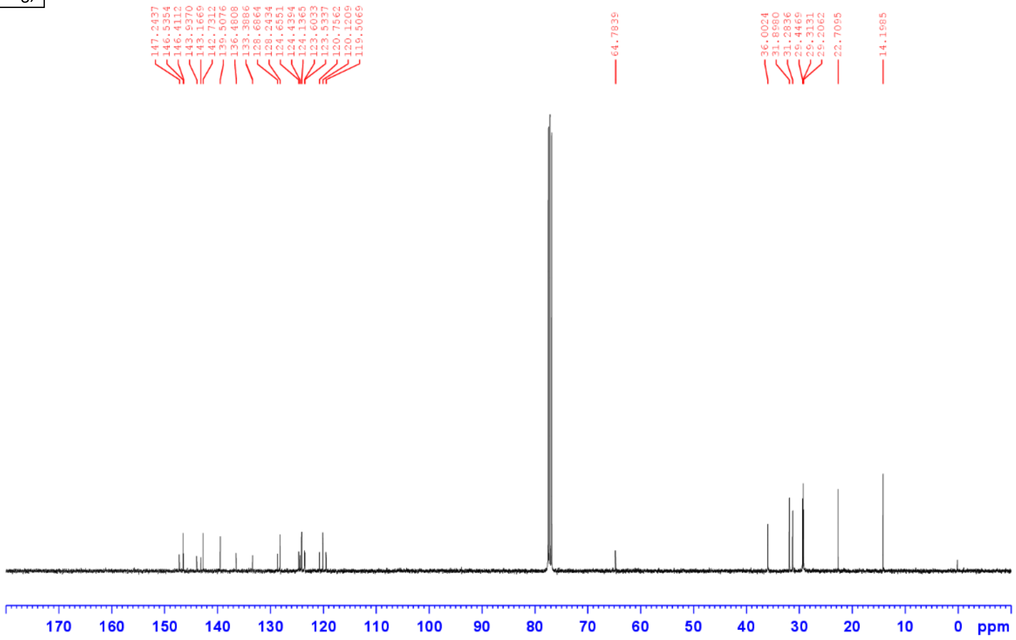

[^{1}H and ^{13}C{^{1}H} NMR Spectra of **3wd**]

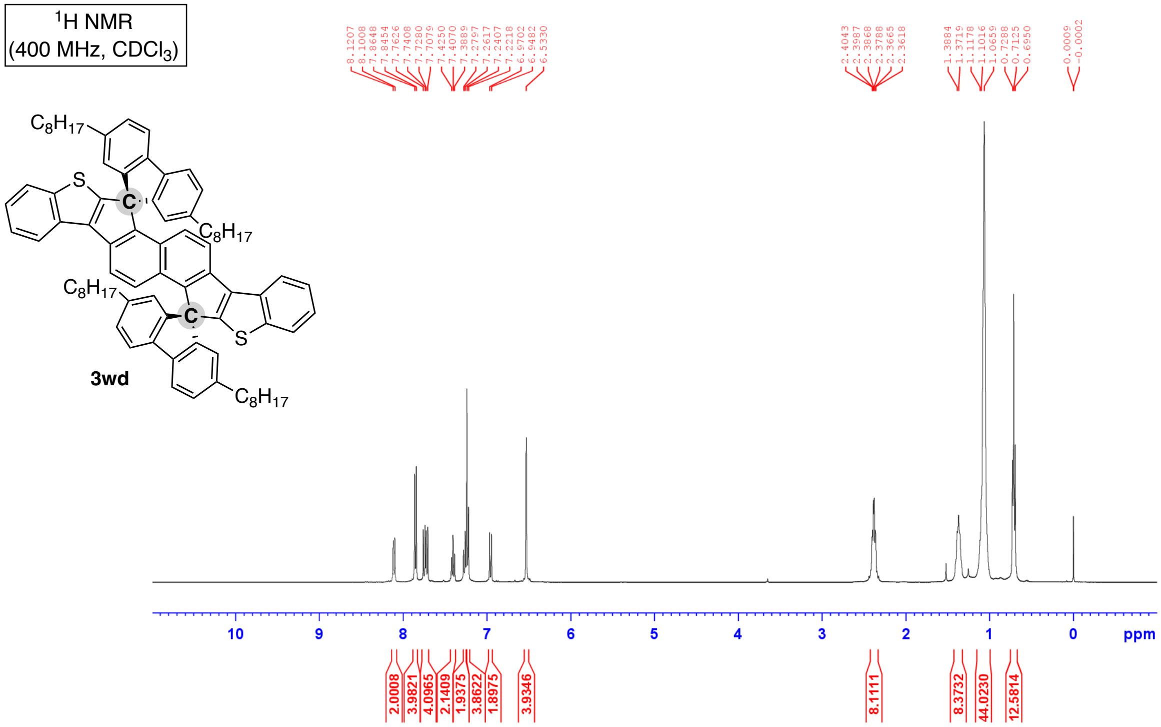

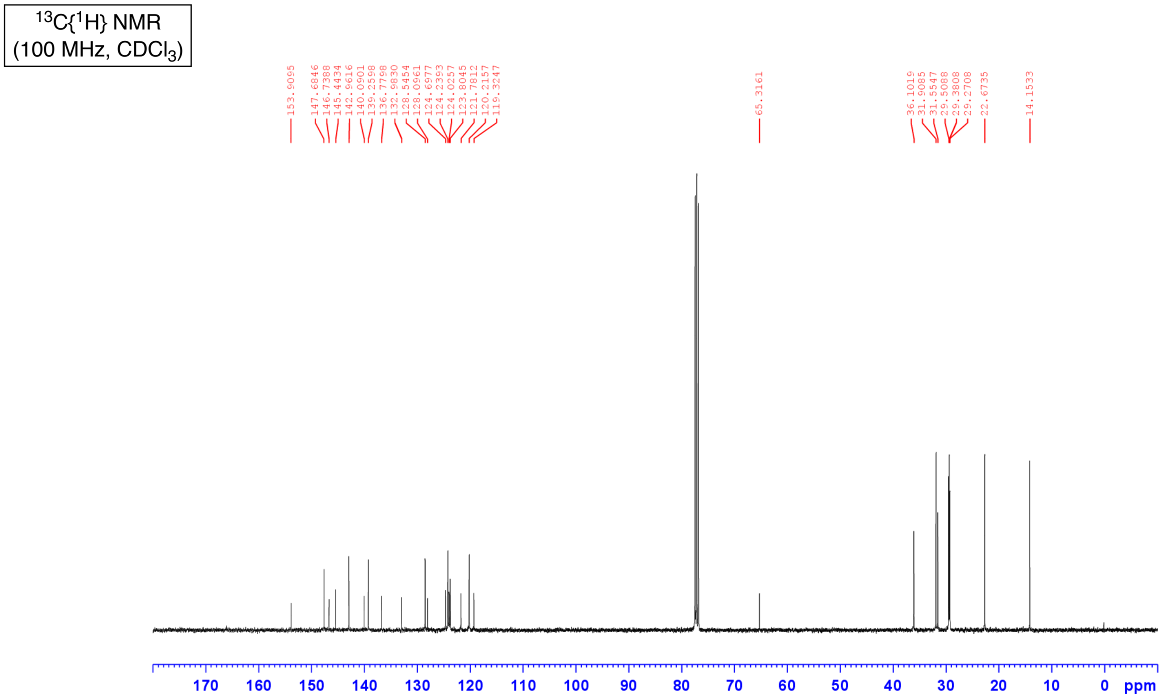

[^{1}H and ^{13}C{^{1}H} NMR Spectra of **3xa**]

^{1}H NMR
(400 MHz, $CDCl_3$)

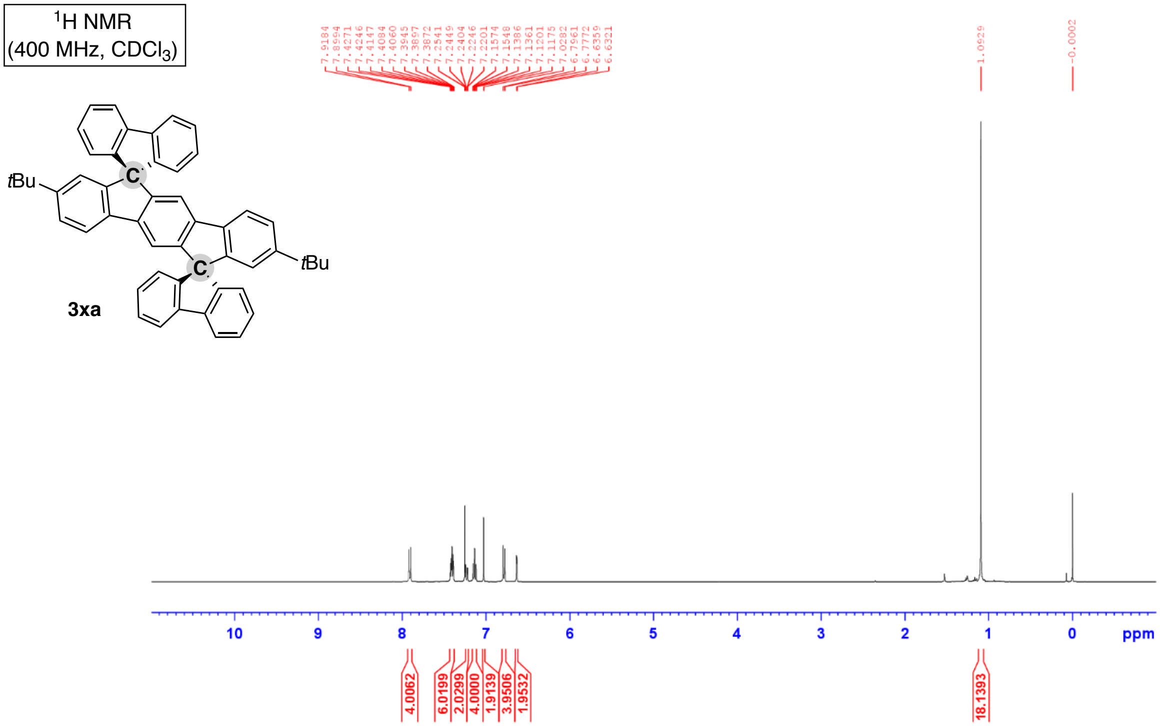

3xa

^{13}C{^{1}H} NMR
(100 MHz, $CDCl_3$)

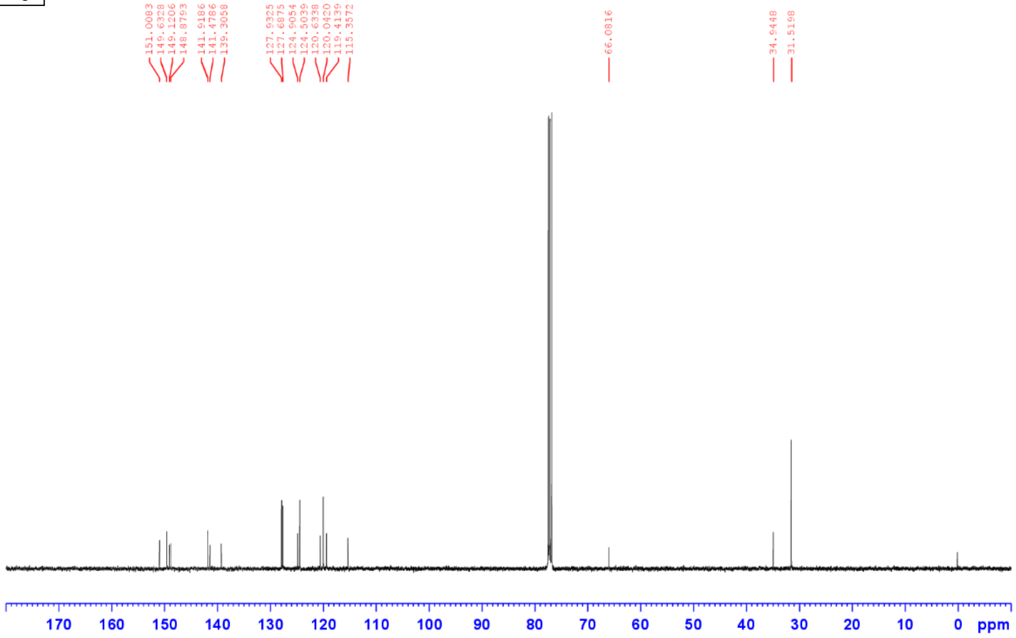

[^{1}H and ^{13}C{^{1}H} NMR Spectra of **3ya**]

^{1}H NMR
(400 MHz, $CDCl_3$)

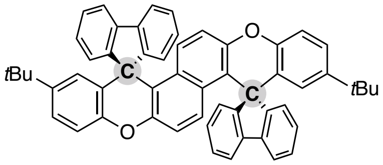

3ya

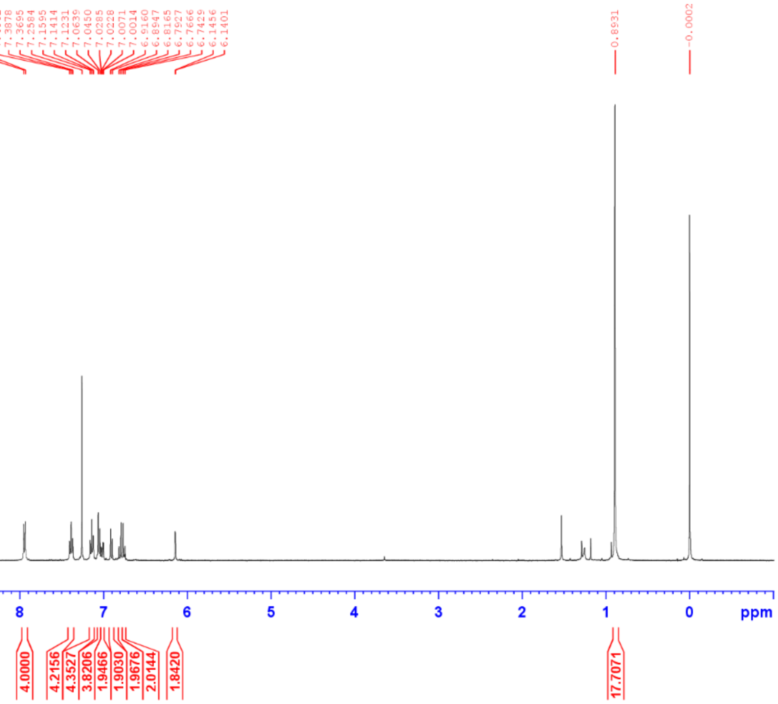

^{13}C{^{1}H} NMR
(100 MHz, $CDCl_3$)

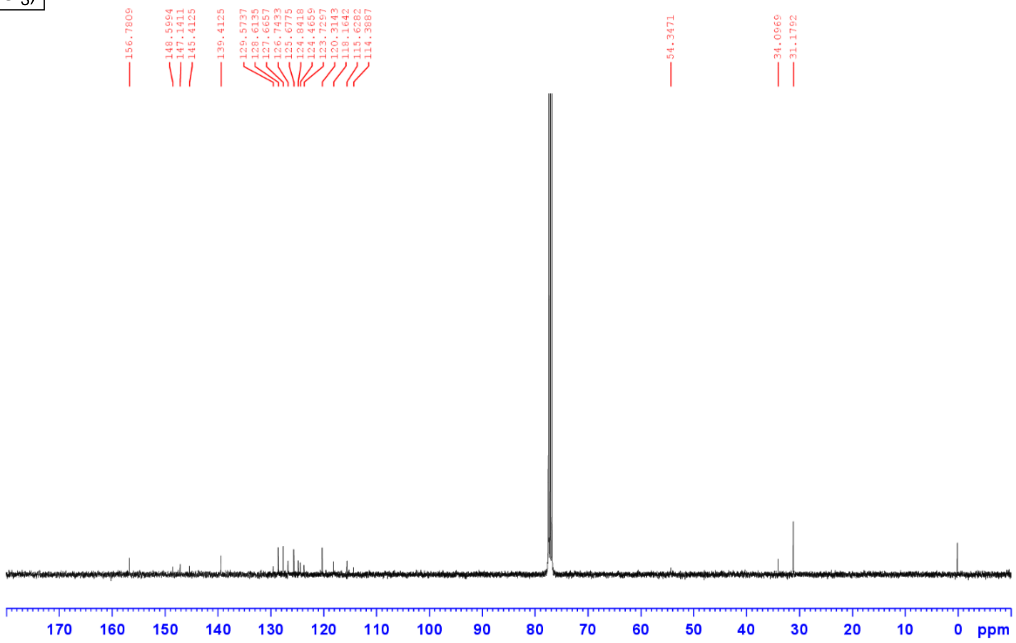

Supplement: SC-015-D3SC05977D-s001 [file SC-015-D3SC05977D-s001.pdf]
